# cPRC1.2 and CTCF-mediated transition from poised to active chromatin loops at bivalent genes

**DOI:** 10.1038/s44319-026-00885-3

**Published:** 2026-07-23

**Authors:** Aflah Hanafiah, Zhuangzhuang Geng, Tingting Liu, Yen Teng Tai, Wenjie Cai, Qiang Wang, Neil Christensen, Yan Liu, Feng Yue, Zhonghua Gao

**Affiliations:** 1 Department of Molecular and Precision Medicine, Penn State College of Medicine, Hershey, PA 17033 USA; 2 Penn State Hershey Cancer Institute, Hershey, PA 17033 USA; 3 Department of Biochemistry and Molecular Genetics, Feinberg School of Medicine, Northwestern University, Chicago, IL 60611 USA; 4 Center for Cancer Genomics, Feinberg School of Medicine, Northwestern University, Chicago, IL 60611 USA; 5 Department of Medicine, Feinberg School of Medicine, Northwestern University, Chicago, IL 60611 USA; 6 Department of Pathology and Laboratory Medicine, Penn State College of Medicine, Hershey, PA 17033 USA; 7 Faculty of Synthetic Biology, Shenzhen University of Advanced Technology, Shenzhen, Guangdong China; 8 Institute of Synthetic Biomedicine, Shenzhen University of Advanced Technology, Shenzhen, Guangdong, China

**Keywords:** Chromatin, Transcription & Genomics

## Abstract

Polycomb Repressive Complex 1 (PRC1) and CCCTC-binding factor (CTCF) are critical regulators of 3D chromatin architecture that influence cellular transcriptional programs. Although the role of CTCF in chromatin organization is well-known, the involvement of PRC1 is less understood. In this study, we identify an unexpected role for the canonical Pcgf2-containing PRC1 complex (cPRC1.2) in activating bivalent genes. Hi-C revealed that cPRC1.2 forms chromatin loops at bivalent promoters, rendering them poised for activation. Pcgf2 deletion disrupts cPRC1.2 loops and impairs the transcriptional induction of crucial target genes necessary for neuronal differentiation. Furthermore, we identify CTCF enrichment at cPRC1.2 loop anchors and at Polycomb group (PcG) bodies, suggesting that PRC1 and CTCF cooperatively regulate chromatin loops. Through virtual 4C and other genomic analyses, we discover that establishing neuronal progenitor cell (NPC) identity involves a switch from cPRC1.2-mediated chromatin loops to CTCF-mediated active loops. Our results suggest a novel mechanism by which pre-formed PRC1 loops at lineage-specific genes maintain a poised state for subsequent CTCF-mediated active loops and gene activation in cell fate transitions.

## Synopsis

The cPRC1.2 complex forms chromatin loops at poised bivalent promoters, and cell fate transition to neuronal progenitors involves a switch from cPRC1.2-mediated loops to CTCF-mediated active loops, enabling transcriptional activation of lineage-specific genes.

- The cPRC1.2 complex is required for formation of chromatin loops at bivalent promoters, poising them for activation during neuronal differentiation.
- Deletion of Pcgf2 disrupts these loops and impairs transcriptional induction of critical neuronal differentiation genes.
- CTCF is enriched at cPRC1.2 loop anchors and Polycomb bodies, and ESC-to-NPC differentiation is accompanied by a switch from cPRC1.2-mediated poised loops to CTCF-mediated active loops.

## Abstract (web summary)

The cPRC1.2 complex forms chromatin loops at poised bivalent promoters, and cell fate transition to neuronal progenitors involves a switch from cPRC1.2-mediated loops to CTCF-mediated active loops, enabling transcriptional activation of lineage-specific genes.

## Graphical abstract

**Figure:**
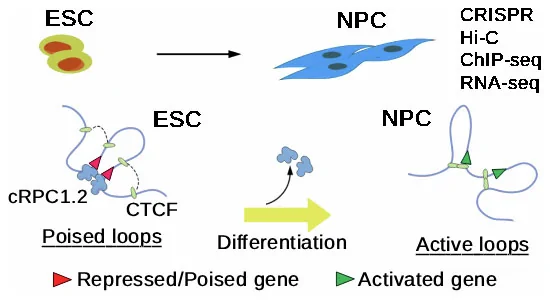

**Figure:**
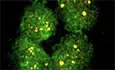

## Introduction

Epigenetic modulators, such as PcG proteins and CTCF, are central to regulating cellular transcriptional programs by shaping 3D chromatin architecture. Technological advances in high-throughput chromatin conformation capture techniques have led to the discovery of various levels of spatial organization within chromatin, including chromatin compartments, topologically associating domains (TADs), and long-range chromatin loops (Dixon et al, 2012; Nora et al, 2012; Sexton and Cavalli, 2015; Yu and Ren, 2017). In particular, chromatin loops constitute a dynamic, cell-type-specific regulation network (Phillips-Cremins et al, 2013). CTCF plays a pivotal role in forming chromatin loops through the coordination with cohesin complexes (Nichols and Corces, 2015; Phillips-Cremins et al, 2013). Recent studies have shown that other proteins, including PcG proteins, establish chromatin loops via distinct mechanisms (Eagen et al, 2017; Kondo et al, 2014; Kundu et al, 2017; Loubiere et al, 2020; Rhodes et al, 2020; Schoenfelder et al, 2015; Vieux-Rochas et al, 2015). However, the interplay between PcG proteins and CTCF in regulating 3D chromatin architecture and, consequently, cell type-dependent transcriptomes, remains not fully understood.

PcG proteins are critical epigenetic regulators that modulate chromatin structure to maintain gene silencing, essential for stem cell maintenance and differentiation (Jaenisch and Young, 2008; Margueron and Reinberg, 2011; Morey and Helin, 2010; Simon and Kingston, 2013). PcG proteins form two major protein complexes: Polycomb Repressive Complex 1 (PRC1) and 2 (PRC2) (Simon and Kingston, 2013). PRC1 and PRC2 catalyze two repressive chromatin modifications: mono-ubiquitination of histone H2A at lysine-119 (H2AK119ub1) and mono-, di-, and tri-methylation of histone H3 at lysine-27 (H3K27me1/2/3), respectively (Simon and Kingston, 2013). Previously, we determined the composition of the mammalian PRC1 complexes, identifying six groups (PRC1.1-1.6) based on the exclusive association of one of the six Polycomb group RING fingers (PCGF1-6) (Gao et al, 2012). Among these, PRC1.2 and PRC1.4 include the canonical PRC1 (cPRC1) complexes, initially isolated in *Drosophila*, and have homologous associated factors, including Really Interesting New Gene 1A/B (RING1A/B), Chromodomain proteins (CBX2/4/6/7/8), and Polyhomeotic homologs (PHC1/2/3) (Gao et al, 2012).

Genome-wide analyses of gene targets in ESCs revealed the localization of cPRC1 complexes at regulatory sites of many developmental transcription factors (TF) (Boyer et al, 2006; Bracken et al, 2006; Lee et al, 2006). Interestingly, subsequent studies discovered that H3K27me3, an inhibitory histone mark, and H3K4me3, an active mark, simultaneously bind promoters of these TFs (Bernstein et al, 2006). The bivalent nature of these genes, due to the enrichment and enzymatic action of PRC2 for H3K27me3 and mixed-lineage leukemia (MLL) for H3K4me3, maintains them at a silent yet poised state in ESCs (Bernstein et al, 2006). Upon lineage-specific differentiation, bivalent genes critical for that particular lineage undergo rapid activation, and those for other lineages remain repressed (Voigt et al, 2013). It has been shown that cPRC1 complexes, including cPRC1.2 and cPRC1.4, are also present at bivalent promoters through the interaction with their CBX subunits to H3K27me3 (Fischle et al, 2003; Ku et al, 2008; Min et al, 2003). However, it remains unclear how cPRC1 complexes regulate the plasticity of bivalent gene expression during differentiation.

PRC1-mediated H2AK119ub1 is a hallmark of silenced chromatin (Simon and Kingston, 2013). While most PCGF proteins enhance the E3 ubiquitin ligase activity of RING1B for H2AK119ub1 deposition, PCGF2 and PCGF4 are exceptions to this pattern (Taherbhoy et al, 2015). Removing Pcgf2 and Pcgf4 in mouse ESCs does not alter the overall levels of H2AK119ub1 (Fursova et al, 2019), suggesting that the cPRC1 complexes may regulate gene transcription through mechanisms independent of H2AK119ub1. Indeed, cPRC1 components have been shown to mediate long-range chromatin interactions in mammalian cells, and disruption of multiple cPRC1 complex components leads to the loss of these loops (Eagen et al, 2017; Kondo et al, 2014; Kundu et al, 2017; Loubiere et al, 2020; Rhodes et al, 2020; Schoenfelder et al, 2015; Vieux-Rochas et al, 2015). Microscopy analysis further demonstrates that distal PRC1 target genes are localized within PcG bodies (Bantignies et al, 2011; Lanzuolo et al, 2007)—nuclear foci enriched with PcG proteins (Alkema et al, 1997; Buchenau et al, 1998; Gunster et al, 1997; Hernandez-Munoz et al, 2005; Messmer et al, 1992; Schoorlemmer et al, 1997; Voncken et al, 1999)—which contribute to the repression of their target genes, underscoring the regulatory role of PRC1-mediated long-range chromatin in gene expression.

This study aims to understand how the PRC1 complex regulates long-range chromatin interactions to control gene expression during cell fate determination. Using CRISPR/Cas9-mediated gene editing, we deleted Pcgf2 in ESCs. We found that Pcgf2 absence unexpectedly compromises the activation of PRC1-targeted bivalent genes upon differentiation, in contrast to its traditional role as a transcriptional repressor. Mechanistically, through various genomic analyses, we demonstrated that Pcgf2 is required to form a subset of chromatin loops that target bivalent promoters. These promoters, marked by H3K4me3 and H3K27me3, are also bound by CTCF. Although previous studies and our immunoprecipitation experiments showed no direct physical interactions between PRC1 and CTCF, immunofluorescence analysis revealed their colocalization at PcG bodies, indicating cooperation between PRC1 and CTCF in regulating high-order chromatin interactions. Importantly, our virtual 4 C analysis showed that as ESCs differentiate into NPCs, PRC1 loops gradually diminish, and concomitantly, new chromatin loops mediated by CTCF emerge, many originating from former cPRC1.2 loop sites. Moreover, Pcgf2 deletion abolishes the CTCF-mediated active loops upon differentiation. Our findings uncover a potential mechanism by which PRC1 and CTCF coordinate chromatin loop reorganization, which might be essential for proper cell fate transition.

## Results

### Pcgf2 regulates neuronal differentiation independently of H2AK119ub1

**Figure EV1:**
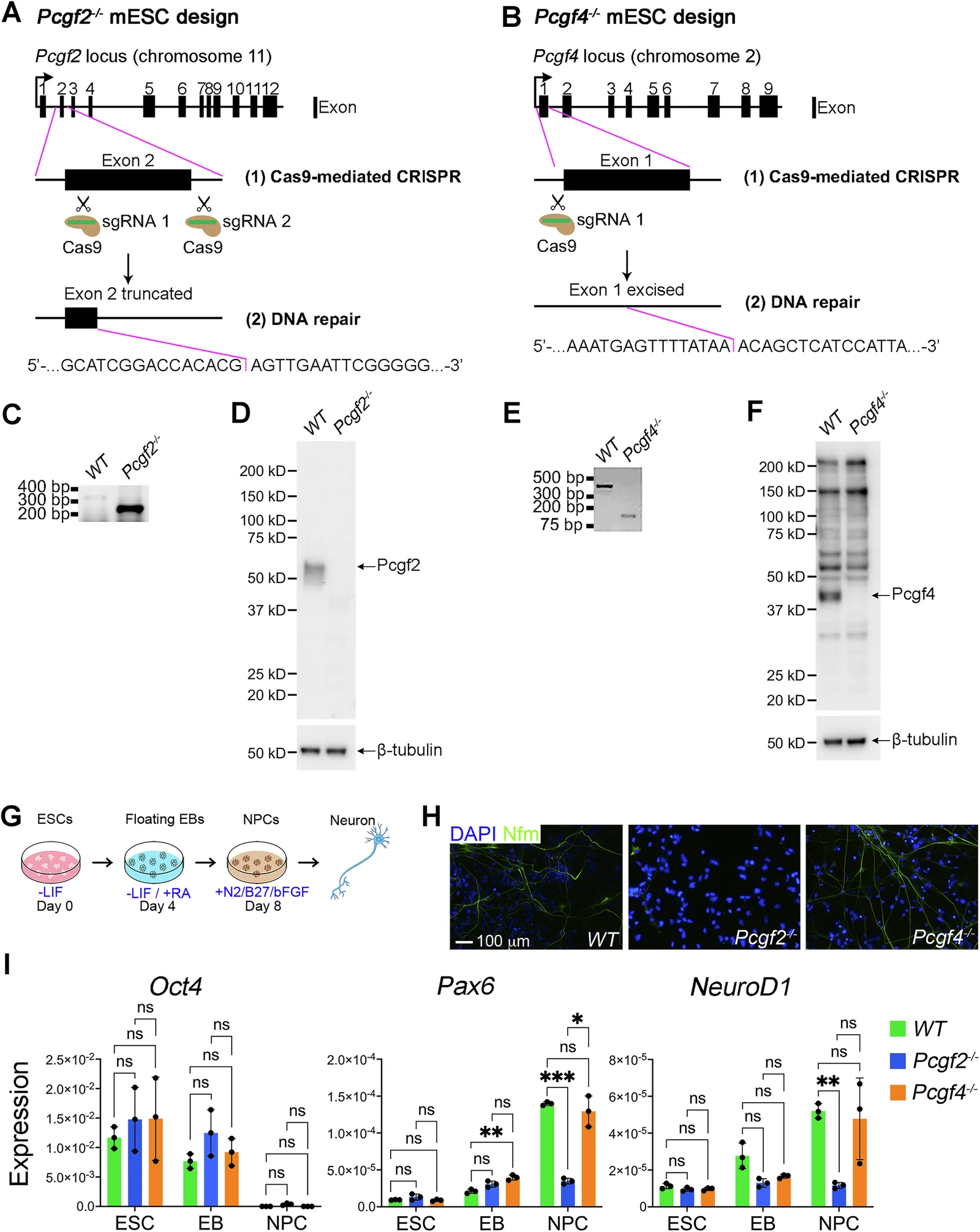
Generation and phenotypic analysis of Pcgf2^−/−^ and Pcgf4^−/−^ ESC lines. (**A**) Schematic of CRISPR/Cas9-mediated deletion of Pcgf2 in ESCs. Bottom: Sanger sequencing validating the excision of Exon 2. (**B**) Schematic of CRISPR/Cas9-mediated deletion of Pcgf4 in ESCs. Bottom: Sanger sequencing validating the excision of Exon 1. (**C**) Gel electrophoresis of PCR products using primers targeting the excision region in WT and Pcgf2^−/−^ ESC clone. (**D**) Immunoblotting of Pcgf2 in WT and Pcgf2^−/−^ ESC clone. (**E**) Gel electrophoresis of PCR products using primers targeting the excision region in WT and Pcgf4^−/−^ ESC clone. (**F**) Immunoblotting of Pcgf4 in WT and Pcgf4^−/−^ ESC clone. (**G**) Schematic of NPC differentiation, as detailed in the Methods section. (**H**) Immunofluorescence staining of Neurofilament (Nfm), a neuronal marker, in neurons differentiated from WT, Pcgf2^−/−^, Pcgf4^−/−^ ESCs. (**I**) RT-qPCR analysis of a pluripotency (*Oct4*) and NPC (*Pax6* and *NeuroD1*) markers in WT, Pcgf2^−/−^, and Pcgf4^−/−^ cells at the ESC, EB, and NPC stages, normalized to *Gapdh*. All mean values of expression levels and standard deviations (error bars) were calculated from three technical replicates. From left to right, *p* values are as following, for *Oct4*: 0.43, 0.76, 0.98, 0.29, 0.76, 0.50, 0.19, 0.76, 0.20; for *Pax6*: 0.24, 0.73, 0.19, 0.07, 0.008, 0.09, <0.001, 0.73, 0.04; for *NeuroD1*: 0.16, 0.33, 0.84, 0.11, 0.28, 0.27, 0.003, 0.77, and 0.27, calculated by Student’s *t*-test. *p < 0.05; **p < 0.01; ***p < 0.001; n.s. not significant.

To investigate the roles of PRC1 complexes in regulating chromatin architecture during cell fate transition, we generated knockout ESC lines for *Pcgf2* (*Pcgf2*^*−/−*^) and *Pcgf4* (*Pcgf4*^*−/−*^) via CRISPR/Cas9-mediated gene editing (Fig. EV1A,B). Genotyping PCR and immunoblotting confirmed the successful deletion of Pcgf2 and Pcgf4 (Fig. EV1C–F). Using a previously established in vitro neuronal differentiation protocol (Fig. EV1G) (Bibel et al, 2004; Hanafiah et al, 2020), we found that Pcgf2, but not Pcgf4, was required for neuronal differentiation, as evidenced by an immunofluorescence (IF) assay with a neurofilament (Nfm) antibody showing the defective dendritic growth in *Pcgf2*^*−/*^^−^ neurons compared with wild type (*WT*) and *Pcgf4*^*−/−*^ cells (Fig. EV1H). The failed neuronal differentiation in *Pcgf2*^*−/−*^ cells was also apparent at the NPC stage, with reduced expression of NPC marker genes, *Pax6* and *Neurod1*, as measured by RT-qPCR (Fig. EV1I).

**Figure EV2:**
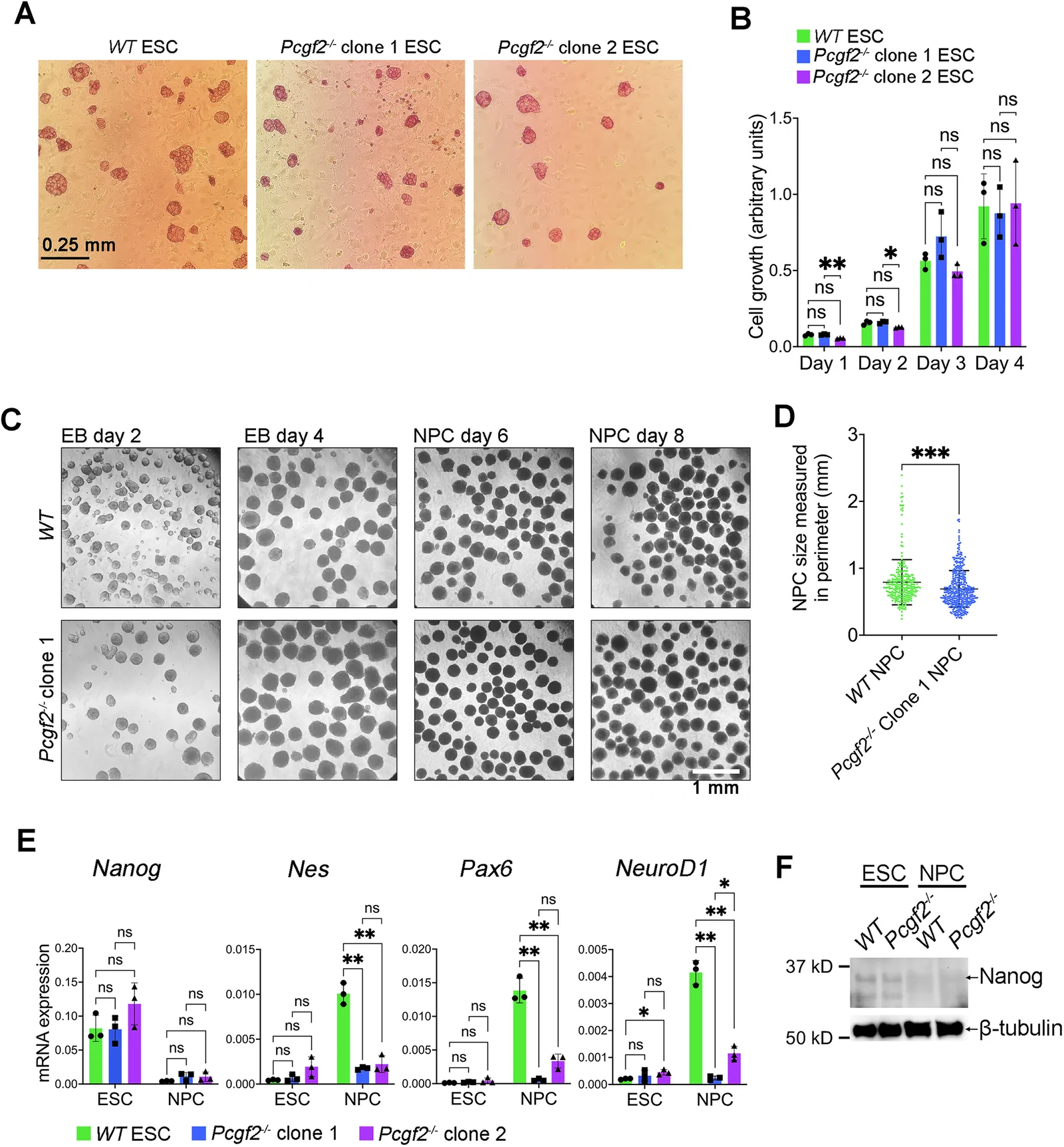
Characterization of Pcgf2^−/−^ ESC clones. (**A**) Alkaline phosphatase (AP) staining in WT and two Pcgf2^−/−^ ESC clones co-cultured with mouse embryonic fibroblasts (MEF). Pluripotent cells stain purple. (**B**) MTT cell proliferation assay in WT and two Pcgf2^−/−^ ESC clones over four days. All mean values and standard deviations (error bars) were calculated from three technical replicates. From left to right, *p* values are: 0.97, 0.08, 0.007, 0.97, 0.16, 0.03, 0.59, 0.30, 0.21, 0.97, 0.92, 0.74. A *t*-test was performed. *p < 0.05; **p < 0.01; n.s. not significant. (**C**) Brightfield images of the WT and Pcgf2^−/−^ clone 1 at EB and NPC stages during differentiation. (**D**) Quantification of NPC size at day 8 in WT (*n* = 277) and Pcgf2^−/−^ clone 1 (*n* = 416) based on cell perimeter. The center line represents the mean, and the ends represent the error bars from the standard deviation. A *t*-test was performed, yielding a *p* value <0.0001. ***p < 0.001. (**E**) RT-qPCR of pluripotency (*Nanog*) and NPC (*Nes*, *Pax6*, and *NeuroD1*) markers in WT and two Pcgf2^−/−^ clones at the ESC, EB, and NPC stages, normalized to *Gapdh*. All mean values of expression levels and standard deviations (error bars) were calculated from three technical replicates. From left to right, *p* values are as follows: for *Nanog*: 0.93, 0.32, 0.30, 0.25, 0.32, 0.89; for *Nes*: 0.40, 0.14, 0.32, 0.01, 0.002, 0.47; for *Pax*6: 0.13, 0.32, 0.45, 0.01, 0.005, 0.08; for *NeuroD1*: 0.41, 0.04, 0.40, 0.007, 0.003, 0.04. A *t*-test was performed. *p < 0.05; **p < 0.01; n.s. not significant. (**F**) Immunoblotting showing the Nanog protein level in WT and Pcgf2^−/−^ clone 1 ESCs and NPCs.

**Figure 1:**
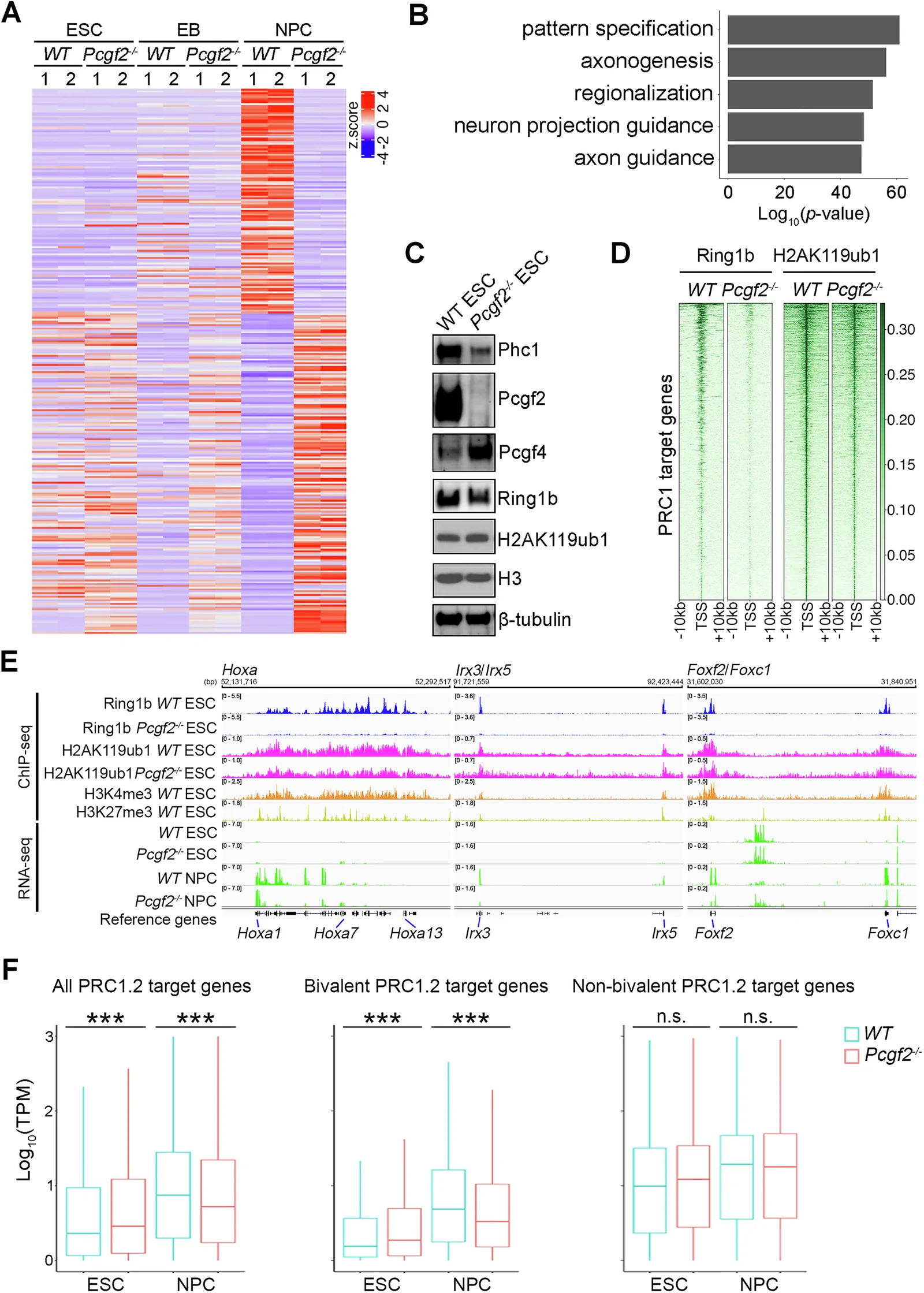
Pcgf2 is required for neuronal differentiation. (**A**) Heatmap of differentially expressed genes (DEGs) in duplicated *WT* and *Pcgf2*^*−/−*^ NPCs, clustered and sorted based on transcript z-score. A total of 2263 upregulated and 1576 downregulated genes were identified. (**B**) Gene ontology (GO) analysis for downregulated genes in *Pcgf2*^*−/−*^ NPCs from (**A**). *p* values are calculated by the hypergeometric distribution embedded in clusterprofiler package. (**C**) Immunoblotting of cPRC1 components (Phc1, Pcgf2, Pcgf4, and Ring1b) and H2AK119ub1 in *WT* and *Pcgf2*^*−/−*^ ESCs. (**D**) ChIP-seq heatmaps showing H2AK119ub1 and Ring1b enrichment in *WT* and *Pcgf2*^*−/−*^ ESCs at Ring1b (PRC1) target loci. (**E**) ChIP-seq tracks for Ring1b, H2AK119ub1, H3K4me3, and H3K27me3 in *WT* and *Pcgf2*^*−/−*^ ESCs and RNA-seq tracks for *WT* and *Pcgf2*^*−/−*^ ESCs and NPCs at PRC1 target loci (*Hoxa*, *Irx3*/*Irx5*, and *Foxf2*/*Foxc1*). (**F**) Box plots of expression levels for all PRC1.2 target genes (5334), bivalent PRC1.2 target genes (3284), and non-bivalent PRC1.2 target genes (2050). Here and below, boxes show the median (midline) and interquartile range (IQR), and whiskers extend either to the minimum or maximum data point or 1.5 X IQR beyond the box, whichever is shorter. Outliers are shown as dots. From left to right, *p* values are 0.00063, 1.5e-56, 0.00032, 3.0e-08, 0.88, and 0.89, calculated by Student’s *t*-test. ***p < 0.001; n.s. not significant. Source data are available online for this figure.

We then sought to determine whether the compromised neuronal differentiation in *Pcgf2*^*-/-*^ cells was due to an impact on ESC self-renewal. Through alkaline phosphatase assay, we demonstrated that Pcgf2 deletion did not affect the ESC pluripotency in two independent *Pcgf2*^−^^*/−*^ ESC lines, compared with *WT* (Fig. EV2A). Similarly, there was no noticeable difference in cell proliferation in these *Pcgf2*^*−/−*^ ESC lines, compared with *WT*, via MTT assay (Fig. EV2B). Morphologically, when *Pcgf2*^*−/−*^ ESCs were induced for NPC differentiation, we found no dramatic change in embryonic body (EB) shape but a slight reduction in EB size (Fig. EV2C,D). Immunoblotting and RT-qPCR analysis showed that Pcgf2 deletion does not affect *Nanog*, a pluripotency marker (Fig. EV2E,F). These data indicate that Pcgf2 deletion has no significant effect on ESC self-renewal and proliferation. Next, to investigate how the loss of Pcgf2 affects gene transcription during NPC differentiation, we performed RNA-seq analysis on *WT* and *Pcgf2*^*−/−*^ cells across the ESC, EB, and NPC stages. Although canonical PRC1 complexes are traditionally viewed as transcriptional repressors, certain variants of PRC1 can also activate transcription. Consistent with the dual role of PRC1 complexes, our differential gene expression analysis revealed bidirectional changes in gene expression in *Pcgf2*^*−/−*^ NPCs (Fig. 1A). To gain insight into the potential activation function of PRC1.2, we focused on genes that are downregulated in the absence of Pcgf2 for further analysis. Gene ontology (GO) analysis revealed that genes failed to be induced in *Pcgf2*^*−/−*^ NPCs are primarily involved in cell differentiation and neuronal identity, including “pattern specification”, “axonogenesis”, “regionalization”, “neuron projection guidance”, and “axon guidance” (Fig. 1B).

To examine whether the observed transcriptomic changes by Pcgf2 deletion were caused by the compromised integrity of the PRC1 complex, we analyzed protein levels of PRC1 components by immunoblotting. Pcgf2 loss reduced the level of Phc1, a cPRC1 component (Fig. 1C), indicating that the Pcgf2 deletion caused the reduced stability of the cPRC1.2 complex but not the non-canonical PRC1 (ncPRC1) complexes. Ring1b, the E3 ligase for H2AK119ub1 and the common component of all PRC1 complexes, showed only a minor reduction in total protein levels in *Pcgf2*^*−/−*^ cells (Fig. 1C), but the genomic enrichment of Ring1b was decreased (Fig. 1D). However, consistent with previous findings (Fursova et al, 2019), global H2AK119ub1 level was not substantially affected in *Pcgf2*^*−/−*^ cells, evidenced by both immunoblotting and ChIP-seq analysis (Fig. 1C,D), suggesting a potential H2AK119ub1-independent mechanism mediating the effect by Pcgf2 loss. Interestingly, Pcgf2 deletion resulted in Pcgf4 overexpression, possibly compensating for Pcgf2 loss (Fig. 1C), but unable to rescue neuronal differentiation defects caused by Pcgf2 knockout (Fig. 1A,B). Altogether, our results reveal an H2AK119ub1-independent role for PRC1.2 in regulating NPC differentiation.

### Pcgf2 deletion compromises activation of PRC1.2-targeted bivalent genes upon neuronal differentiation

Previous studies have shown that PRC1 complexes localize at bivalent promoters (Asenjo et al, 2023; Ku et al, 2008). We sought to investigate whether PRC1.2 plays a role in regulating bivalent gene expression during neuronal differentiation. We first identified bivalent loci simultaneously marked by H3K27me3 and H3K4me3. We then cross-referenced these loci with Ring1b and Pcgf2 binding sites on the genome, identifying a total of 3,284 PRC1.2-targeted bivalent promoters. Three examples are shown for the *Hoxa*, *Irx3/Irx5*, and *Foxf2/Foxc1* loci (Fig. 1E). These PRC1.2 target genes are kept silent in *WT* ESCs, and Pcgf2 deletion only mildly increases their expression (Fig. 1E), which is consistent with a previous report (Fursova et al, 2019). When neuronal differentiation was initiated, we observed dramatic induction of these genes in *WT* NPCs, in keeping with their roles in neurodevelopment as previously described (Aldinger et al, 2009; Dou et al, 2021; Goncalves et al, 2020), and Pcgf2 deletion inhibited their activation (Fig. 1E). This is somewhat surprising given the traditional roles of PRC1 as transcriptional repressors. Globally, PRC1.2 target genes tend to be less activated in *Pcgf2*^*−/−*^ NPCs compared to *WT* NPCs (Fig. 1F). Interestingly, when we divided PRC1.2 target genes based on their bivalency status, we found that non-bivalent PRC1.2 target genes are less subjected to alteration by Pcgf2 knockout compared to the bivalent PRC1.2 target genes (Fig. 1F), suggesting that the PRC1.2 complex specifically target bivalent genes for their activation upon neuronal differentiation.

### cPRC1.2 mediates long-range chromatin interactions

**Figure 2:**
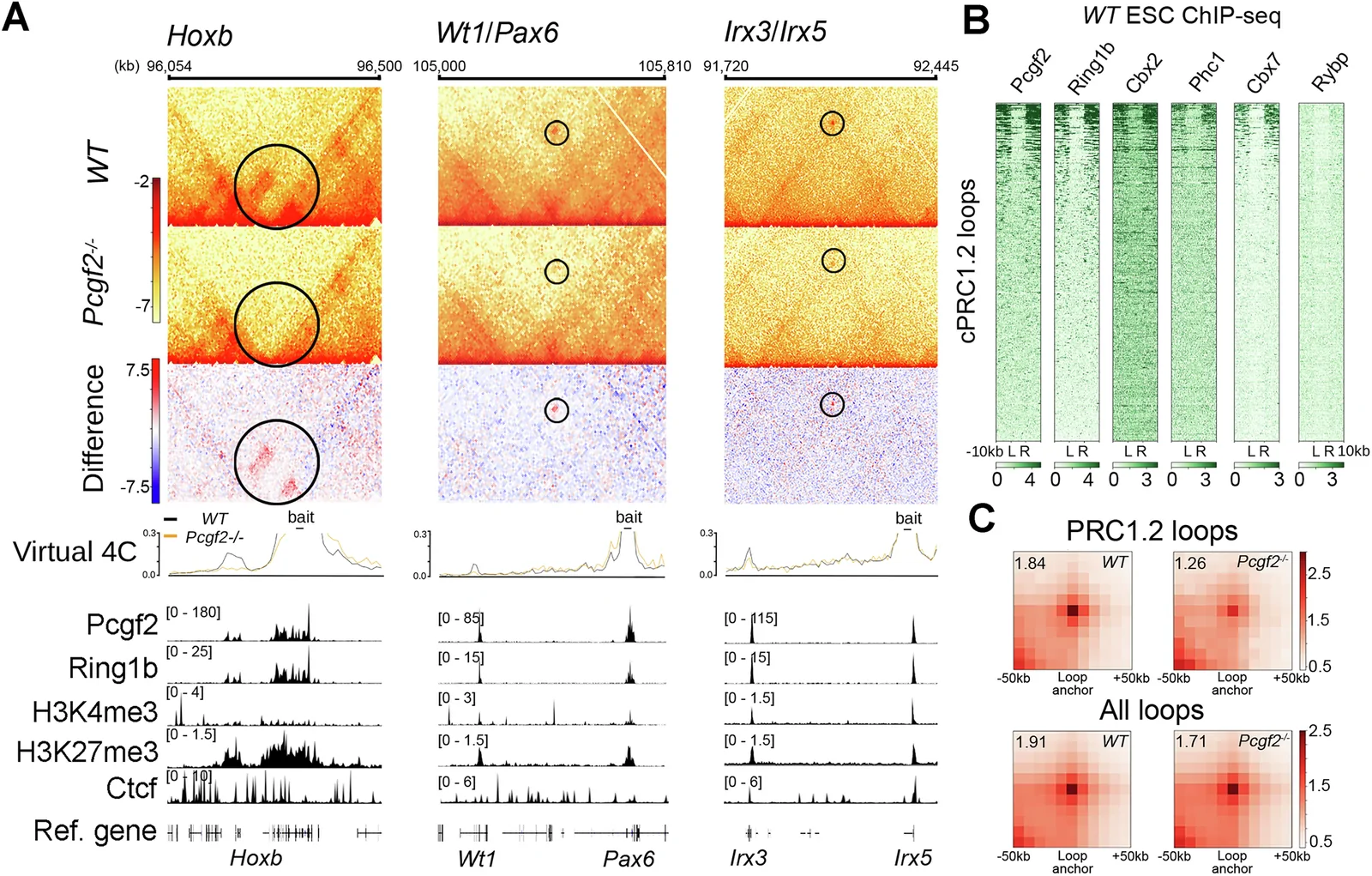
cPRC1.2 is required for chromatin loop formation. (**A**) Example Hi-C matrices for *WT* and *Pcgf2*^*−/−*^ ESCs at 5-kb resolution showing reduced contact intensities at *Hoxb*, *Wt1*/*Pax6*, and *Irx3*/*Irx5* loci in *Pcgf2*^*−/−*^ ESCs. Virtual 4C plots are constructed using Hi-C data. The bait was set at one of the two loop anchors as indicated. ChIP-seq tracks for Pcgf2, Ring1b, H3K4me3, H3K27me3, and Ctcf are displayed below the Hi-C matrices. (**B**) ChIP-seq heatmaps show enrichment of cPRC1.2 components (Pcgf2, Cbx2, Phc1, and Cbx7) and lack of ncPRC1 component (Rybp) at anchors of 432 cPRC1.2 loops in *WT* ESCs. L and R denote left and right loop anchors, with 10 kb regions flanking each anchor. (**C**) Aggregate peak analysis (APA) of Hi-C for total chromatin loops and cPRC1.2 loops in *WT* and *Pcgf2*^*−/−*^ ESCs at 10-kb resolution. The pileups are normalized to the average of the top-left and bottom-right corner pixels, and the value of the central pixels is displayed on the top-left side of the plot. A total of 3912 loops and 149 cPRC1.2 loops with Pcgf2 anchored on both sides were analyzed.

Previous studies have demonstrated that PRC1 complexes regulate long-range chromatin interactions independently of Ring1b catalytic activity toward H2AK119ub1. Furthermore, Phc1, a component essential for cPRC1 oligomerization (Isono et al, 2013; Kim et al, 2002), has been shown to influence long-range chromatin interactions at selective loci in ESCs (Kundu et al, 2017). Despite the previously established role of PRC1 in chromatin looping, the specific subcomplex remains undefined. Given that Pcgf2 deletion had little effect on H2AK119ub1 but notably reduced Phc1 level (Fig. 1C), we speculated that the observed dysregulation of bivalent genes in *Pcgf2*^*−/−*^ NPCs was mediated by the cPRC1.2 complex through long-range chromatin interactions. To test this hypothesis, we performed Hi-C analysis in *WT* and *Pcgf2*^*−/−*^ ESC samples. Deep-sequenced raw data were mapped to the mouse genome GRCm38 to detect ligation junctions (Appendix Table S1). Although no significant change in A/B compartments and TADs in *Pcgf2*^*−*^^*/−*^ ESCs compared to *WT* (Appendix Fig. S1A–D), our bioinformatic analysis identified 3912 chromatin loops in *WT* ESCs; among them, 458 are not present in *Pcgf2*^*−/−*^ ESCs (Appendix Fig. S2A). We define cPRC1.2-dependent loops as those anchored at genomic sites with enriched Pcgf2 in *WT* ESCs and disrupted in *Pcgf2*^*−/−*^ ESCs. From this analysis, a total of 432 PRC1.2 loops were identified, with 149 of those localized by Pcgf2 on both anchors and 283 localized by Pcgf2 on one anchor (Appendix Fig. S2A). Figure 2A shows three selected examples, including *Hoxb*, *Wt1*/*Pax6*, and *Irx3*/*Irx5* loci, with substantially reduced contact intensities in the *Pcgf2*^*-/-*^ Hi-C matrix compared to *WT*. Pcgf2 and Ring1b, two core components of the cPRC1.2 complex, are enriched at the genomic anchors of these loops (Fig. 2A). The *Hoxb* and *Pax6* loci have been shown in previous studies to be targeted by loops dependent on PRC1 (Kundu et al, 2017). Further ChIP-seq analysis revealed that the cPRC1.2 components, such as Pcgf2, Ring1b, Cbx2, Phc1, and Cbx7, are all enriched at loop anchors, but Rybp, an ncPRC1 component, is absent (Fig. 2B; Appendix Fig. S2B). Among all genes targeted by cPRC1.2 loops (Appendix Table S2), many are associated with pathways that regulate neuronal development (Appendix Fig. S2C).

Aggregate peak analysis (APA), which measures average loop strength, revealed a global reduction in PRC1.2-associated chromatin loops in *Pcgf2*^*−/−*^ ESCs (Fig. 2C, upper panel). Although CTCF is localized to these loop anchoring regions (Fig. 2A), there is no noticeable difference in total chromatin loops between *WT* and *Pcgf2*^*−/−*^ ESCs (Fig. 2C, lower panel), suggesting that the effect of Pcgf2 deletion is restricted to the selected loops targeted by PRC1.2. Interestingly, these PRC1.2 loops are often anchored at sites with enrichment of H3K4me3 and H3K27me3, suggesting a bivalent status of these regions (Fig. 2A). In summary, our results demonstrate that the cPRC1.2 complex is responsible for the establishment of a specific set of chromatin loops.

### cPRC1.2 loops target bivalent genes for activation upon neuronal differentiation

**Figure 3:**
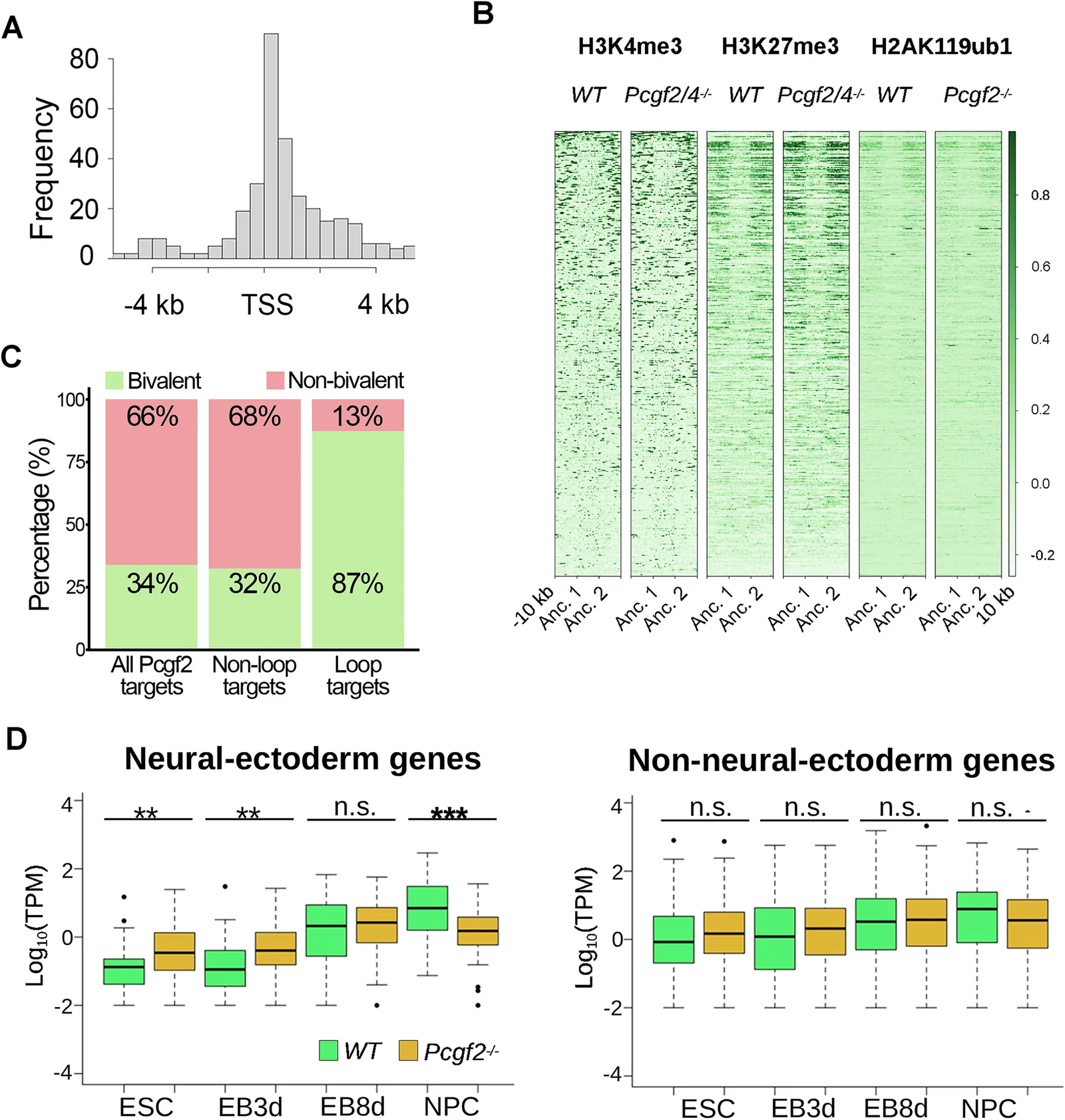
cPRC1.2 loops target bivalent promoters for gene activation upon differentiation. (**A**) Distribution of cPRC1.2 loop regions relative to the nearest annotated transcription start site (TSS). The x-axis represents the distance to TSS. (**B**) Heatmaps show ChIP-seq signals of H3K4me3 and H3K27me3 enrichment across cPRC1.2 loop regions in *WT* and *Pcgf2/4*^*−/−*^ ESCs, and H2AK119ub1 enrichment across cPRC1.2 loop regions in *WT* and *Pcgf2*^*−/−*^ ESCs. (**C**) Bar graph shows the percentage of bivalent domains within total Pcgf2 targets (8194 total, 2794 bivalent, 5400 non-bivalent), non-loop Pcgf2 targets (7933 total, 2566 bivalent, 5367 non-bivalent), and cPRC1.2 loop targets (261 total, 228 bivalent, 33 non-bivalent). (**D**) Box plots showing expression of neural-ectoderm genes (77) and non-neural-ectoderm genes (194) that are targeted by cPRC1.2 loops in *WT* and *Pcgf2*^*−/−*^ cells across ESC, EB, and NPC stages. Boxes show the median (midline) and interquartile range, and whiskers extend to the minimum or maximum data point, or to 1.5 × interquartile range beyond the box, whichever is shorter. Outliers are shown as dots. From left to right, *p* values are 0.0080, 0.0051, 0.35, 0.00072, 0.21, 0.34, 0.85, and 0.29, calculated by Student’s *t*-test. **p < 0.01; *** *p* < 0.001; n.s. not significant.

Our observation of bivalent histone marks at selected cPRC1.2-mediated chromatin loop anchors (Fig. 2A) suggests the potential involvement of these loops in regulating bivalent genes. Therefore, we conducted a further bioinformatic analysis to assess the genome-wide relationship between the cPRC1.2-mediated chromatin loops and bivalency. As shown in Fig. 3A, cPRC1.2-mediated chromatin loops are primarily anchored at transcription start sites (TSS). More importantly, cPRC1.2 loop anchors are found to be enriched with H3K4me3 and H3K27me3 modifications (Fig. 3B). Strikingly, our analysis showed that PRC1 loops exclusively target bivalent regions, with a high percentage (87%) of cPRC1.2 loops-targeted regions being bivalent, compared with 34% of all PRC1-targeted and 32% of non-loop forming PRC1-targeted regions showing bivalency (Fig. 3C). Additionally, only 10.4% (697 out of 6694) of cPRC1.2-independent loop anchors being localized by Pcgf2, H3K4me3, and H3K27me3 (Appendix Fig. S3A). From this analysis, we calculated that of all 8194 Pcgf2 occupied genomic sites, 261 of them reside at loops that are disrupted upon Pcgf2 deletion, and 1584 of them are associated with loops not affected by Pcgf2 deletion. These results suggest that cPRC1.2 loops specifically target bivalent promoters.

In our transcriptomic analysis, we discovered a surprisingly reduced expression of bivalent genes targeted by cPRC1.2 in *Pcgf2*^*−/−*^ NPCs (Fig. 1E,F). Given the specific targeting of bivalent promoters by cPRC1.2 loops, we then examined how these chromatin loops may affect the activation of bivalent genes targeted by PRC1. Of all 271 genes targeted by Pcgf2 loops, 166 (61.3%) of them are dysregulated in Pcgf2-deficient NPCs (Appendix Fig. S3B). Specifically, we found that 50.6% (39 out of 77 genes) of the neural-ectoderm genes are downregulated, 16.9% (13 out of 77 genes) of the neural-ectoderm genes are upregulated, and 32.5% (25 out of 77 genes) of the neural-ectoderm genes have unchanged expression. Meanwhile, there is almost an equal distribution of meso/endoderm cPRC1.2 genes that are downregulated, upregulated, and unchanged (Appendix Fig. S3B). Although no difference was found in the average transcript per million (TPM) values for all PRC1 target genes and cPRC1.2 loop-targeted genes between *WT* and *Pcgf2*^*−/−*^ NPCs (Appendix Fig. S3C), Pcgf2 deletion led to a significant reduction in TPM for neuro-ectodermal genes targeted by the cPRC1.2 loops in *Pcgf2*^*−/−*^ NPCs (Fig. 3D; Appendix Fig. S3D). Interestingly, either non-neuro-ectodermal or meso-endodermal genes targeted by the cPRC1.2 loops showed no noticeable difference in their expression between *WT* and *Pcgf2*^*−/−*^ NPCs (Fig. 3D; Appendix Fig. S3C), indicating that cPRC1.2 loops are selectively required for the activation of neuro-ectodermal genes in our neuronal differentiation model. When we omitted retinoic acid (RA) at day 4 and let EB differentiate without restriction into NPC in the same differentiation period (EB8d), the observed difference in expression of neuro-ectodermal genes targeted by cPRC1.2 loops was diminished (Fig. 3D). It is worth noticing that, at the ESC stage, the Pcgf2 deletion causes a de-repression of neuro-ectodermal genes targeted by cPRC1.2 loops but has no noticeable effect on non-neuro-ectodermal or meso-endodermal genes (Fig. 3D; Appendix Fig. S3C). Our results suggest that the cPRC1.2 complex plays a role in activating neuro-ectodermal lineage genes through chromatin looping to promote NPC identity.

### Phc1 SAM domain deletion mimics Pcgf2 knockout effects on cPRC1.2 target gene expression

**Figure 4:**
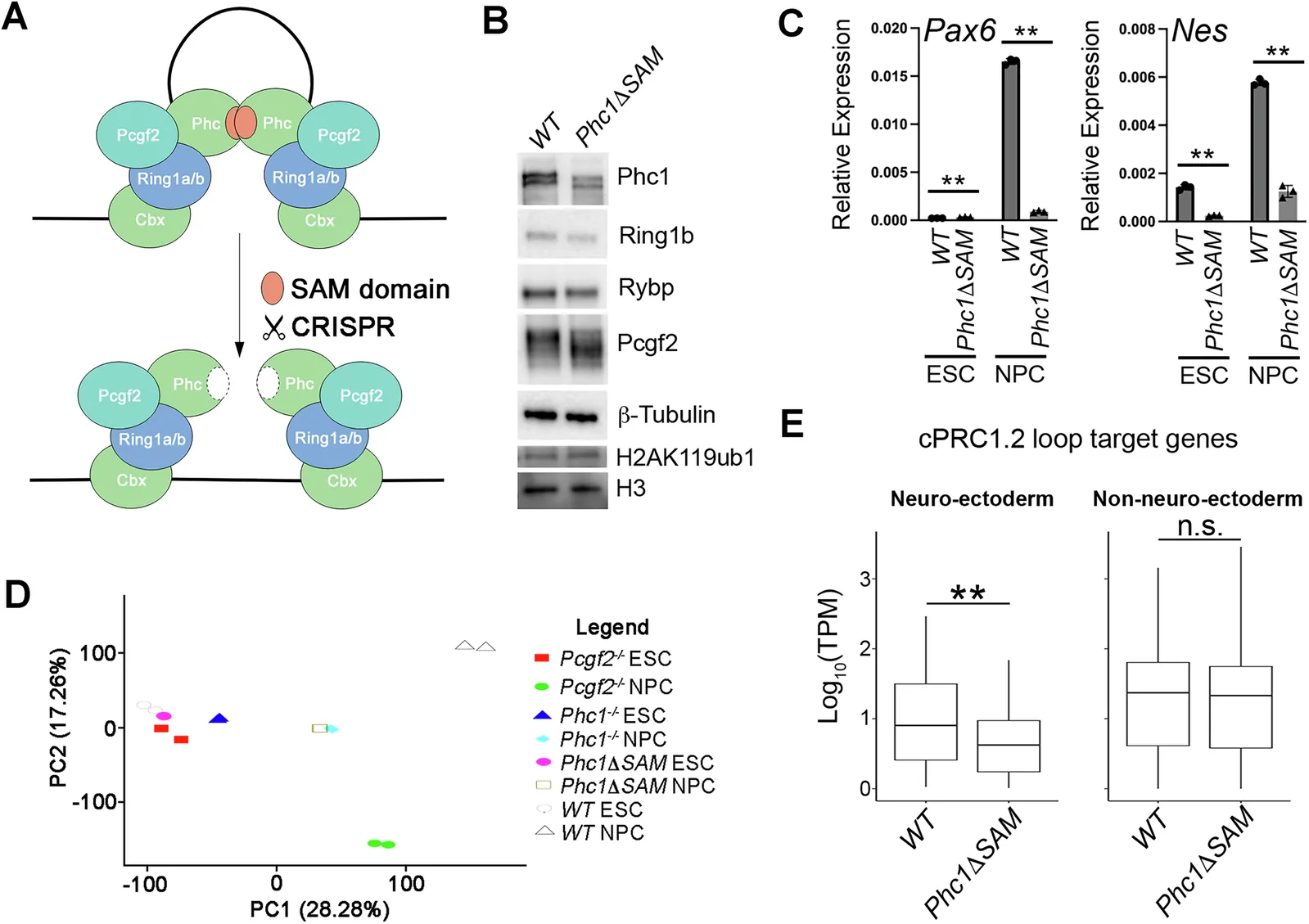
Deleting Phc1 SAM domains compromises target gene activation in NPCs. (**A**) Schematic of Phc1 SAM domain deletion (*Phc1∆SAM*) for disrupting cPRC1 oligomerization. (**B**) Immunoblotting of Phc1, Ring1b, Rybp, Pcgf2, and H2AK119ub1 in *WT* and *Phc1∆SAM* ESCs. Note the smaller size of the Phc1 band in the *Phc1∆SAM* sample. (**C**) RT-qPCR analysis showing failed induction of NPC markers (*Pax6* and *Nes*) in *WT* and *Phc1∆SAM* NPCs. All mean values of expression levels and standard deviations (error bars) were calculated from three technical replicates. From left to right, *p* values are 0.0070, 1.1e-7, 5.2e-5, and 1.0e-5, calculated by Student’s *t*-test. **p < 0.01. (**D**) Principal component analysis (PCA) of RNA-seq data from *WT*, *Pcgf2*^*−/−*^, *Phc1*^*−/−*^, and *Phc1∆SAM* ESCs and NPCs. (**E**) Box plots of expression of neuro-ectodermal (77) and non-neuro-ectodermal (194) genes targeted by cPRC1.2 loops in *WT* and *Phc1∆SAM* NPCs. Boxes show the median (midline) and interquartile range, and whiskers extend to the minimum or maximum data point, or to 1.5 × interquartile range beyond the box, whichever is shorter. From left to right, *p* values are 0.0052 and 0.26, calculated by Student’s *t*-test. **p < 0.01; n.s. not significant. Source data are available online for this figure.

Pcgf2 is present in both cPRC1 and ncPRC1 (Gao et al, 2012; Morey et al, 2013; Tavares et al, 2012). To further evaluate the specific contribution of cPRC1.2-mediated loops in controlling the expression of bivalent genes, we generated an ESC line with the deletion of Phc1 sterile alpha motif (*Phc1∆SAM*) (Fig. 4A; Appendix Fig. S4A). Phc proteins are components specific for cPRC1 (Gao et al, 2012). Among all three Phc paralogs, Phc1 is the predominant form in ESCs (Kloet et al, 2016), and the *Phc1* deletion in ESCs disrupted selective chromatin loops (Kundu et al, 2017). It has been previously shown that the SAM domain of Phc mediates PRC1 oligomerization and PRC1 clustering in cells (Isono et al, 2013; Kim et al, 2002). The *Phc1∆SAM* ESC line has been confirmed by Sanger sequencing, genotyping PCR, and immunoblotting (Appendix Fig. S4A,B; Fig. 4B, note the smaller size of *Phc1∆SAM*). The SAM domain deletion has no noticeable effect on global protein levels in PRC1 components, including Ring1b, Pcgf2, Rybp, and PRC1-mediated modification H2AK119ub1 (Fig. 4B).

**Figure EV3:**
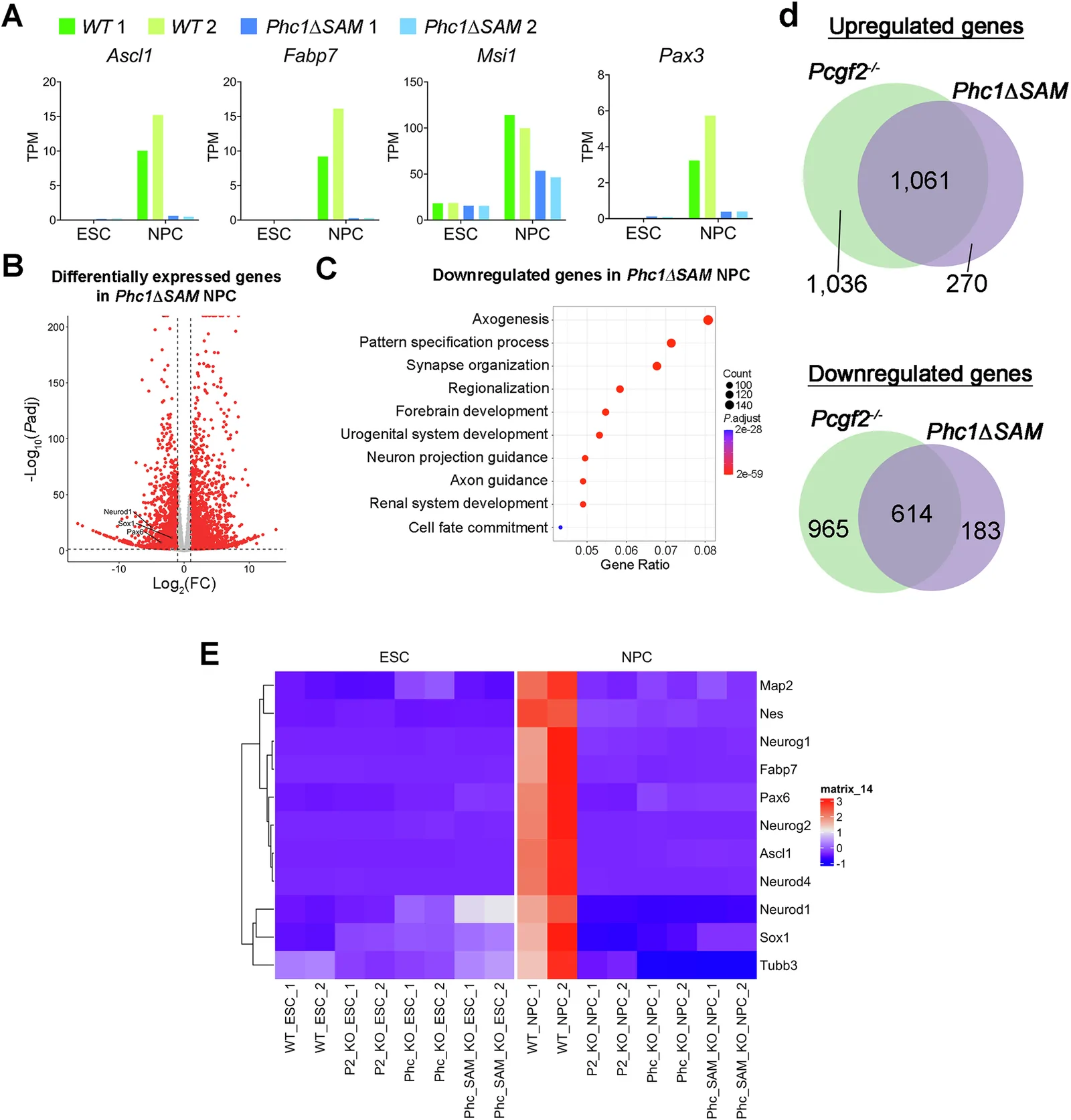
SAM domain truncation alters the NPC transcriptomic profile similar to Pcgf2 deletion. (**A**) Bar graphs show the TPM values for NPC and neuron-related gene markers in WT and Phc1∆SAM ESCs and NPCs. (**B**) Volcano plot of differentially expressed genes (DEGs) in Phc1∆SAM NPCs compared to WT NPCs. *p* values are calculated by Wald test using DEseq2 package. Red dots denote significant DEGs, and gray dots denote insignificant DEGs. NPC markers such as NeuroD1, Sox1, and Pax6 are indicated among the downregulated genes. (**C**) GO analysis of downregulated genes in Phc1∆SAM NPCs. Gene ratio (x-axis) indicates the proportion of genes in each GO term. *p* values are calculated by the hypergeometric distribution embedded in the clusterprofiler package. (**D**) Venn diagrams show the number of differentially expressed genes that are either exclusive or shared between Pcgf2^−/−^ and Phc1∆SAM NPCs based on RNA-seq data. (**E**) Heatmap using RNA-seq data showing selected neuronal genes targeted by cPRC1.2 loops.

To examine the impact of *Phc1∆SAM* on neuronal gene activation, we differentiated the *Phc1∆SAM* and *WT* ESCs into NPCs. Through RT-qPCR analysis, we found that disrupting the Phc1 SAM domain caused the failure of induction of NPC markers (*Pax6* and *Nes*) (Fig. 4C). Additionally, from our transcriptomic analysis, we found that the expression of several neuronal markers, such as *Ascl1*, *Fabp7*, *Msi1*, and *Pax3*, are downregulated in *Phc1∆SAM* NPC compared to *WT* NPC (Fig. EV3A). These results suggest a similar NPC differentiation defect as seen in *Pcgf2*^*−/−*^ cells (Figs. 1 and EV1). Further transcriptomic analysis showed that many genes related to neuronal cell fate transition were downregulated in *Phc1∆SAM* NPCs compared with *WT* (Fig. EV3B,C). To further assess the transcriptomic changes caused by Pcgf2 and Phc1 disruption, we performed a Principal Component analysis (PCA) (Fig. 4D). We also created a Phc1 total knockout (*Phc1*^*−/−*^) ESC cell line for the comparison (Appendix Fig. S4D,E). As shown in Fig. 4D, all ESC samples, including *WT*, *Pcgf2*^*−/−*^, *Phc1*^*−/−*^, and *Phc1∆SAM*, are clustered closely, indicating a minor effect on gene expression caused by cPRC1.2 disruption. However, NPC samples are scattered more distantly, with *WT* and *Pcgf2*^*−/−*^ being the furthest apart from each other, and *Phc1*^*−/−*^ and *Phc1∆SAM* are placed relatively closer to *Pcgf2*^*−/−*^ compared to *WT* (Fig. 4D). This may reflect the more dramatic impact of Pcgf2 deletion on both cPRC1 and ncPRC1 than Phc1 disruption, with limited effect on cPRC1. Our early analysis in *Pcgf2*^*−/−*^ NPCs revealed a specific failure to induce neuro-ectodermal genes targeted by the cPRC1.2 loops (Fig. 3D). When we examined the impact of *Phc1∆SAM* on PRC1.2 loop-targeted genes, we found that SAM deletion led to a similar observation, with a reduction in cPRC1.2 loop-targeted gene expression related to neuro-ectoderm but not the ones unrelated to neuro-ectoderm roles (Fig. 4E). We compared the differentially expressed genes between *Pcgf2*^*−/−*^ and *Phc1∆SAM* NPCs and found that 79.7% (1061 out of 1331) of upregulated genes and 77.0% (614 out of 797) of downregulated genes in *Phc1∆SAM* are shared by *Pcgf2*^*−/−*^ (Fig. EV3D). In addition, many neuronal genes targeted by cPRC1.2 loops failed to be induced upon differentiation in *Pcgf2*^*−/−*^, *Phc1*^*−/−*^, and *Phc1∆SAM* NPCs (Fig. EV3E). In summary, by disrupting cPRC1 oligomerization through SAM domain deletion of Phc1, our results show a similar trend to Pcgf2 deletion in affecting cPRC1.2 target gene expression, further supporting the role of cPRC1.2-mediated loops in activating genes critical for neuronal lineage differentiation.

### CTCF is colocalized with cPRC1.2

**Figure 5:**
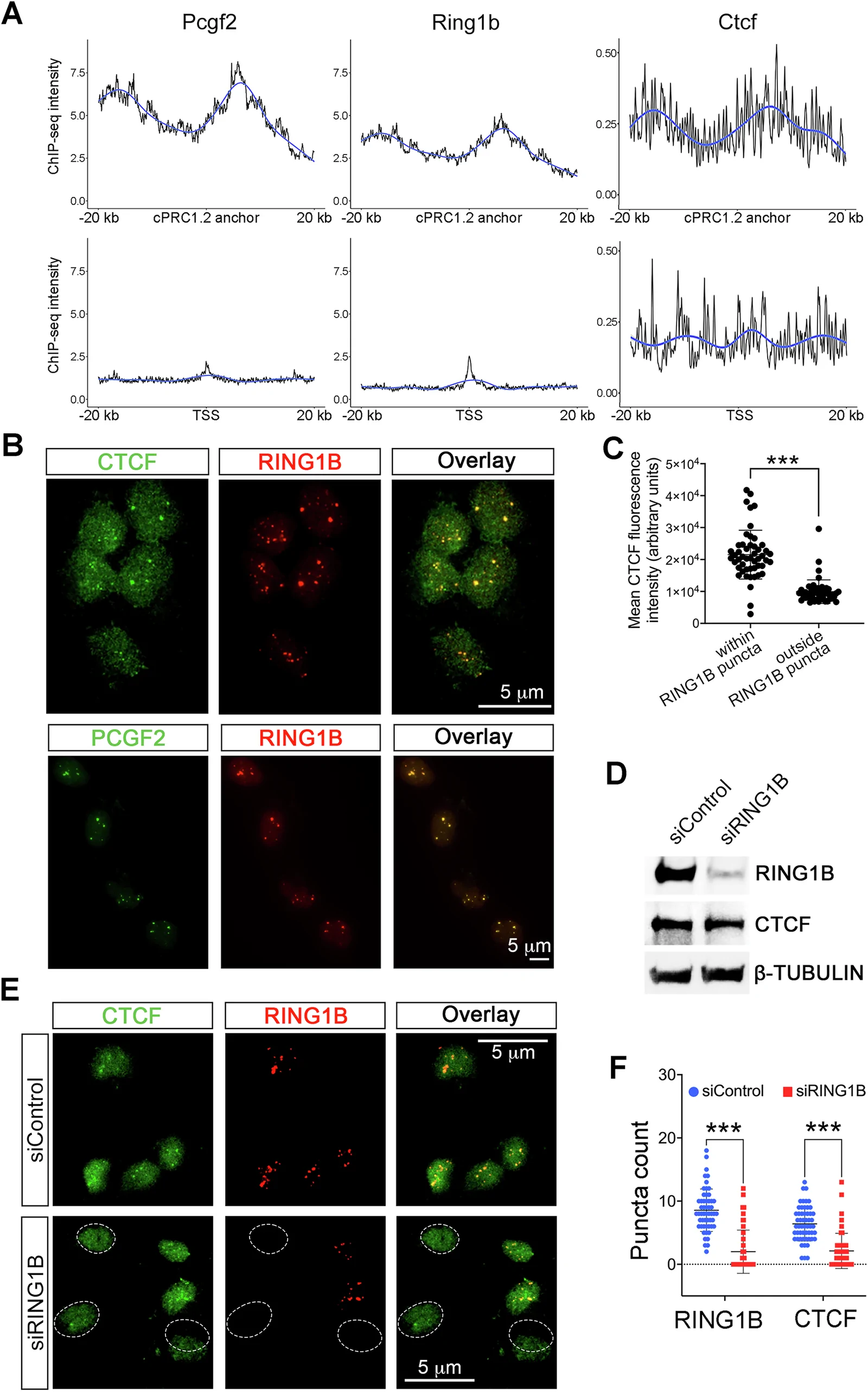
CTCF is colocalized at cPRC1.2 sites. (**A**) ChIP-seq enrichment of Ring1b, Pcgf2, and Ctcf at either genes localized by cPRC1.2 loop anchors (top panel) or at expression-matched genes void of cPRC1.2 loop anchors (bottom panel) in ESCs. (**B**) Immunofluorescent images of U2OS cells co-stained for CTCF and RING1B (top panel) and PCGF2 and RING1B (bottom panel). (**C**) Quantification of CTCF fluorescence within or outside RING1B puncta (*n* = 50). The center line represents the mean, and the ends represent the error bars from the standard deviation. A *t*-test was performed with a *p* value of 6.8e-16. ***p < 0.001. (**D**) Immunoblotting of RING1B and CTCF in RING1B knockdown (siRING1B) and control (siControl) U2OS cells. (**E**) Immunofluorescent images of control (siControl) and RING1B knockdown (siRING1B) U2OS cells showing a reduction in CTCF puncta with RING1B knockdown. White circular outlines indicate the nuclei boundaries for cells with successful knockdown. (**F**) Dot plots showing the number of RING1B and CTCF puncta in siControl (*n* = 54) and siRING1B (*n* = 50) U2OS cells. The center line represents the mean, and the ends represent the error bars from the standard deviation. From left to right, *p* values are 1.6e-16 and 3.2e-12, calculated by Student’s *t*-test. ***p < 0.001. Source data are available online for this figure.

Our Hi-C analysis identified CTCF enrichment at anchors of selected cPRC1.2 chromatin loops (Fig. 2A), raising the question of whether it cooperates with PRC1 in chromatin looping. Interestingly, a previous report has shown that CTCF binding is highly correlated with chromatin loops established by PRC2 (Xu et al, 2022), which catalyzes H3K27me3 as docking sites for cPRC1 binding (Fischle et al, 2003; Min et al, 2003). To examine the global chromatin distribution of CTCF in relation to cPRC1.2 loops, we calculated the binding intensities of PRC1.2 and CTCF at cPRC1.2 loop anchors in ESCs. As a result, both Ring1b and Pcgf2 show strong genomic enrichment at cPRC1.2 loop anchors (Fig. 5A), while, as a control, there is much less Ctcf occupancy at TSS of genes without cPRC1.2 localization and with matching expression level (Fig. 5A). Additionally, genome-wide analysis of Ctcf-bound sites revealed an enrichment of Ring1b (Appendix Fig. S5), confirming that these factors co-localize, at least at a subset of genomic regions.

**Figure EV4:**
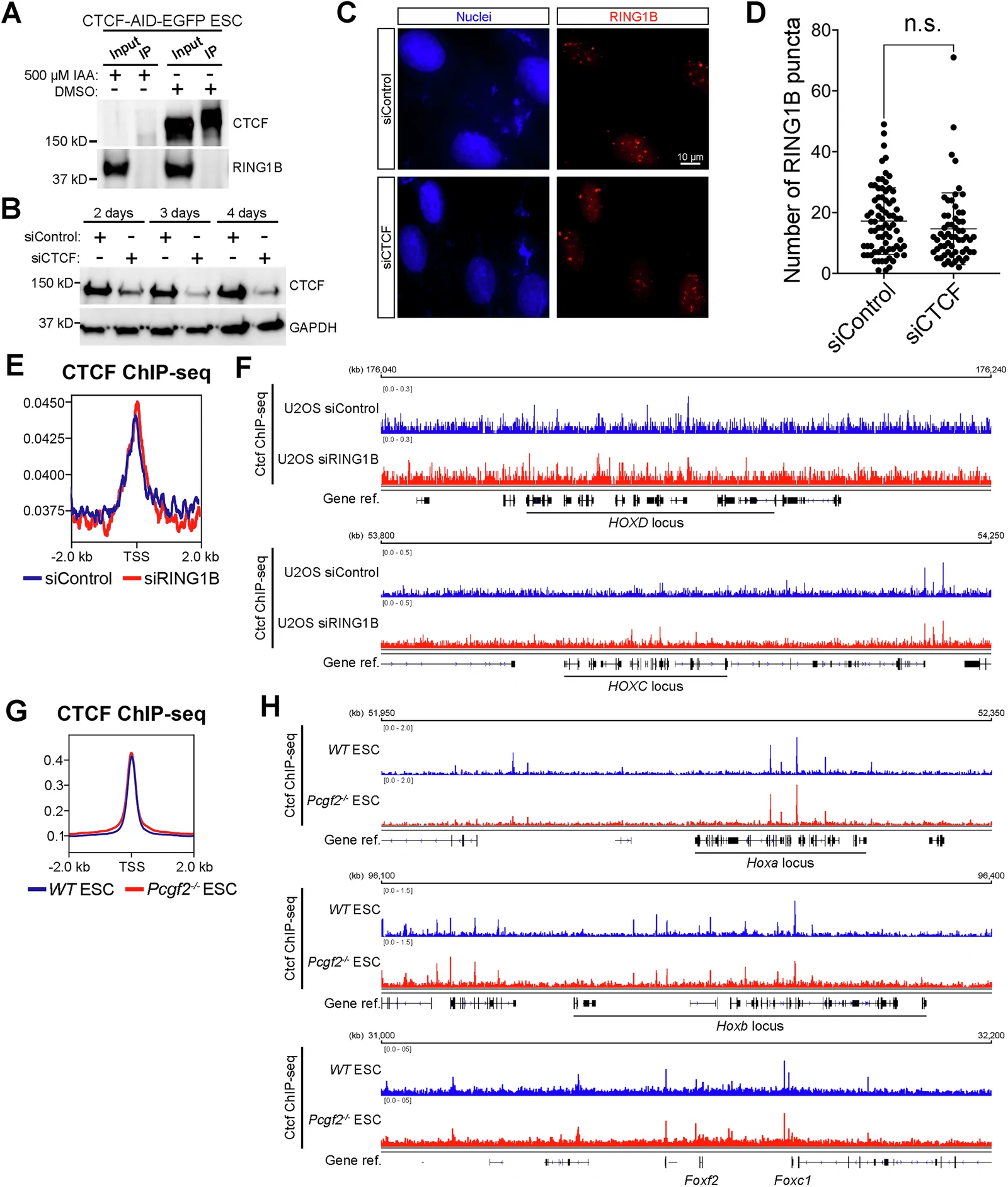
CTCF is not required for PcG body formation. (**A**) Immunoprecipitation using CTCF antibody in CTCF-AID-EGFP ESCs with 500 mM indole-3-acetic acid (IAA) or DMSO treatment for 24 h, followed by immunoblotting for CTCF and RING1B. (**B**) Immunoblotting of CTCF in U2OS cells with CTCF knockdown (siCTCF) or control (siControl) over 2, 3, and 4 days. (**C**) Immunofluorescent images of siControl and siCTCF U2OS cells stained for RING1B. (**D**) Dot plots of PcG body count in siControl (*n* = 77) and siCTCF (*n* = 60) U2OS cells. The center line represents the mean, and the ends represent the error bars from the standard deviation. A *t*-test was performed. n.s. not significant. (**E**) Line graphs show the CTCF enrichment based on ChIP-seq output in siControl and siRING1B U2OS cells. (**F**) IGV graphs show the ChIP-seq tracks of CTCF enrichment in siControl and siRING1B U2OS cells at the *HOXD* (top panel) and *HOXC* (bottom panel) loci. (**G**) Line graphs show the CTCF enrichment based on ChIP-seq output in WT and Pcgf2^−/−^ ESCs and NPCs. (**H**) IGV graphs show the ChIP-seq tracks of Ctcf enrichment in WT and Pcgf2^−/−^ ESCs at the *Hoxa* (top panel), *Hoxb* (middle panel), and *Foxq1/Foxc1* (bottom panel) loci.

Direct physical interactions between PRC1 and CTCF have not been detected through affinity purification studies in the past (Boyer et al, 2006; Gao et al, 2012; Gearhart et al, 2006; Saurin et al, 2001; Shao et al, 1999; Trojer et al, 2011; Vidal, 2009; Wang et al, 2004). We did not observe their interaction by immunoprecipitation either (Fig. EV4A). cPRC1 components are known to be concentrated in PcG bodies, nuclear structures that serve as hubs to tether distantly located PRC1-targeted loci to maintain their silencing (Bantignies et al, 2011; Lanzuolo et al, 2007). Interestingly, CTCF has been found to be located at PcG bodies, but the functional importance remained unclear (MacPherson et al, 2009). Therefore, to understand the relationship of CTCF with PRC1-associated PcG bodies, we performed IF studies in U2OS cells, which have been used previously for their ease of visualizing PcG bodies (Alkema et al, 1997; Gunster et al, 1997; Hernandez-Munoz et al, 2005; Voncken et al, 1999). Albeit present throughout nuclei, strong CTCF punctate signals were detected within RING1B-positive PcG bodies (Fig. 5B, top panel). PCGF2 also showed a high level of colocalization with RING1B (Fig. 5B, bottom panel), indicating the presence of the cPRC1.2 complex at PcG bodies. With ImageJ, we quantified the CTCF fluorescent intensity within or outside PcG bodies and found that CTCF was highly concentrated at RING1B-enriched PcG bodies (Fig. 5C).

CTCF may play a causative role in PcG body formation, or, inversely, its localization at PcG bodies depends on PRC1. To test these possibilities, we performed siRNA-mediated knockdown analyses. In U2OS cells treated with siRNA for RING1B, we achieved almost complete silencing of RING1B compared with cells treated with control siRNAs, demonstrated by immunoblotting analysis (Fig. 5D). The loss of RING1B expression in U2OS cells led to the disruption of PcG bodies shown by the lack of fluorescent RING1B and an accompanied disappearance of CTCF puncta, compared to control (Fig. 5E). With ImageJ quantification, we observed a reduction in nuclear puncta positive for both RING1B and CTCF upon RING1B knockdown (Fig. 5F). On the other hand, when we knocked down CTCF (Fig. EV4B), we did not see a dramatic change in RING1B-positive foci (Fig. EV4C,D), suggesting CTCF is not required for PcG body formation. To further investigate if the genomic localization of CTCF is altered by PRC1 disruption, we performed ChIP-seq analysis in U2OS cells transfected with either siControl or siRING1B. We found that there is no dramatic change in the CTCF localization globally and at select cPRC1.2 loop target sites upon RING1B depletion (Fig. EV4E,F). In parallel, in *Pcgf2*^*−/−*^ ESCs, no change in Ctcf chromatin localization were observed, compared to *WT* (Fig. EV4G,H). Altogether, these results suggest a potential collaboration between cPRC1.2 and CTCF in proximity for regulating chromatin loops and gene activity.

### Disruption of cPRC1.2 inhibits the formation of active loops mediated by CTCF upon neuronal differentiation

**Figure 6:**
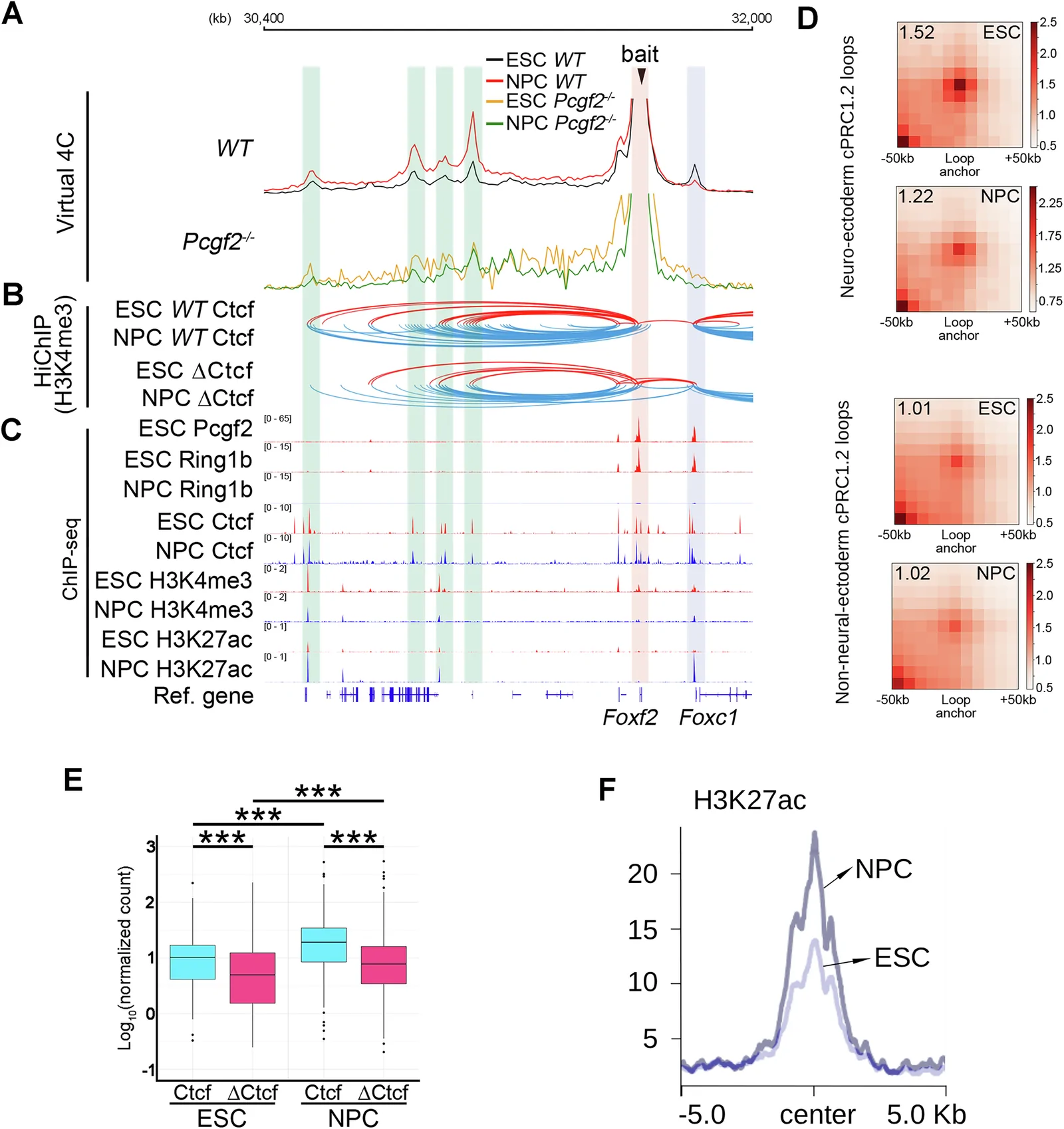
cPRC1 loops prime subsequent active CTCF loops during differentiation. (**A**) Virtual 4C analysis of *Foxf2/Foxc1* locus using Hi-C data in *WT* and *Pcgf2*^*−/−*^ ESCs and NPCs. The bait was set at the loop anchor at *Foxf2* (highlighted in red). Note the increased interactions in NPCs outside of the loop domain (highlighted in green) and the decreased interaction between *Foxf2* and *Foxc1* (highlighted in purple) in the *WT* cells. Interactions are normalized based on the peak height of the bait. (**B**) HiChIP analysis for H3K4me3, from a previous study (Kubo et al, 2021), shows the increase of promoter-enhancer or promoter-promoter loops in *WT* NPCs compared with ESCs (top panel). These loops are weakened when Ctcf is deleted (bottom panel). These loops are well correlated with the Virtual 4C peaks. (**C**) ChIP-seq tracks showing the enrichment for Pcgf2, Ring1B, Ctcf, H3K4me3, and H3K27ac in *WT* ESCs and NPCs. (**D**) APA plots for cPRC1.2 loops targeting neuro-ectoderm genes (top) or non-neuro-endoderm genes (bottom) in *WT* ESCs and NPCs at 10-kb resolution. A total of 73 neuro-ectoderm and 76 non-neuro-ectoderm cPRC1.2 loops were analyzed. Normalized pileup values are displayed at the top-left. (**E**) Box plots showing the contact frequency of active loops sharing anchors with cPRC1.2 loops in control (WT Ctcf) and Ctcf-depleted (∆Ctcf) ESCs and NPCs. Total number of analyzed loops: *n* = 1547. Boxes show the median (midline) and interquartile range, and whiskers extend to the minimum or maximum data point, or to 1.5 × interquartile range beyond the box, whichever is shorter. From left to right, *p* values are 0.00026, 2.6e-11, 7.5e-5, and 8.5e-7, calculated by Student’s *t*-test. ***p < 0.001. (**F**) ChIP-seq enrichment of H3K27ac at active loops sharing anchors with cPRC1.2 loops in ESCs and NPCs.

Given our observation of the localization of CTCF in cPRC1.2 loop anchors as well as in PcG bodies (Fig. 5), we hypothesized that CTCF may coordinate with cPRC1.2 to regulate the lineage-specific gene activation through chromatin looping. To investigate this, we performed virtual 4C analysis on Hi-C data from *WT* and *Pcgf2*^*−/−*^ ESCs and NPCs (Bonev et al, 2017). We first chose one loop anchor as the bait and then calculated its contact frequencies with its neighboring regions. At the *Foxf2/Foxc1* locus, we observed that the interaction strength of the previously identified cPRC1.2 loop was notably weakened at the NPC stage (Fig. 6A, top panel, purple-shaded peaks). Globally, cPRC1.2 loops that target the neuro-ectodermal genes often show a decreased loop strength upon differentiation into NPCs (Fig. 6D). In contrast, cPRC1.2 loops targeting non-neuro-ectodermal genes show no significant change (Fig. 6D). Interestingly, accompanying the weakening of cPRC1.2 loops at the *Foxf2/Foxc1* locus upon differentiation, the bait showed increased contact with adjacent genomic regions bound by CTCF but outside the cPRC1.2 loop (Fig. 6A,C, green-shaded areas), indicating the enhancement of CTCF-mediated chromatin loops. Furthermore, Pcgf2 deletion weakened these newly formed loops in *Pcgf2*^*−/−*^ NPCs (Fig. 6A, bottom), which suggests that cPRC1.2 is required for the formation of subsequent CTCF loops.

A previous study, through HiChIP analysis, using an H3K4me3 antibody, identified promoter-enhancer and promoter-promoter loops gained during NPC differentiation (Kubo et al, 2021). Furthermore, the formation of these loops requires the presence of CTCF and promotes gene activation (Kubo et al, 2021). To test whether the newly gained loops we observed in NPCs were previously identified CTCF-mediated active loops, we took advantage of the published HiChIP dataset and extracted the H3K4me3 loops surrounding our identified cPRC1.2 loop sites. As a result, we found an increase of H3K4me3 HiChIP contacts between the bait and the genomic regions showing enhanced virtual 4C contacts (Fig. 6B, green-shaded areas). In addition, these H3K4me3 loops are dependent on CTCF since auxin-induced CTCF deletion (∆Ctcf) led to reduced H3K4me3 HiChIP contacts (Fig. 6B, compare NPC control and NPC ∆Ctcf). To better understand the roles of Ctcf at cPRC1.2 loop sites, we utilized the H3K4me3 HiChIP data in *WT* and ∆Ctcf ESC and NPC and assessed the Ctcf-dependent active loop strength at those sites. We observed a significant reduction of active loop contacts at cPRC1.2 loop anchors in the ∆Ctcf ESCs and NPCs compared to *WT* cells (Fig. 6E). This indicates that the presence of cPRC1.2 loops are essential for the subsequent formation of Ctcf-dependent active loops upon differentiation.

It has been suggested that the distal promoter may act as an enhancer to stimulate the gene activity downstream of the proximal promoter (Diao et al, 2017; Li et al, 2012). Therefore, we used the H3K4me3/H3K27ac ratio to gauge the status of the distal genomic elements looped to the proximal promoter targeted by cPRC1.2 loops. As seen in Fig. 6C,F, an increase in H3K27ac occupancy and a decrease in H3K4me3 level were observed at distal looped regions upon differentiation, suggesting the enhancer activity of the distal elements. Next, we measured the expression of cPRC1.2 loop genes in WT and ∆Ctcf cells during differentiation using published RNA-seq data from the same study (Kubo et al, 2021). Across the differentiation stages, the expression of overall cPRC1.2 loop genes do not change significantly between WT Ctcf and ∆Ctcf cells (Appendix Fig. S6A, top panel). However, we do observe a significant upregulation in neuro-ectoderm cPRC1.2 loop genes between WT Ctcf and ∆Ctcf cells at day 2 of differentiation (Appendix Fig. S6A, bottom panel). While these genes are further significantly activated at day 4 from day 2 of differentiation, they failed to be further activated in ∆Ctcf cells (Appendix Fig. S6A, bottom panel). There is no significant difference in gene expression between WT Ctcf and ∆Ctcf cells at day 4 of differentiation (Appendix Fig. S6A, bottom panel). When we examined the expression of several critical neuronal markers that are also targeted by cPRC1.2 loops, such as *Neurog1*, *Pax3*, *Pax6*, and *Hes3*, we found a prominent trend of downregulation across the differentiation stages upon Ctcf depletion (Appendix Fig. S6B). Overall, our analysis indicates that Pcgf2 deletion disrupts cPRC1.2 chromatin loops, which in turn reduces the formation of Ctcf-mediated activating loops during differentiation, ultimately preventing the expression of critical neuronal genes.

### Deleting cPRC1.2 anchor disrupts Ctcf loop formation and gene activation of the targeted gene

**Figure 7:**
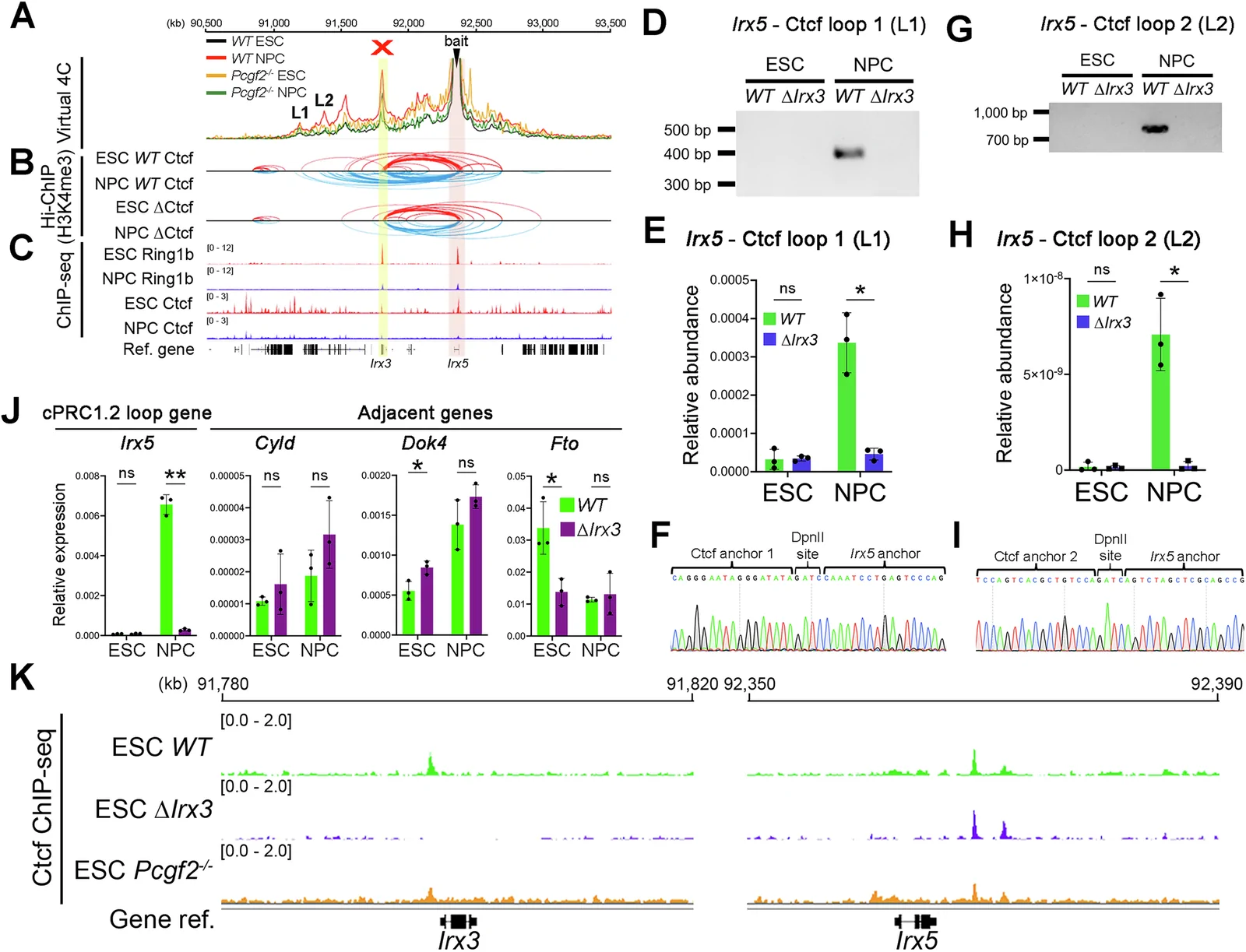
Disruption of the cPRC1.2 loop at the *Irx3/Irx5* locus leads to the loss of Ctcf loops and target gene activation. (**A**) Virtual 4C analysis of *Irx3*/*Irx5* locus using Hi-C data in *WT* and *Pcgf2*^*−/−*^ ESCs and NPCs. The bait was set at the loop anchor at *Irx5* (highlighted in red). The *Irx3* anchor deletion (*∆Irx3*) region is highlighted in yellow. Interactions are normalized based on the peak height of the bait. (**B**) HiChIP analysis for H3K4me3 at the *Irx3/Irx5* locus, using data from a previous study (Kubo et al, 2021), shows the increase of promoter-enhancer or promoter-promoter loops in *WT* NPCs compared with ESCs (top panel). These loops are weakened when Ctcf is deleted (bottom panel). (**C**) ChIP-seq tracks showing the enrichment for Ring1B and Ctcf in *WT* ESCs and NPCs. (**D**, **G**) Agarose gel shows the PCR product from 3C for *Irx5*-Ctcf loop 1 (L1) and *Irx5*-L2 interactions, respectively, in *WT* and *∆Irx3* ESCs and NPCs. (**E**, **H**) Bar graph shows the qPCR analysis of 3C for -qPCR analysis for *Irx5*-L1 and *Irx5*-L2 loops, respectively, in *WT* and *∆Irx3* ESCs and NPCs. The relative abundance is calculated by normalizing against the internal loading control for each sample. Mean and standard deviation values were derived from three biological replicates. Error bars represent the standard deviation. From left to right, *p* values are 0.89, 0.0032, 0.83, and 0.0034, calculated by Student’s *t*-test. *p < 0.05, **p < 0.01, ns not significant. (**F**, **I**) Sanger sequencing results for *Irx5*-L1 and *Irx5*-L2 fragments generated by 3C in *WT* NPCs. (**J**) Bar graphs show the RT-qPCR result for *Irx5* and three non-loop target adjacent genes (*Cyld*, *Dok4*, and *Fto*) as controls in *WT* and *∆Irx3* ESCs and NPCs. Mean and standard deviation values were derived from three biological replicates. Error bars represent the standard deviation. From left to right, *p* values are 0.60, 2.5e-5, 0.39, 0.17, 0.024, 0.15, 0.020, and 0.67, calculated by Student’s *t*-test. *p < 0.05, **p < 0.01, ns not significant. (**K**) The ChIP-seq tracks for Ctcf in WT, *∆Irx3*, and *Pcgf2*^*−/−*^ ESCs at *Irx3* (left panel) and *Irx5* (right panel) loci. Note the loss of Ctcf enrichment at the *Irx3* deletion region in *∆Irx3* ESC but unaffected at the *Irx5* site. Source data are available online for this figure.

**Figure EV5:**
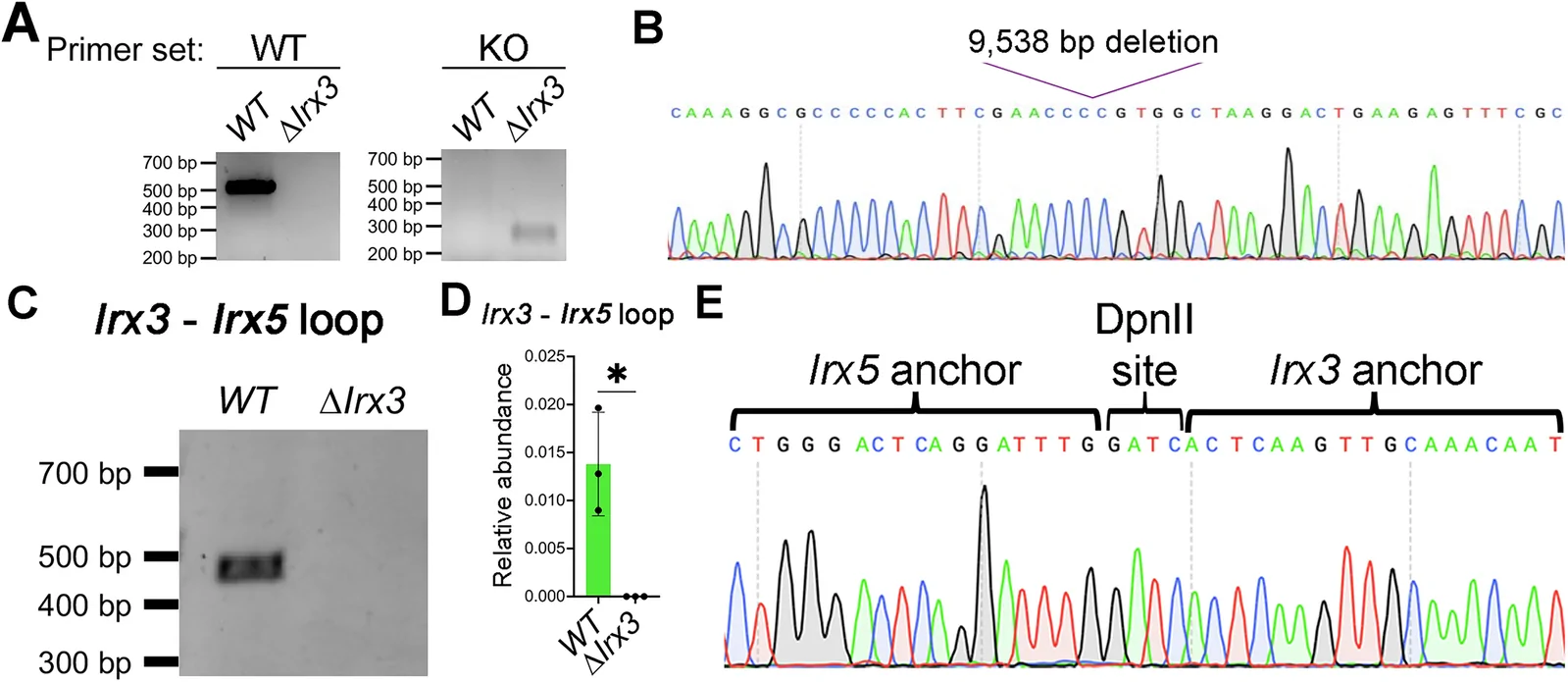
Irx3 anchor deletion disrupts Irx3-Irx5 cPRC1.2 loop formation. (**A**) Agarose gel picture shows the PCR output of Irx3 anchor deletion (∆Irx3) in ESCs using specific primer sets to amplify the intact sequence (WT) or the excised sequence (KO). (**B**) Sanger sequencing result of the homozygous deletion of the Irx3 anchor in WT ESCs. (**C**) Agarose gel picture shows the 3C-PCR result of the cPRC1.2 Irx3-Irx5 loop interaction in WT and ∆Irx3 ESCs. (**D**) The bar graph shows the 3C-qPCR analysis for Irx3-Irx5 loop interaction between WT and ∆Irx3 ESCs. The relative abundance is calculated by normalizing against the internal loading control for each sample. Each data point represents three biological replicates. Error bars represent standard deviations. A *t*-test was performed, yielding a *p* value of 0.05. *p < 0.05. (**E**) Sanger sequencing result for Irx3-Irx5 loop interaction fragment in WT ESCs derived from 3C.

**Figure 8:**
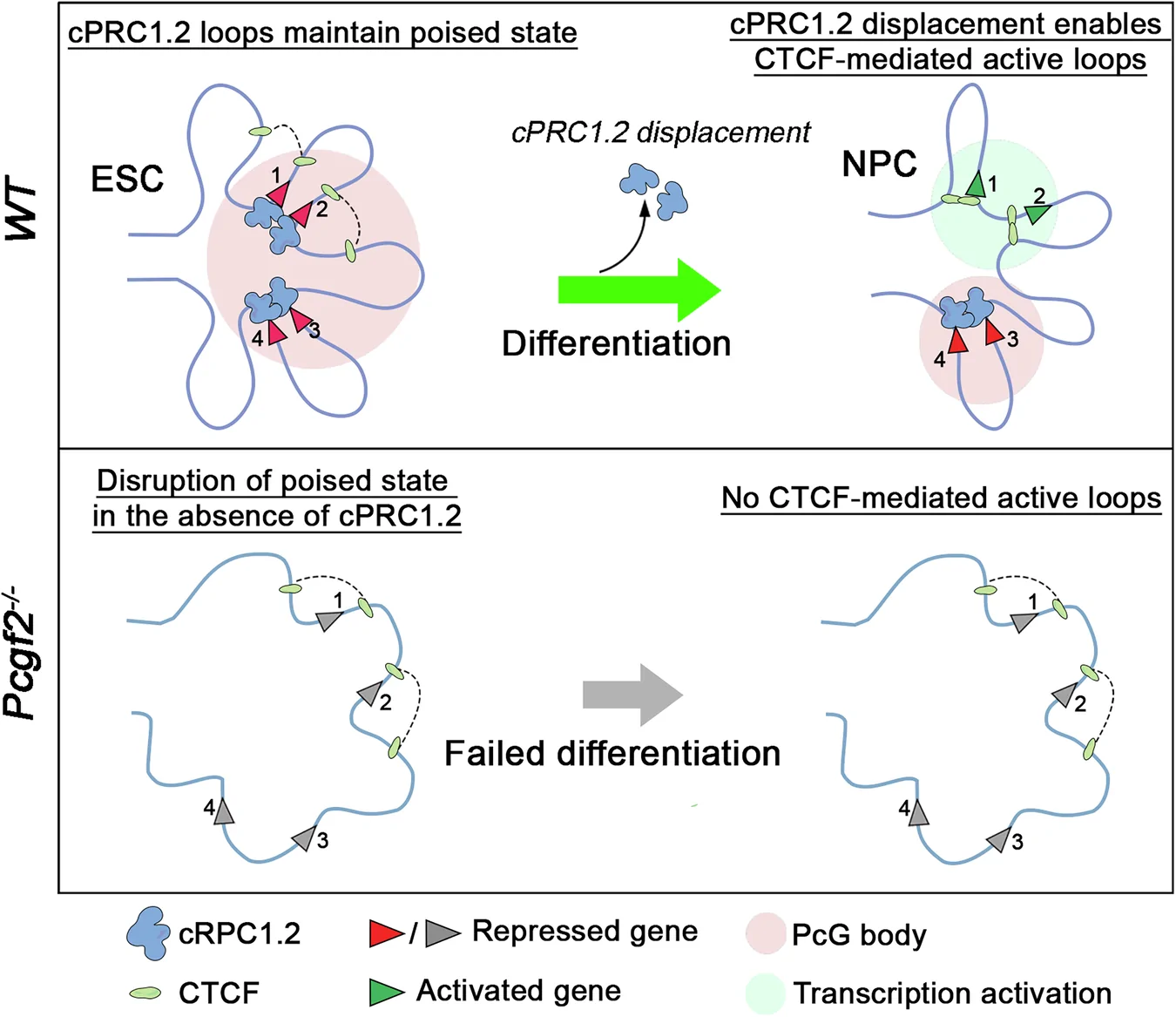
Proposed model for chromatin loops regulating neuronal differentiation. Schematic model illustrating the role of cPRC1.2 loops in regulating lineage-specific gene expression through establishing a primed state for subsequent CTCF-mediated loops during neuronal differentiation.

We have shown that Pcgf2 deletion in ESC resulted in the loss of cPRC1.2 loops and subsequent loss of Ctcf-dependent active loops, and that disruption of cPRC1.2 complex through either Pcgf2 or Phc1 SAM deletion leads to dysregulation of loop target genes. It remains possible, however, that these effects are not solely due to looping but may also involve compromised PRC1 spreading or other functions. Through virtual 4C and H3K4me3 HiChIP analysis, we observed an increase of contact frequency in NPC corresponding to active loops anchored at *Irx5* and distal Ctcf-occupied sites, which was lost upon Pcgf2 deletion (Fig. 7A–C). To directly test the requirement of the cPRC1.2 loop at the *Irx3/Irx5* locus for downstream effects, we engineered an ESC line by deleting the *Irx3* anchor (*∆Irx3*) of the *Irx3*/*Irx5* loop. A ~9.5 kB DNA fragment was deleted and confirmed with PCR and Sanger sequencing (Fig. EV5A,B). We next performed 3C to examine the chromatin loops at the *Irx3/Irx5* locus. First, we confirmed the loss of the *Irx3* to *Irx5* loop mediated by cPRC1.2 (Fig. EV5C–E). We next measured the contact enrichment between the intact *Irx5* anchor and the distal regions bound by Ctcf, for which we chose two regions, L1 and L2 (Fig. 7A). 3C-PCR and -qPCR analyses for the targeted fragments showed that there is an increase in *Irx5*-Ctcf fragment level in *WT* NPC compared to *WT* ESC, indicating the enhancement of the Ctcf-mediated loops during differentiation (Fig. 7D–I). More importantly, the probed contact fragments are significantly depleted in *∆Irx3* NPC, compared to *WT* NPC (Fig. 7D–I). Furthermore, we quantified the gene expression of *Irx5* with RT-qPCR and found that *Irx5* failed to be induced in *∆Irx3* NPC compared to *WT* NPC (Fig. 7J). In contrast, the control genes flanking the loop region, *Cyld*, *Dok4*, and *Fto*, did not show a significant expression change (Fig. 7J). To rule out the possibility that cPRC1.2 disruption has any effect on Ctcf genomic distribution, we conducted ChIP-seq analysis in *WT*, *∆Irx3, and Pcgf2*^*−/−*^ ESCs. As a result, deletion of the *Irx3* anchor has no effect on Ctcf binding at the *Irx5* anchor (Fig. 7K). These findings provide direct evidence that the cPRC1.2 loop itself is essential for the downstream formation of Ctcf-mediated loops and target gene activation during neuronal differentiation. Taken together, our results suggest a mechanism by which PRC1 and CTCF orchestrate chromatin looping interactions, which contributes to bivalent gene activation critical for neuronal differentiation (Fig. 8).

## Discussion

Our study supports a novel mechanism through which PRC1, traditionally linked to gene repression, plays an unexpected role in activating essential developmental genes by collaborating with CTCF to reorganize chromatin topology. The cPRC1.2 complex forms poised chromatin loops at bivalent promoters of critical lineage-specific TFs in ESCs, and upon neuronal differentiation, cPRC1.2 loops dissolve with simultaneous enhancement of pre-formed CTCF-mediated active loops (Fig. 8, top panel). This rearrangement of chromatin structure allows for the timely activation of key TFs for the transition to NPCs. The loss of cPRC1.2 leads to the disruption of PRC1 loops in ESCs, which compromises the formation of subsequent CTCF loops and ultimately prevents neuronal TF induction (Fig. 8, bottom panel). It is worth mentioning that the loss of Pcgf2 itself may lead to halted or diverged differentiation trajectory as evidenced by the two large sets of genes that are either upregulated or downregulated in *Pcgf2*^*−/−*^ NPCs (Fig. 1A). Furthermore, as our analysis was limited to a single loop, we cannot exclude the possibility that CTCF-mediated loop formation arises indirectly from cPRC1.2 loops, occurring as a result of gene activation upon differentiation. Hence, future research incorporating genome-wide approaches analyzing multiple differentiation stages will be key to delineate the precise sequence of molecular events following the disruption of PRC1 loops in regulating cell fate transitions.

PRC1 complexes perform gene regulatory roles by maintaining a repressive chromatin environment (Jaenisch and Young, 2008; Margueron and Reinberg, 2011; Morey and Helin, 2010; Simon and Kingston, 2013). Recent evidence suggests they can also function as transcriptional activators (Frangini et al, 2013; Gao et al, 2014; Liu et al, 2021; Mousavi et al, 2012; Russo et al, 2018; Yu et al, 2012). Despite the previous studies establishing the critical role of PRC1-mediated chromatin loops in transcription, whether they positively or negatively regulate gene activity remains controversial. Although earlier studies mostly align with a repressive role of PRC1 loops (Ogiyama et al, 2018; Schoenfelder et al, 2015), it has been later observed that PRC1-mediated promoter-enhancer loops are formed in both *Drosophila* eye-antennal imaginal disks and mouse brains, resulting in target gene activation (Kondo et al, 2014; Loubiere et al, 2020). Our proposed model thus provides a means to reconcile these opposing observations. At the ESC stage, PRC1 loops target bivalent promoters to maintain their silencing while prime them for activation by subsequent topological rearrangement. Upon differentiation, PRC1 loops are weakened or disassembled to allow their targeted promoters to form CTCF-mediated loops with distal regulatory elements, leading to gene activation. This model is indeed consistent with the previous observation at the *Meis2* locus in the developing mouse brains, where a temporal transition of PRC1-mediated loop occurs between its promoter and a site within the 3’-region to a promoter-enhancer loop (Kondo et al, 2014). Interestingly, this study identified an intermediate tripartite loop formed by these elements (Kondo et al, 2014), although the molecular mechanism was unclear. We suspect that the colocalization of CTCF in PcG bodies may provide a looping mechanism to link the *Meis2* promoter to the corresponding enhancer. It will be interesting to test whether this is the case and extend further studies to elucidate how distinct types of loops dynamically regulate chromatin reorganization to activate key lineage-specific factors during development.

PcG bodies have long been observed in cells from both *Drosophila* and mammalian origins (Alkema et al, 1997; Bantignies et al, 2011; Buchenau et al, 1998; Gunster et al, 1997; Hernandez-Munoz et al, 2005; Lanzuolo et al, 2007; Messmer et al, 1992; Schoorlemmer et al, 1997; Voncken et al, 1999). Recently, chromosomal phase separation has been proposed to interpret the formation of nuclear condensates, including various nuclear bodies such as PcG bodies, heterochromatin, and transcriptional condensates (Cho et al, 2018; Feric et al, 2016; Lafontaine et al, 2021; Park et al, 2024; Stanek and Fox, 2017; Wang and Liu, 2019). Such nuclear condensates may act as structural hubs for either active gene transcription or repression. In particular, PcG bodies have been shown to organize looped PRC-associated genes for their silencing (Bantignies et al, 2011; Lanzuolo et al, 2007). Our findings of the colocalization of cPRC1.2 and CTCF in PcG bodies (Fig. 5) suggest that PcG bodies may also serve as a platform for priming genes for activation upon differentiation. However, the cPRC1.2 and CTCF relationship was primarily examined in U2OS cells and ESCs; a key remaining question is how this interaction is regulated during neuronal differentiation. Future studies integrating biochemical and genetic approaches will be needed to address how cPRC1.2 and CTCF may cooperate to achieve sophisticated gene regulation. Regarding the collaboration between CTCF and PRC1, another critical question remains: how is CTCF recruited to PcG bodies? We have shown a clear dependence of CTCF on the RING1B to be enriched in such nuclear bodies, but it is unlikely through direct interaction between CTCF and cPRC1.2. As previously reported, PRC2 is also localized to PcG bodies, so the reported interaction of PRC2 subunits with CTCF (Li et al, 2008; Wang et al, 2020) may assist the CTCF recruitment. Alternatively, an indirect mechanism may enhance CTCF retention on chromatin in the compacted PcG bodies.

Given the cell-type-dependent complexity of the PRC1 composition (Gao et al, 2012; Kloet et al, 2016), future efforts will be needed to clarify whether the different cell or animal models used in previous studies may account for the differential regulation by cPRC1 loops on distinct genes. For example, given their nearly identical subunit makeups, does cPRC1.4 have a similar effect on loops as cPRC1.2 (Gao et al, 2012)? Although our data suggest a predominant role for cPRC1.2 in regulating neuronal genes in vitro, we cannot rule out the possibility that cPRC1.4 or cPRC1.2 may be necessary for other lineages or even different neuronal cell types. Recently, it has been reported that patients harbor a missense mutation in the *Pcgf2* locus, resulting in a developmental syndrome called the Turnpenny-Fry syndrome. The developmental defects included brain tissue malformations accompanied by a varying degree of intellectual disability (Patricio Rodrigues et al, 2024; Qi et al, 2022; Turnpenny et al, 2018). These reports provide an important rationale for studying cPRC1 functions in regulating chromatin architecture in the context of development in other lineages besides neuronal lineages.

## Methods

**Table:** Reagents and tools table

Reagent/resource	Reference or source	Identifier or catalog number
**Experimental models**		
*Pcgf2*^*−/−*^ ESC	Generated in this study	
*Phc1*^*−/−*^ ESC	Generated in this study	
*Phc1∆SAM* ESC	Generated in this study	
∆*Irx3* loop anchor ESC	Generated in this study	
**Recombinant DNA**		
**Antibodies**		
Ring1b	Bethyl Laboratories	A302-869A
Pcgf2 (Mel18)	Gift from Diego Passini	N/A
Pcgf2 (Mel18)	Generated in this study	N/A
Rybp	Santa Cruz Biotechnology	SC374235
Phc1	Cell Signaling Technology	13768
Pcgf4 (Bmi1)	Bethyl Laboratories	A301-694A
Gapdh	Invitrogen	MA5-15738
β-tubulin	Abcam	ab6046
Nanog	Invitrogen	PA1-41577
H2AK119ub1	Cell Signaling Technology	8240
Histone H3	Bethyl Laboratories	A300-823A
CTCF	Active Motif	91285
CTCF	BD Biosciences	BD612148
Alexa Fluor 488	Thermo Fisher Scientific	A28175
Alexa Fluor 568	Thermo Fisher Scientific	A-11011
IgG	Cell Signaling Technology	2729S
**Oligonucleotides and other sequence-based reagents**		
**gRNAs for knock-out**		Appendix Table S4
*Pcgf2*	Guide 1: CACGGATTAAAATCACGGAG	
	Guide 2: GTGAGTTGAATTCGGGGGTG	
*Pcgf4*	Guide 1: TTATAAACCGCTCAGCATTC	
	Guide 2: TACACAGTGTTCGCCTTCT	
*Phc1*	Guide 1: ACTGCAGCGGCAACCTAATG	
	Guide 2: GGGGGAGTTACTGCAAGGTT	
*Phc1* (SAM domain)	Guide 1: TCCTAGCCAATGGAGCGTCG	
	Guide 2: AGTGCCATGAACATCAAATT	
*Irx3* loop anchor	Guide 1: ATCCAAGGCGTCACTACTCT	
	Guide 2: ACCTTCTAGCATTCTGCCCG	
**Genotyping PCR primers for knock-out**		Appendix Table S4
*Pcgf2*	Forward: GGTGACACTTCCCAAAT	
	Reverse: GAGCCAGAAGCTCACTCCTGG	
*Pcgf4*	Forward: ATTCTGATCTCATTAGTAAATTCCTTT	
	Reverse: CATAACTGTGAGGTTTACTTTCTTTT	
*Phc1*	Forward: GAGTAGACTACTGAATCCAACATTG	
	Reverse: CTGCAACCTTAATCTAAGAACCAC	
*Phc1* (SAM domain)	Forward (Common): GCCGGGGATCAGATAATTCCA	
	Reverse (WT): AATGTGGACCTGGTTCTGCT	
	Reverse (KO): GTTCTGCCTCCAAACCCCAC	
*Irx3* loop anchor	Forward (Common): GCTAGTGGTCTCCATTCTGAGG	
	Reverse (WT): TTCGTCCTGTAGAGGGAGCTC	
	Reverse (KO): GGCAAACTGGAAAGTAGCGG	
**Sanger sequencing primers for knock-out**		Appendix Table S4
*Pcgf2*	CCAGGAGTGAGCTTCTGGCTC	
*Pcgf4*	ATTCTGATCTCATTAGTAAATTCCTTT	
*Phc1* (SAM domain)	CCATCCACGCCAGAGTTACA	
*Irx3* loop anchor	ATGTGGGCACCGGTCTTCAT	
**RT-qPCR primers**		Appendix Table S4
Mouse *Gapdh*	Forward: GCGGCACGTCAGATCCA	
	Reverse: CATGGCCTTCCGTGTTCCT	
Mouse *Nanog*	Forward: AGGCTTTGGAGACAGTGAGGTG	
	Reverse: TGGGTAAGGGTGTTCAAGCACT	
Mouse *Oct4*	Forward: AGATCACTCACATCGCCAATCA	
	Reverse: CGCCGGTTACAGAACCATACTC	
Mouse *Nes*	Forward: AGTGCCCAGTTCTAGTGGTGTCC	
	Reverse: CCTCTAAAATAGAGTGGTGAGGGTTG	
Mouse *NeuroD1*	Forward: CGAGTCATGAGTGCCCAGCTTA	
	Reverse: CCGGGAATAGTGAAACTGACGTG	
Mouse *Pax6*	Forward: CTTGGGAAATCCGAGACAGA	
	Reverse: CTAGCCAGGTTGCGAAGAAC	
Mouse *Irx3* - *Irx5* loop	Primer 1a: AGAAGGCCTCTGCGTAATGTC	
	Primer 1b: GCGTAATGTCCACTTCCTTCCAAGC	
	Primer 2a: GCCGCAAATTACAAACGCAC	
	Primer 2b: CAAACGCACTGAAGCTTTGGGACAG	
	Primer 3a: GGAAATGATGCGACTCCCTCT	
	Primer 3b: GTTGCATGGAAATGATGCGACTCCCTCTG	
	Primer 4a: TTGCCTTGGTCAAAAGGTGC	
	Primer 4b: GAGTGAAAGATGCAGCCACAGA	
Mouse *Irx5* - Ctcf loop 1	Primer 1a: CACCGAGGCTCCAAGTCATA	
	Primer 1b: GTCATAAATCAAAGGGATGGAGCAGAGC	
	Primer 2a: TGCCTTGGTCAAAAGGTGCAA	
	Primer 2b: GTGGAGTGAAAGATGCAGCCACAGACAGTT	
Mouse *Irx5* - Ctcf loop 2	Primer 1a: TACCCTAACCCAAACTCCCTG	
	Primer 1b: ACTCCCTGCTTTTACAGACTGG	
	Primer 2a: CTCACTAGCCAGCAAGCAGTT	
	Primer 2b: GCAAGCAGTTTGCTCGGTTG	
**Chemicals, enzymes and other reagents**		
LIF	Cayman Chemical	32066
PD0325901	Cayman Chemical	13034
Chir99021	Cayman Chemical	13122
retinoic acid (RA)	Sigma	R2625
siCTCF	Dharmacon	L-020165-00-0005
siControl	Dharmacon	D-001810-10-05
siRNF2	QIAGEN	S100095543
Lipofectamine	Invitrogen	11668019
TRIzol	Invitrogen	15596026
phenol: chloroform: isoamylalcohol	Sigma-Aldrich	P3803
formaldehyde	Thermo Fisher Scientific	28908
Proteinase K	Thermo Fisher Scientific	BP1700-100
Phusion DNA polymerase	NEB	M0530S
iTaq SYBR® Green polymerase	Bio-Rad	1725121
**Software**		
**Other**		
NovaSeq platform	Illumina	

### ESC culture, NPC, and neuronal differentiation

The ESC, NPC, and neuronal cell culture and differentiation methods were performed according to previously described protocols (Bibel et al, 2004; Hanafiah et al, 2020). About 10 cm tissue culture-treated plates were coated with 0.1% gelatin and set for at least 15 min before aspiration. The plates were then seeded with γ-irradiated SNL feeder cells (ATCC), which are derived from mouse embryonic fibroblasts (MEFs) in the pre-warmed ESC medium, which consisted of DMEM (Corning, 10-017-CM) with 15% fetal bovine serum (FBS) (R&D Systems, S10250), 1X non-essential amino acids (Gibco, 11140-050), 0.1 mM β-mercaptoethanol (Fisher BioReagents, BP176-100), 0.5X penicillin/streptomycin (Corning, 30-002-Cl), 1X sodium pyruvate (Corning, 25-000-Cl), 3.0 × 10^−3^ µg/mL LIF (Cayman Chemical, 32066), 1 μM PD0325901 (PD) (Cayman Chemical, 13034), and 3 μM Chir99021 (CH) (Cayman Chemical, 13122). The MEFs were allowed to settle and attach to the plates’ surface before plating ESCs on them. The co-cultures were incubated in an incubator set at 37 °C with 5% CO_2_ content. The ESCs were differentiated into NPCs using the hanging drop method on 10 cm cell culture plates. About 2000 cells were suspended in 25 μL differentiation medium, which consisted of DMEM with 15% FBS, 1X non-essential amino acids, 0.1 mM β-mercaptoethanol, 0.5X penicillin/streptomycin, and 1X sodium pyruvate. The droplet cultures were incubated in the incubator set at 37 °C with 5% CO_2_ content. On day 2, the formed EBs were transferred from the droplets into suspension culture plates with 10 mL differentiation medium and left on an orbital shaker at low speed in the incubator. On day 3, some of the EBs were harvested for that timepoint. The rest of the EBs were cultured in differentiation medium either with 5 μM retinoic acid (RA) (Sigma, R2625) to induce the EBs’ differentiation into NPCs or without RA to maintain their EB identity. The old medium was replaced with a new differentiation medium on the subsequent days until day 8. Some of the NPCs and EBs were harvested on day 8 for that timepoint. To further differentiate the NPCs into neurons, the NPCs were dissociated and plated at a density of 1.5 × 10^5^/cm^2^ in N2 medium (DMEM/F12 medium with 3 mg/mL glucose, 3 mg/mL Albumax, 1/100 N2 supplement, 10 ng/mL bFGF, 50 U/mL pen/strep, and 1 mM L-glutamine). On day 9, the medium was changed. On day 10, the N2 medium was switched to N2/B27 medium (50% DMEM/F12 and 50% Neural Basal, 3 mg/mL Albumax, 1/200 N2 supplement, 1/100 B27 supplement, 50 U/mL penicillin/streptomycin, and 1 mM L-glutamine). The medium was refreshed for the next consecutive 2 days before being processed for downstream assays. Cells are passaged or harvested by covering the culture plates with Trypsin (Corning, 25-052-Cl).

### U2OS culture

U2OS cells (ATCC) were cultured in growth media consisting of DMEM (Corning, 10-017-CM), 10% FBS (R&D Systems, S11550), 10% newborn calf serum (NCS) (R&D Systems, S11250), and 0.5X penicillin/streptomycin (Corning, 30-002-Cl). Cells are plated on tissue culture plates and incubated in an incubator set at 37 °C with 5% CO_2_ content. Cells are passaged or harvested by covering the culture plates with Trypsin.

### Knockdown using small interfering RNAs (siRNA)

U2OS cells were subjected to siRNA-mediated knockdown of either CTCF (siCTCF, Dharmacon, L-020165-00-0005) or RING1B (siRNF2, QIAGEN, S100095543). Each siRNA treatment was accompanied by a control (siControl, Dharmacon, D-001810-10-05). About 20 µM of each siRNA was transfected into the cells using the Lipofectamine 2000 (Invitrogen, 11668019) reagent according to the manufacturer’s recommended protocol supplemented with OPTI-MEM medium (Gibco, 31985-070). The transfected cells were incubated at 37 °C with 5% CO_2_ for 2 days before being processed for immunoprecipitation, immunoblotting, and immunofluorescence assay.

### CRISPR/Cas9-mediated gene editing

The CRISPR-Cas9-mediated gene editing in ESCs was performed according to a previously described method (Cong et al, 2013). All genetic knock-out lines were done in E14 ESCs (ATCC). Cells were cultured as described above. The guide RNAs used to knock out Pcgf2, Pcgf4, Phc1, and the truncation of Phc1 SAM domain are listed in Appendix Table S4. The guide RNAs were cloned into the CRISPR/Cas9 plasmid system, PX458 (Addgene, Plasmid #48138). The plasmids containing the guide RNAs were transfected into E14 ESCs using the Lipofectamine 2000 reagent (Invitrogen, 11668019) as described by the manufacturer, supplemented with OPTI-MEM medium (Gibco, 31985-070). The transfected cells were then sorted into single cells via flow cytometry for GFP-positive cells into 96-well plates. Live cells were sorted by flow cytometry under BSL-2 conditions using a FACSAria SORP (Becton Dickinson) instrument in Penn State College of Medicine’s Flow Cytometry core. The Pcgf2, Pcgf4, Phc1 knock-out, Phc1 SAM domain truncation, and *Irx3* loop anchor knock-out were confirmed by PCR using primers listed in Appendix Table S4. Further confirmation of the Pcgf2, Pcgf4 and *Irx3* loop anchor deletions, and Phc1 SAM domain truncation was done by Sanger sequencing (via GeneWiz) using the primers listed in Appendix Table S4. Prospective clones were also subjected to immunoblotting to confirm protein deletions and truncation.

### Immunoblotting

Whole-cell lysates were obtained from samples by using lysis buffer containing 1.5 mM MgCl_2_, 280 mM NaCl, 3 mM KCl, 0.15 mM EDTA, 15% EDTA,15 mM Tris-HCl, pH 8.0, 0.02% IGEPAL, and protease inhibitors added fresh during the experiment (0.5 mM PMSF, 1 μg/mL Pepstatin A (Sigma-Aldrich, P5318), 1 μg/mL Leupeptin (Sigma-Aldrich, EI8), 1 μg/mL Aprotinin (Sigma-Aldrich, 616370)). Protein concentration of samples was measured using the Bradford assay (Thermo Scientific, 1856209). The samples were loaded into an SDS-PAGE gel to analyze the proteins extracted. The protein samples were transferred to nitrocellulose blot using the Trans-Blot Turbo system (Bio-Rad). The blots were blocked in 5% milk and washed with 1X TBST. Refer to Appendix Table S3 for the list of antibodies used for immunoblotting, immunoprecipitation, and ChIP-seq.

### Immunoprecipitation

Immunoprecipitation methods were adapted from the previously described protocol (Gao et al, 2012). CTCF-AID-EGFP ESCs were first cultured as described in the ESC culture section above. When cells are at roughly 1.0 × 10^6^ density per sample, they were treated with 500 µM auxin in the form of indole-3-acetic acid (IAA) or DMSO at equal volume for 24 h before being collected and processed for whole-cell lysate as described in the immunoblot section above. Protein lysates were incubated for 2 h at 4 °C with 10 µg of CTCF antibody in a volume of 400 μL Buffer A (10 mM Tris-HCl, pH 8.0, 1.5 mM MgCl_2_, and 10 mM KCl) and 400 μL Buffer BN (20 mM Tris-HCl pH 8.0, 100 mM KCl, 0.2 mM EDTA, 20% glycerol, 0.5 mg/mL BSA, and 0.1% IGEPAL) supplemented with 0.5 mM PMSF, 1 μg/mL Pepstatin A, 1 μg/mL Leupeptin, and 1 μg/mL Aprotinin. About 30 μL of protein G beads were then added and incubated at 4 °C overnight. Beads were then washed with Buffer BN five times, eluted with 100 μL glycine (0.1 M, pH 2.0), and neutralized by adding 6.5 μL Tris solution (1.5 M, pH 8.8). The eluates were mixed with 1X SDS sample buffer and analyzed by SDS-PAGE, followed by immunoblotting.

### Alkaline phosphatase (AP) assay

The assay was conducted according to the manufacturer’s instructions (Stemgent, 00-0055). The culture medium was aspirated, and the cells were washed with 2 mL of 1X PBST. About 1 mL of Fix Solution was added, and the cells were incubated at room temperature (RT) for 5 min. The Fix Solution was then aspirated, and the fixed cells were washed with 2 mL of 1X PBST. The 1X PBST was aspirated, and 1.5 mL of freshly prepared AP Substrate Solution was added. The cells were incubated in the dark and wrapped with foil at room temperature for up to 15 min until the color changed. The reaction was stopped when the color turned bright to avoid non-specific staining by aspirating the AP Substrate Solution and washing the wells twice with 2 mL of 1X PBS. The cells were covered with 1X PBS or mounting medium to prevent drying, with AP expression resulting in a red or purple stain and the absence of AP expression resulting in no stain.

### MTT cell proliferation assay

The assay was conducted according to the manufacturer’s instructions (Invitrogen, 13154). The ESCs were cultured in 96-well plates. About 10 μL of the 12-mM MTT stock solution was added to each well, and a negative control was included by adding 10 μL of the MTT stock solution to 100 μL of medium alone. The wells were incubated at 37 °C for 2 h. All but 25 μL of the medium was aspirated from the wells. About 50 μL of DMSO was added to each well, then pipetted up and down thoroughly to mix. The plates were incubated at 37 °C for 10 min. Each sample was resuspended by pipetting before the absorbance was read at 540 nm. This assay was done where measurements were taken every day for 4 days.

### Immunofluorescence assay

U2OS cells were cultured on tissue culture-grade chamber slides (Thermo Fisher Scientific, 154526PK) for an immunofluorescent assay. To prepare the chamber slides, they were coated with 0.1 mg/mL poly-L-ornithine and 0.1 mg/mL laminin. The chamber slides were washed with sterile 1X PBS before U2OS cells were plated and incubated at 37 °C and 5% CO_2_. Once cells were ready to be processed, they were fixed with 4% formaldehyde for 15 min at RT. The chambers were then washed with 1X PBS. The cells were blocked and permeabilized with 5% BSA diluted in 1X PBS and 0.5% Triton X-100 solution for 1 h. The chamber slides were washed with 1X PBS before incubating the samples in a primary antibody mix (1% BSA made in 1X PBS and 0.5% Triton X-100) for 1 h at RT at the dilution recommended by their respective manufacturers. After the chamber slides were washed with 1X PBS, the samples were then incubated in a secondary fluorophore-conjugated antibody mix for 1 h at RT in the dark. The chamber slides were then washed with 1X PBS. Without leaving the samples completely dry, the chambers were removed from the slide before being stained with DAPI and mounted with a mounting solution. The samples were imaged with a fluorescence microscope. All quantifications calculated from images captured from immunofluorescent-stained samples were performed in ImageJ (Schindelin et al, 2012).

### RNA-seq sample preparation

RNA-seq experiments were performed as previously described (Gao et al, 2014). RNA was extracted using TRIzol following the manufacturer’s instructions (Invitrogen, 15596026). The RNA-seq mRNA library construction and sequencing procedures were performed by the Penn State College of Medicine Genomics Core using the TruSeq Stranded mRNA and Total RNA kit. Briefly, polyA RNA was purified from total RNA using oligo (dT) beads. The extracted mRNA fraction was initially subjected to fragmentation, reverse transcription, end repair, 3′–end adenylation, and adapter ligation. Then, the adapter-ligated strands were subjected to PCR amplification and SPRISelect (Beckman Coulter) bead purification. The unique barcode sequences were incorporated into the adapters for multiplexed high-throughput sequencing. The final product was assessed for size distribution and concentration using the BioAnalyzer High Sensitivity DNA Kit (Agilent, 5067-4626). Pooled libraries were diluted to 2 nM in EB buffer (Qiagen) and then denatured using the Illumina protocol. The denatured libraries were diluted to 10 pM by pre-chilled hybridization buffer and loaded onto a TruSeq Rapid flow cell on an Illumina Novaseq 6000 platform and run for 50 cycles using a single-read recipe according to the manufacturer’s instructions.

### RNA-seq analysis

Transcript abundances were estimated using the Kallisto quant function, with mm10 as the reference genome. The expected counts for each transcript were imported into R using the tximport package. Differentially expressed genes (DEGs) were identified using the Deseq2 package, with a fold-change larger than 2 and a *p* adjusted value smaller than 0.01 (Gu et al, 2016; Love et al, 2014). Upregulated and downregulated DEGs were used to perform Gene ontology (GO) analysis using the clusterprofiler package (Xu et al, 2024)^,^(Wu et al, 2021). RNA-seq related plots were generated using ggplot2 and complexheatmap packages (Soneson et al, 2015). Neuro-ectoderm/meso-endo PRC1 loop gene list was generated by crossing the PRC1 loop gene list with the gene list acquired from GO term neurogenesis (GO:022008), mesoderm development (GO:0007498), and endoderm development (GO:0007492) with manual curation. For RNA-seq visualization on IGV, the data processing was similar to that of ChIP-seq data.

### RT-qPCR

For the cDNA synthesis, 0.5 g of total extracted RNA was used, and the procedure was done using SuperScript III RT according to the manufacturer’s instructions (Invitrogen, 18080044). The cDNA samples were diluted 5X before being used for the qPCR step. qPCR was done according to the manufacturer’s instructions (Azura Genomics, AZ-2120). The reactions were performed and measured using the Bio-Rad CFX Connect real-time PCR detection system. Refer to Appendix Table S4 for the complete list of RT-qPCR primers used in this study.

### ChIP-seq sample preparation and sequencing

ChIP-seq samples were prepared as described previously (Gao et al, 2012). Harvested cells were cross-linked with the fix solution (1% formaldehyde, 9 mM NaCl, 0.09 mM EDTA, 0.045 mM EGTA, 9 mM HEPES buffer, pH 7.6) for 8 min. After washing and nuclei extraction, the samples were then resuspended in sonication buffer (0.5% *N*-lauroyl sarcosine, 1 mM EDTA, 0.5 mM EGTA, 10 mM Tris buffer pH 8.0, 0.5 mM PMSF, 1 µg/ml Pepstatin A, 1 µg/ml Leupeptin, and 1 µg/ml Aprotinin). The cross-linked nuclei pellets were sonicated to about 200 bp after reverse cross-linking. To start the immunoprecipitation, the protein A/G beads were first washed with TE buffer and blocked for 1 h at 4 °C with 1 mg/mL BSA before being used to pre-clear chromatin samples. Each sample was immunoprecipitated using antibodies for proteins of interest (Appendix Table S3) in 3 X ChIP buffer (3% Triton X-100, 0.3% sodium deoxycholate, 3 mM EDTA, 0.5 mM PMSF, 1 µg/ml Pepstatin A, 1 µg/ml Leupeptin, and 1 µg/ml Aprotinin). The ChIP samples were then washed with RIPA buffer (50 mM HEPES, pH 7.6, 0.5 M LiCl, 1 mM EDTA, 1% IGEPAL, 0.7% DOC, 0.5 mM PMSF, 1 µg/ml Pepstatin A, 1 µg/ml Leupeptin, and 1 µg/ml Aprotinin), and 10% of each sample was loaded on SDS-PAGE for enrichment verification by immunoblotting. The remaining 90% of the ChIP samples were subjected to DNA extraction using the ethanol precipitation method with the PCI reagent (phenol: chloroform: isoamylalcohol) (Sigma-Aldrich, P3803). The ChIP-seq library and sequencing procedures were performed by the Penn State College of Medicine Genomics Core. The ChIP-seq library was constructed using the SparQ DNA Library Prep kit. Final libraries were subsequently sequenced using the NovaSeq platform (Illumina) as described (Francis et al, 2004).

### ChIP-seq analysis

Raw FASTQ data were mapped using bwa mem against the mm10 mouse reference genome for mouse samples and hg38 for human samples. Sam files were converted to bam files, followed by sorting and indexing using samtools sort and index functions. Peak calling was performed using the MACS2 callpeak function using aligned bam files (Zhang et al, 2008). For data visualization and downstream analysis, aligned bam files were converted to bigwig files via deepTools bamCoverage command with bins per million mapped reads (BPM) normalization option (Ramirez et al, 2016). Then, ChIP-seq data were visualized in bigwig format using an integrated genome browser (IGV) or Coolbox. For the heatmap and intensity plot, the selected ChIP-seq data and correlated genomic coordinate files were processed using the computeMatrix function, and plots were generated using the plotHeatmap function in deepTools (Ramirez et al, 2016). Refer to Appendix Table S5 for the full list of publicly available ChIP-seq, Hi-C, and HiChIP datasets used in this study.

### Hi-C sample preparation and library construction

The samples were prepared using the Arima-Hi-C+ kit (Arima Genomics, A510008) based on the manufacturer’s protocol with a few modifications. Briefly, cells were harvested based on the appropriate procedure to prepare samples for Hi-C and counted using a hemocytometer. For each sample, 2 million cells were cross-linked. This was done by resuspending the cells in 1 mL of 1X PBS and cross-linked in 2% formaldehyde (Thermo Fisher Scientific, 28908) by inverting the tube ten times before incubating at RT for 10 min. Glycine was added to each sample at the 0.25 M final concentration, and the tubes were inverted ten times. The samples were then incubated at room temperature for 5 min and subsequently for 15 min on ice. The cells were centrifuged. The cell pellets were then washed with 1X PBS and centrifuged again to remove the supernatant. The cross-linked cells were stored in the −80 °C freezer before continuing with the subsequent steps. The library preparation procedures were conducted using the Arima Library Prep Module (Arima Genomics, A303011) based on the manufacturer’s instructions.

### Hi-C analysis

Raw FASTQ files were mapped against the mm10 mouse reference genome using the runHiC pipeline (https://doi.org/10.5281/zenodo.55324) with the runHiC pileup command, which compiles all processing steps into a single-line command. Briefly, the bwa aligner was used for alignments, and aligned reads were filtered for quality control and PCR duplication. Fragments were assembled and filtered to retain the fragments with at least two different restriction fragments to filter out self-ligated fragments. Last, the reads were binned at multiple resolutions to generate a contact matrix using cooler tools (Abdennur and Mirny, 2020). ICE-corrected Hi-C matrices were visualized using coolbox(Xu et al, 2021). Hi-C data at 10-KB resolution were used for look calling for each sample, which was achieved using pyHICCUPS from HiCPeaks packages (Rao et al, 2014). Bedtools pairToPair function was used to compare the loops among samples with two mismatches and identify the overlap between Hi-C loops and ChIP-seq peaks of multiple proteins (Quinlan and Hall, 2010). WT ESC Hi-C loops were crossed with ChIP-seq of Pcgf2 and Ring1b, and the loops with both occupied anchors were considered PRC1 loops. Aggregated plot analysis (APA) plots were generated using the APA analysis function from the HiCPeaks apa-analysis command (Rao et al, 2014).

### 3C library preparation and analysis

The 3C library preparation and analysis were adapted and performed based on a previously published method (Naumova et al, 2012). overnight. The ligated 3C libraries were purified from the nuclei by adding the reverse cross-linking mix (200 mM NaCl and 1.5 mg/mL Proteinase K (Thermo Fisher Scientific, BP1700-100)) and incubating them overnight at 65 °C. About 0.1 mg/mL of RNase A was added to each sample and incubated at 37 °C for 1 h. The DNA was finally extracted using the PCI reagent as described above. The DNA concentration of each 3C library were first standardized before being used for 3C-PCR and qPCR using high-fidelity Phusion DNA polymerase (NEB, M0530S) and iTaq SYBR® Green polymerase (Bio-Rad, 1725121), respectively. The primers used for control and test regions are listed in Appendix Table S4.

### Statistical analyses

For RT-qPCR, 3C-qPCR, MTT assays, and the RING1B and CTCF puncta counts, the statistical significance was determined using biological triplicate data points analyzed with unpaired *t*-test with Welch’s correction and the adjusted *P* values reported based on the Holm–Šídák method. For the mean U2OS CTCF fluorescence intensity, the NPC perimeter measurement, and the number of U2OS RING1B puncta, the statistical significance was calculated using an unpaired *t*-test with Welch’s correction and the two-tailed *P* value was reported. For differences in gene expression and loop strength shown in the box plots, statistical significance was assessed using Student’s *t*-test. To ensure a robust and unbiased analysis, we randomly assigned cell culture plates to treatment groups and applied no post-culture exclusion criteria. Blinding was implemented during image acquisition and subsequent quantitative analysis, with analysts masked to cell line genotypes or treatment. Sample size was determined based on preliminary pilot experiments to ensure sufficient power to detect phenotypic differences.

## Supplementary information

Appendix

Peer Review File

Source data Fig. 1

Source data Fig. 4

Source data Fig. 5

Source data Fig. 7

Expanded View Figures

## Data Availability

Raw and processed next-generation sequencing data generated in this study are deposited at the Gene Expression Omnibus (GEO) under the accession number: GSE278946 (URL: https://www.ncbi.nlm.nih.gov/geo/query/acc.cgi?acc=GSE278946). The source data of this paper are collected in the following database record: biostudies:S-SCDT-10_1038-S44319-026-00885-3.
