# Supplementary material for: cPRC1.2 and CTCF-mediated transition from poised to active chromatin loops at bivalent genes
Source: EMBO Rep. 2026 Jul 23;27(17):5092–120. doi: 10.1038/s44319-026-00885-3 (PMC13554221; doi:10.1038/s44319-026-00885-3)
Supplement: Supplementary file 1 [file 44319_2026_885_MOESM1_ESM.pdf]

cPRC1.2 and CTCF-Mediated Transition from Poised to Active Chromatin Loops at Bivalent Genes

Table of Content

Appendix Figure S1. Hi-C analysis in *WT* and *Pcgf2*$^{-/-}$ ESCs. ……………………..……….2

Appendix Figure S2. Characterization of cPRC1.2 loops. ……………………………….4

Appendix Figure S3. Altered expression of cPRC1.2 loop target genes in *Pcgf2*$^{-/-}$ cells. ………………………………………………………………………………………….5

Appendix Figure S4. SAM domain truncation and Phc1 deletion in ESCs. …………....7

Appendix Figure S5. Enrichment of cPRC1.2 components at Ctcf sites in ESCs. …….9

Appendix Figure S6. Ctcf depletion in ESCs alters critical neuronal gene markers mediated by cPRC1.2. ……………………………………………………………..…10

Appendix Table S1. Hi-C sequencing quality control parameters. ………………..….11

Appendix Table S2. List of cPRC1.2 loop target genes. ………………………………...12

Appendix Table S3. List of antibodies used in this study. …………………………..…19

Appendix Table S4. List of gRNAs, PCR and RT-qPCR primers used in this study. …20

Appendix Table S5. List of ChIP-seq, Hi-ChIP, Hi-C, and RNA-seq datasets used in this study. ……………………………………………………………………………………22

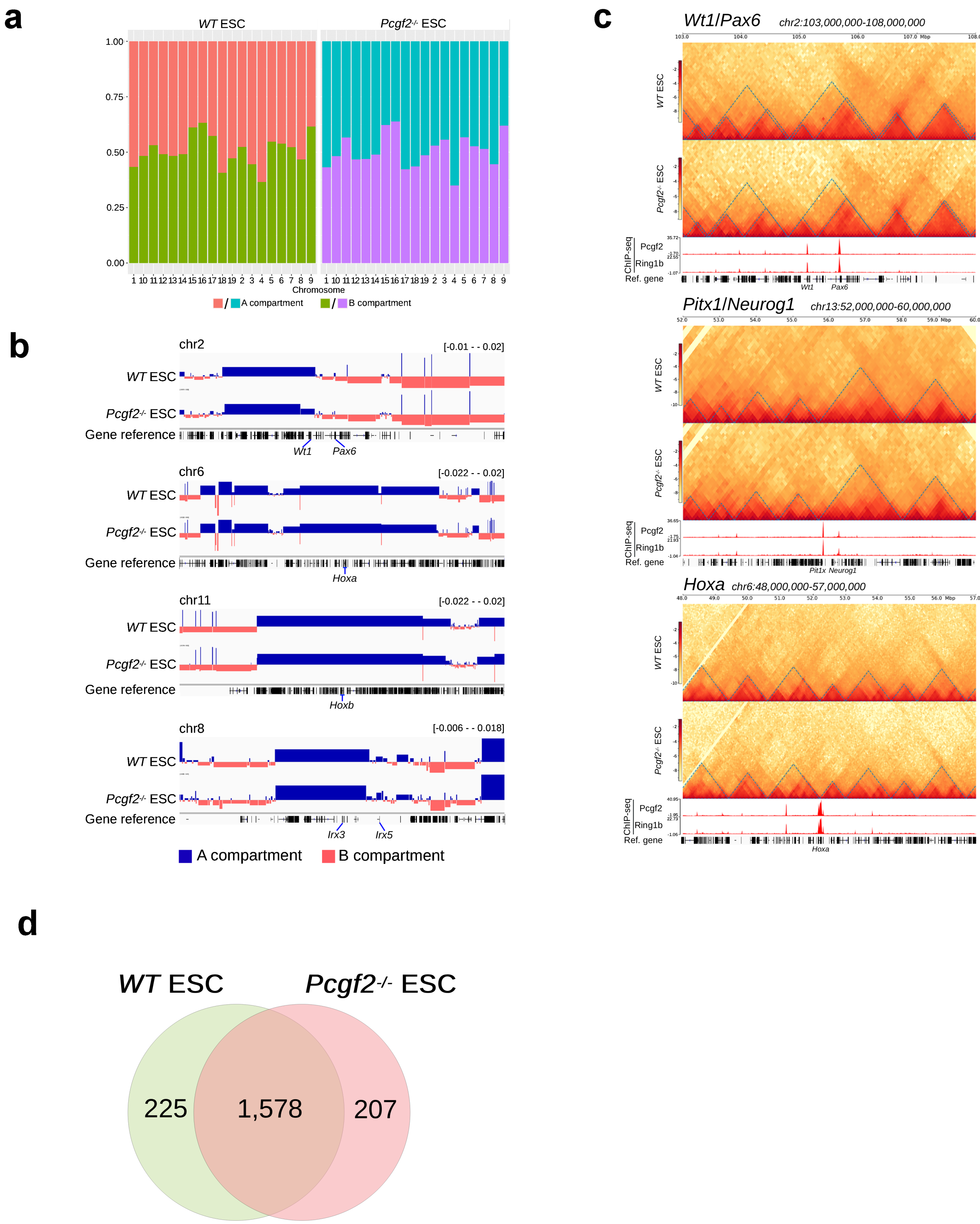
a
WT ESC
Pcgf2-/- ESC
1.00
0.75
0.50
0.25
0.00
Chromosome
A compartment
B compartment
b
chr2
[-0.01 - - 0.02]
WT ESC
Pcgf2-/- ESC
Gene reference
Wt1
Pax6
chr6
[-0.022 - - 0.02]
Hoxa
chr11
[-0.022 - - 0.02]
Hoxb
chr8
[-0.006 - - 0.018]
Irx3
Irx5
A compartment
B compartment
c
Wt1/Pax6 chr2:103,000,000-108,000,000
Pcgf2
Ring1b
ChIP-seq
Ref. gene
Pitx1/Neurog1 chr13:52,000,000-60,000,000
Hoxa chr6:48,000,000-57,000,000
d
WT ESC
Pcgf2-/- ESC
225
1,578
207

Appendix Figure S1. Hi-C analysis in *WT* and *Pcgf2*$^{-/-}$ ESCs. a, A/B compartment analysis for each autosomal chromosome in *WT* and *Pcgf2*$^{-/-}$ ESCs derived from Hi-C data. **b**, A/B compartment plots at the *Wt1*/*Pax6*, *Hoxa*, *Hoxb*, and *Irx3*/*Irx5* loci. **c**, Contact frequency **heatmaps** comparing *WT* and *Pcgf2*$^{-/-}$ ESCs at *Wt1*/*Pax6*, *Pitx1*/*Neurog1*, and *Hoxa* loci. Topologically associated domains (TADs) are highlighted in dashed blue lines. Bottom panel shows ChIP-seq tracks for Ring1b and Pcgf2 in *WT* ESC. **d**, Venn diagram showing the number of unique and shared topologically associated domains (TADs) identified in *WT* and *Pcgf2*$^{-/-}$ ESC samples.

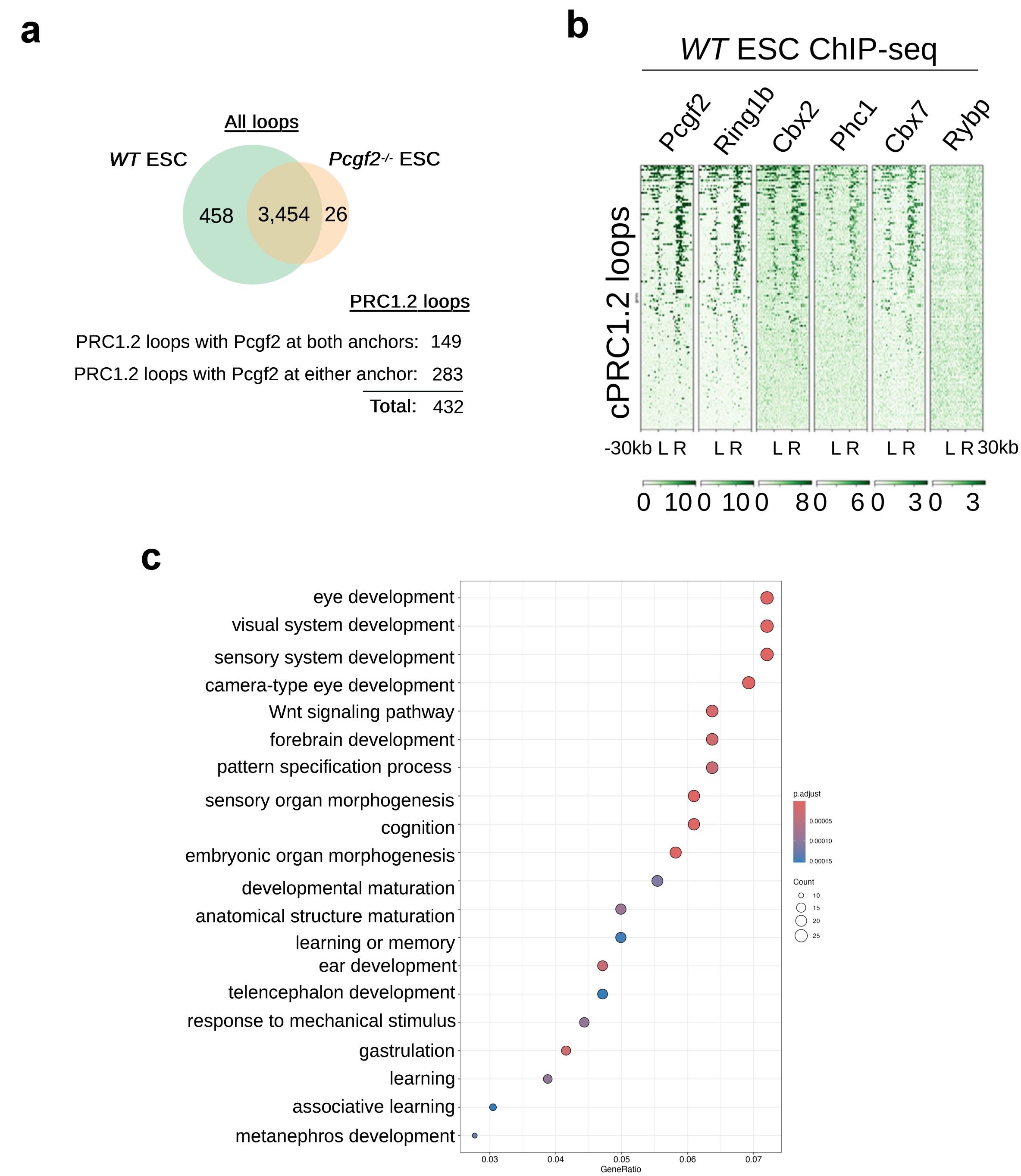

Appendix Figure S2. Characterization of cPRC1.2 loops. a, Venn diagram showing the number of unique and shared chromatin loops in *WT* and *Pcgf2*$^{-/-}$ ESCs. **b**, ChIP-seq heatmaps at 149 cPRC1.2 loops with both anchors occupied with cPRC1.2 components in *WT* ESCs. L and R denote left and right loop anchors, with 30 kb regions flanking each anchor. **c**, GO cPRC1.2 loop-targeted genes. p values are calculated by the hypergeometric distribution embedded in the clusterprofiler package.

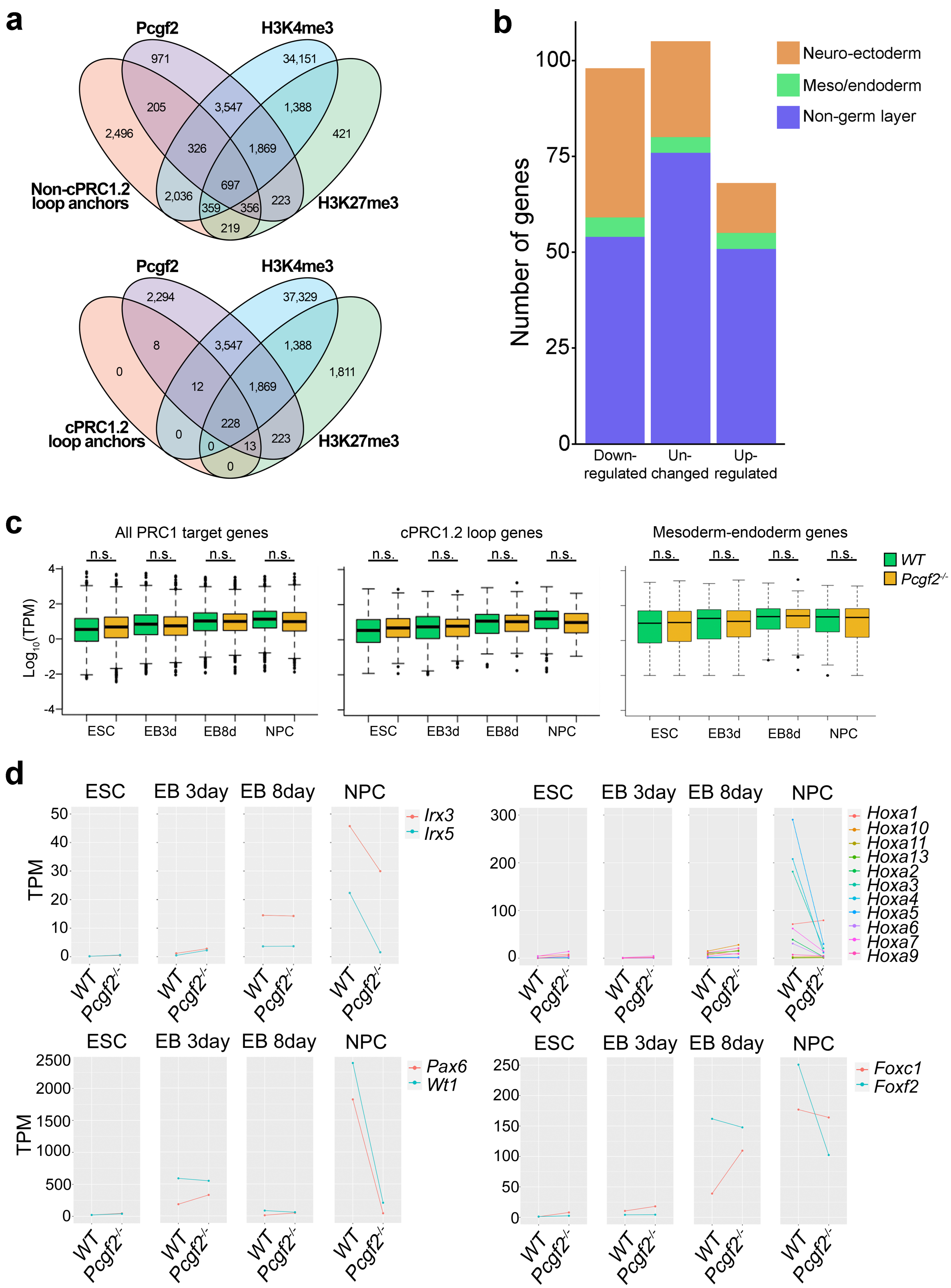

a
Pcgf2
H3K4me3
Non-cPRC1.2 loop anchors
H3K27me3
971
34,151
205
3,547
1,388
2,496
326
1,869
421
697
2,036
359
356
223
219
Pcgf2
H3K4me3
cPRC1.2 loop anchors
H3K27me3
2,294
37,329
8
3,547
1,388
0
12
1,869
1,811
228
0
0
13
223
0
b
Number of genes
Neuro-ectoderm
Meso/endoderm
Non-germ layer
Down-regulated
Un-changed
Up-regulated
c
All PRC1 target genes
cPRC1.2 loop genes
Mesoderm-endoderm genes
n.s.
Log_{10}(TPM)
ESC
EB3d
EB8d
NPC
WT
Pcgf2-/-
d
ESC
EB 3day
EB 8day
NPC
TPM
Irx3
Irx5
Hoxa1
Hoxa10
Hoxa11
Hoxa13
Hoxa2
Hoxa3
Hoxa4
Hoxa5
Hoxa6
Hoxa7
Hoxa9
Pax6
Wt1
Foxc1
Foxf2
WT
Pcgf2-/-

Appendix Figure S3. Altered expression of cPRC1.2 loop target genes in *Pcgf2*$^{-/-}$ cells. a, Venn diagrams show the overlap in localizations of either non-cPRC1.2 loops (top panel) or cPRC1.2 loops (bottom panel) with H3K27me3, H3K4me3, and Pcgf2. **b**, Bar graph shows the percentage distribution of differentially expressed cPRC1.2 loop genes categorized into neuro-ectoderm (39 downregulated, 25 unchanged, and 13 upregulated), meso/endoderm (5 downregulated, 4 unchanged, and 4 upregulated) and non-germ layer (54 downregulated, 76 unchanged, and 51 upregulated) genes between *WT* and *Pcgf2*$^{-/-}$ NPCs. **c**, Box plots show expression for all PRC1 target genes (5,334), total cPRC1.2 loop genes (271), and cPRC1.2 loop targeted meso-endodermal genes (13) in *WT* and *Pcgf2*$^{-/-}$ cells at ESC, EB, and NPC stages. **d**, Line plots showing the transcript per million (TPM) values for genes within *Irx3*/*Irx5*, *Hoxa*, *Wt1*/*Pax6*, and *Foxf2*/*Foxc1* loci from RNA-seq data in *WT* and *Pcgf2*$^{-/-}$ cells at ESC, EB, and NPC stages. Each datapoint represents the average TPM value between duplicates.

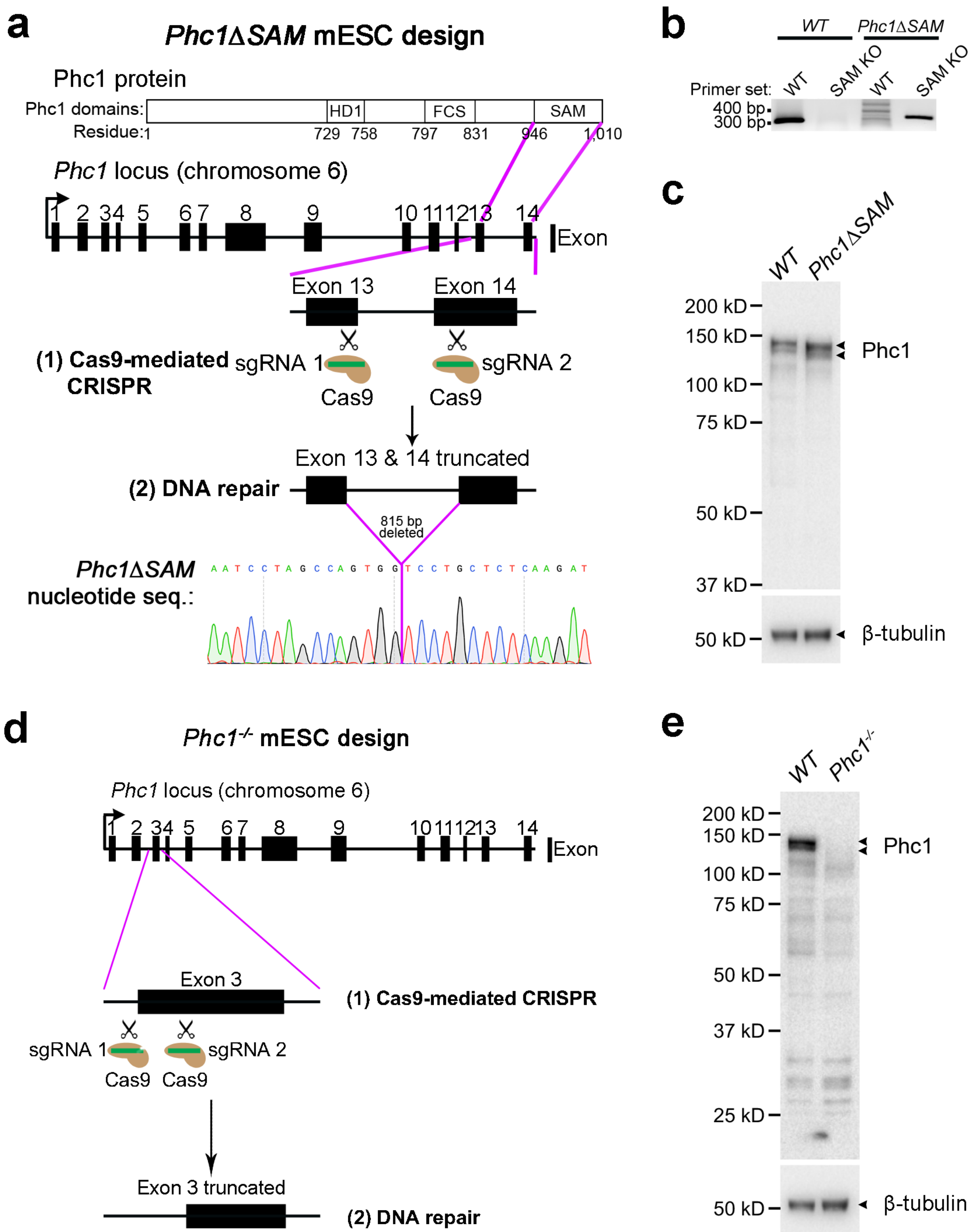
a
Phc1ΔSAM mESC design
Phc1 protein
Phc1 domains:
HD1
FCS
SAM
Residue:
1
729
758
797
831
946
1,010
Phc1 locus (chromosome 6)
1
2
34
5
67
8
9
10
111213
14
Exon
Exon 13
Exon 14
(1) Cas9-mediated CRISPR
sgRNA 1
sgRNA 2
Cas9
Cas9
Exon 13 & 14 truncated
(2) DNA repair
815 bp deleted
Phc1ΔSAM nucleotide seq.:
AATCCTAGCCAGTGGTCCTGCTCTCAAGAT
b
WT
Phc1ΔSAM
Primer set:
WT
SAM KO
WT
SAM KO
400 bp
300 bp
c
WT
Phc1ΔSAM
200 kD
150 kD
100 kD
75 kD
50 kD
37 kD
50 kD
Phc1
β-tubulin
d
Phc1-/- mESC design
Phc1 locus (chromosome 6)
1
2
34
5
67
8
9
10111213
14
Exon
Exon 3
(1) Cas9-mediated CRISPR
sgRNA 1
sgRNA 2
Cas9
Cas9
Exon 3 truncated
(2) DNA repair
e
WT
Phc1-/-
200 kD
150 kD
100 kD
75 kD
50 kD
37 kD
25 kD
50 kD
Phc1
β-tubulin

Appendix Figure S4. SAM domain truncation and Phc1 deletion in ESCs. a, Schematic of Phc1 SAM domain deletion (*Phc1∆SAM*) in ESCs by CRISPR/Cas9-mediated gene editing. The SAM domain spans parts of exons 13 and 14. The bottom panel shows the Sanger sequencing result of a *Phc1∆SAM* ESC clone with SAM domain excision. HD1: homology domain 1; FCS: FCS zinc-finger domain; SAM: sterile alpha motif. **b**, PCR of a *WT* and *Phc1∆SAM* ESC clone using WT and ∆*SAM*-specific primer set showing the successful deletion. **c**, Immunoblots show the smaller size of Phc1 protein from SAM domain truncation in *Phc1∆SAM* ESC. **d**, Schematic showing the complete deletion of Phc1 (Exon 3) in ESCs. **e**, Immunoblots show the loss of Phc1 protein in *Phc1*$^{-/-}$ ESC compared to *WT* ESC.

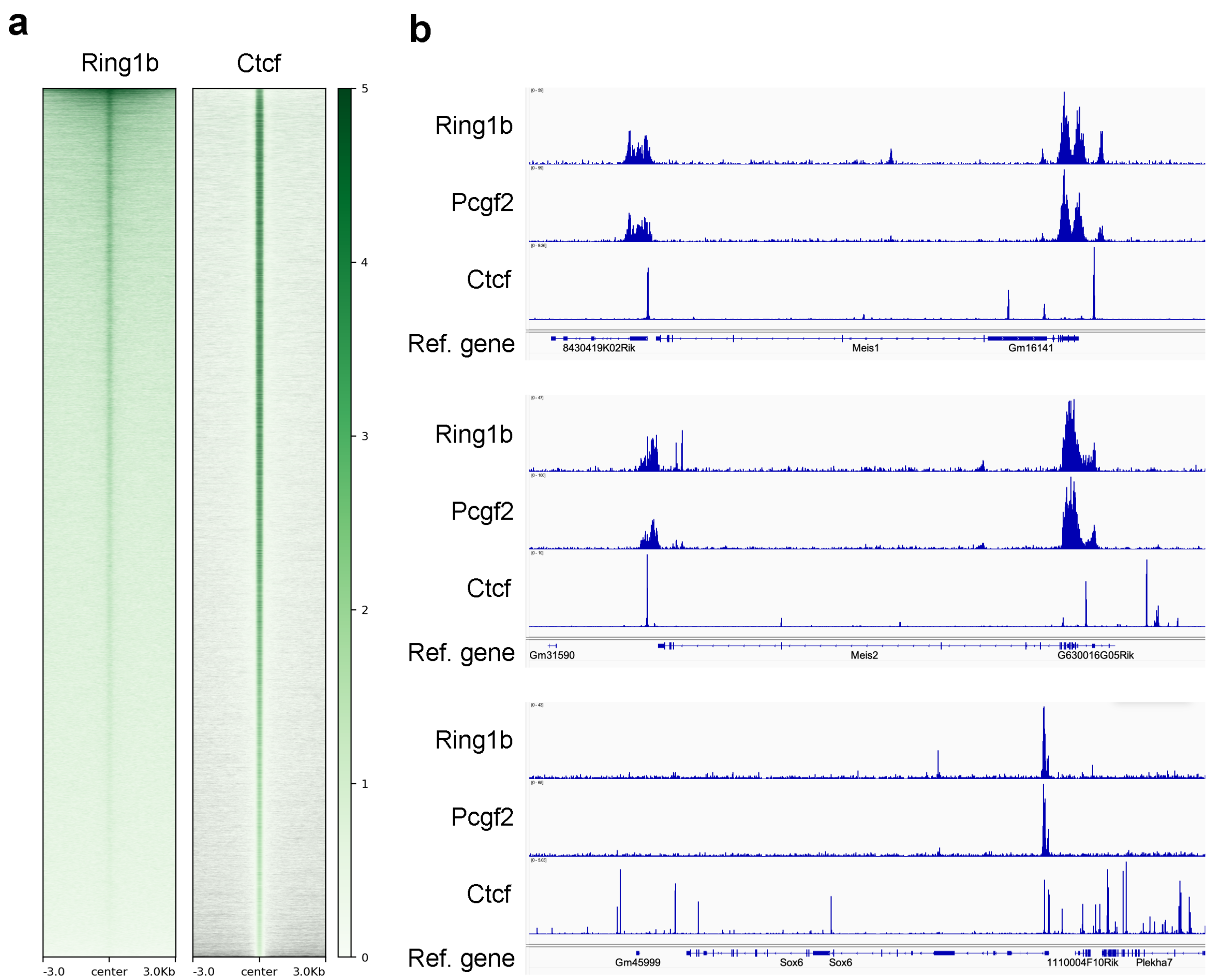

Appendix Figure S5. Enrichment of cPRC1.2 components at Ctcf sites in ESCs. a, ChIP-seq heatmaps at Ctcf-bound sites showing enrichment of Ring1b. **b**, IGV views for selected cPRC1.2 loop-targeted genomic loci showing the co-localization of Ring1b, Pcgf2, and Ctcf.

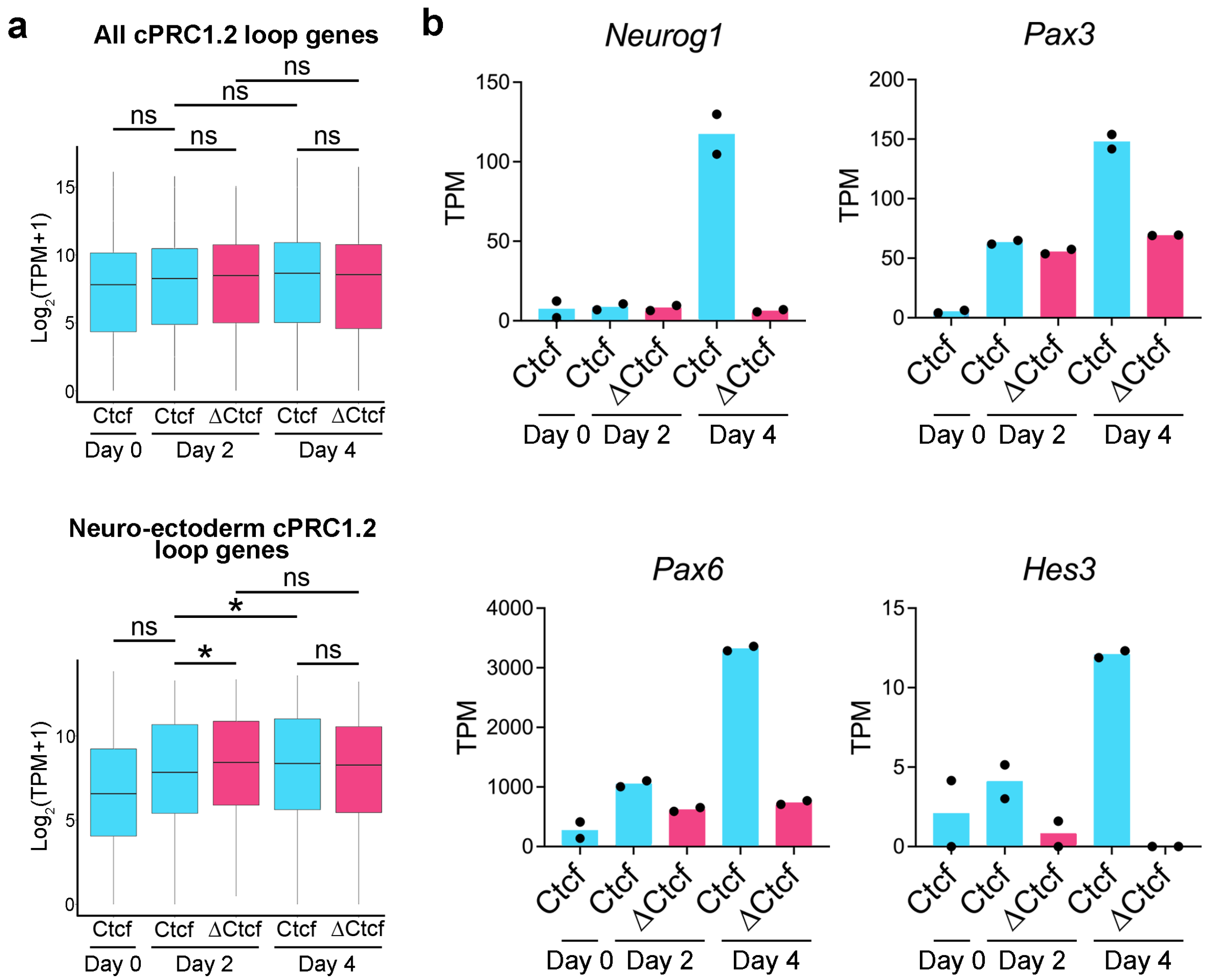

Appendix Figure S6. Ctcf depletion in ESCs alters critical neuronal gene markers mediated by cPRC1.2. a, Box plots show the TPM values of either all cPRC1.2 loop genes (top panel, n = 271) or neuro-ectoderm cPRC1.2 loop genes (bottom panel, n = 77) in control (WT Ctcf) and Ctcf-depleted (ΔCtcf) ESCs, untreated (day 0) or treated with retinoic acid for 2 and 4 days. Boxes show the median (midline) and interquartile range, and whiskers extend to the minimum or maximum data point, or to 1.5 × interquartile range beyond the box, whichever is shorter. A student's t-test was performed. p values are as follows: Top from left to right: 0.73, 0.78, 0.055, 0.087, 0.12; Bottom from left to right: 0.29, 0.034, 0.034, 0.36, 0.12. **b**, Bar graphs show the TPM values of critical neuro-ectoderm markers, *Neurog1*, *Pax3*, *Pax6*, and *Hes3* from day 0 to day 4 of retinoic acid treatment. *$p<0.05$, n.s. $p>0.05$.

Appendix Table S1. Hi-C sequencing quality control parameters.

**Parameter**	***WT* ESC**	***Pcgf2*$^{-/-}$ ESC**	***Pcgf2*$^{-/-}$ NPC**
Total reads	947053322	773871511	698183783
Mapped reads	661722797	539376595	499839643
Mapping ratio	661722797 / 947053322 = 0.6987	539376595 / 773871511 = 0.6970	499839643 / 698183783 = 0.7159
Total reads after filtering	397060244	192827488	406749595
Inter-/trans-chromosomal reads	39437227	19948992	80593419
Intra-/cis-chromosomal reads	357623017	172878496	326156176
Intra-/cis-long range reads	271779607	131376247	211555852

Appendix Table S2. List of cPRC1.2 loop target genes.

gene_id	note
Tnfrsf1a	meso/endo
Tfap2b	meso/endo
Cldn5	meso/endo
Sgce	meso/endo
Dlx3	meso/endo
Foxc2	meso/endo
Foxl1	meso/endo
Zfpm1	meso/endo
Fgf3	meso/endo
Fgf15	meso/endo
Cbx8	meso/endo
Cbx4	meso/endo
Wt1	meso/endo
Pax3	neuron
Anks1	neuron
Foxa1	neuron
Hnf1b	neuron
Fgf20	neuron
Tbx2	neuron
Tbx4	neuron
Irx3	neuron
Irx5	neuron
Tgfb1	neuron
Crtac1	neuron
Foxd2	neuron
Foxe3	neuron
Mycl	neuron
Neurog1	neuron
Stat3	neuron
Six3	neuron
Slc9a3r1	neuron
Kcnq1	neuron
Mfsd2a	neuron
Evx2	neuron
Hoxd11	neuron
Hoxd10	neuron
Hoxd9	neuron

Hoxd8	neuron
Hoxd3	neuron
Pax1	neuron
Rtn4rl2	neuron
Shank1	neuron
Pax2	neuron
Gdpd5	neuron
Bbs2	neuron
Celsr2	neuron
Hoxb9	neuron
Hoxb8	neuron
Hoxb7	neuron
Hoxb6	neuron
Hoxb5	neuron
Hoxb4	neuron
Hoxb3	neuron
Hoxb1	neuron
Plec	neuron
Hes2	neuron
Hes3	neuron
Cck	neuron
Foxo3	neuron
Eomes	neuron
Six1	neuron
Mnat1	neuron
Sox2	neuron
Hoxc10	neuron
Hoxc8	neuron
Vax2	neuron
Tfap2c	neuron
Psen2	neuron
Fgfr3	neuron
Six2	neuron
Cux1	neuron
Tlx1	neuron
Lbx1	neuron
Fbxo2	neuron
Hoxa10	neuron
Hoxa11	neuron

Hoxa13	neuron
Evx1	neuron
Cngb1	neuron
Meis2	neuron
Meis1	neuron
Rac3	neuron
Rab3a	neuron
Tfap2a	neuron
Nfix	neuron
Wnt7a	neuron
Pax6	neuron
Tbx5	neuron
Foxq1	neuron
Fgf5	neuron
Vwf	none
Ttc9	none
Map3k9	none
Ccnd2	none
Parp11	none
Tcp11	none
Bcas1	none
Pfdn4	none
Cyp24a1	none
H2afz	none
Letmd1	none
Pou6f1	none
Plagl1	none
Phactr2	none
Sp3	none
Pcdhb22	none
Pcdhga5	none
Pcdhga6	none
Ddx52	none
Zdhhc2	none
Bckdha	none
Arap3	none
Pcdh1	none
Etfbkmt	none
Resf1	none

Zfp322a	none
Inafm2	none
Disp2	none
Bahd1	none
Slc47a2	none
Slc47a1	none
Arhgap29	none
Btbd16	none
Htra1	none
Cxcl14	none
Slc25a48	none
Gm6525	none
Trim2	none
Ssbp2	none
Gm12830	none
Mrpl19	none
Eva1a	none
Bmp8b	none
Eif3f	none
Tub	none
Actrt3	none
Samd7	none
Tmem50b	none
Pcsk1n	none
Glod5	none
Rarres1	none
Mfsd1	none
Stat5b	none
Cavin1	none
Grin2c	none
Ebf4	none
Cpxm1	none
Mapkapk2	none
Rassf5	none
Bmp1	none
Lgi3	none
Reep4	none
Hr	none
Dmtn	none

Ddx11	none
Mtcl1	none
Osr1	none
Uba2	none
Pdcd2l	none
Ebf1	none
Hoxd13	none
Tex2	none
Hif1an	none
Slc43a1	none
2410002F23Rik	none
Igf2bp3	none
Fam221a	none
Ipmk	none
1700008O03Rik	none
Klhl35	none
Cyp26c1	none
Cyp26a1	none
Myof	none
Marveld3	none
Zfp612	none
Tmc7	none
Gde1	none
Mt1	none
Des	none
Asic4	none
Chpf	none
Sort1	none
Sertm1	none
Ccna1	none
Tmem220	none
Vps45	none
Smarca5-ps	none
Zfp982	none
Nrbp2	none
Aqp2	none
Aqp5	none
Asic1	none
Lyzl4	none

5-Sep	none
Armc2	none
Ttc29	none
Fam43b	none
Camk2n1	none
Casd1	none
Peg10	none
Tac4	none
Dnajc7	none
Zfp385c	none
Dhx58	none
Tnfsf9	none
Cd70	none
Ankle1	none
Abhd8	none
Plvap	none
Ccdc194	none
Ttll6	none
Pom121l2	none
Prss16	none
Hoxc9	none
Smug1	none
Sh2b2	none
Ppp1r15b	none
Kiss1	none
Gm5069	none
Etnk2	none
Prune2	none
Gcnt1	none
Stip1	none
Otub1	none
Rhcg	none
Calm3	none
Pnmal2	none
Ccdc8	none
Cacna2d2	none
Tmem115	none
Sall2	none
Mthfsd	none

Mad2l2	none
Agpat2	none
6430628N08Rik	none
Dmbx1	none
Faah	none
Zfp960	none
Dusp26	none
Rnf122	none
Zfp985	none
Trhr2	none
Zfp319	none
Rassf4	none
Adrb3	none
Eif4ebp1	none
Rgs12	none
Dok7	none
Gjd3	none
Igfbp4	none
Igdcc3	none
Parp16	none
Hsd17b1	none
Rhbg	none
Aars2	none
Tmem151b	none
Map4k1	none
Tnfrsf23	none
Osbpl5	none
Mrgprg	none
Dus1l	none
She	none
Rdm1	none
Arl4d	none
Dhx8	none
Zhx1	none
Atad2	none
Hist2h2bb	none
Fcgr1	none
Gm13212	none
Fbln2	none

Appendix Table S3. List of antibodies used in this study.

Antibody	Manufacturer	Catalog #	Application
Ring1b	Bethyl Laboratories	A302-869A	Immunoblot, immunofluorescence, ChIP-seq
Pcgf2 (Mel18)	Gift from Diego Passini	N/A	Immunoblot, immunofluorescence
Pcgf2 (Mel18)	Generated by Pennsylvania State University College of Medicine Custom Antibody core	N/A	Immunoblot
Rybp	Santa Cruz Biotechnology	SC374235	Immunoblot
Phc1	Cell Signaling Technology	13768	Immunoblot
Pcgf4 (Bmi1)	Bethyl Laboratories	A301-694A	Immunoblot
Gapdh	Invitrogen	MA5-15738	Immunoblot
β-tubulin	Abcam	ab6046	Immunoblot
Nanog	Invitrogen	PA1-41577	Immunoblot
H2AK119 ub1	Cell Signaling Technology	8240	Immunoblot, ChIP-seq
Histone H3	Bethyl Laboratories	A300-823A	Immunoblot
CTCF	Active Motif	91285	ChIP-seq
CTCF	BD Biosciences	BD612148	Immunoblot, immunofluorescence, immunoprecipitation
Alexa Fluor 488	Thermo Fisher Scientific	A28175	Immunofluorescence
Alexa Fluor 568	Thermo Fisher Scientific	A-11011	Immunofluorescence
IgG	Cell Signaling Technology	2729S	Immunoprecipitation

Appendix Table S4. List of gRNAs, PCR and RT-qPCR primers used in this study.

gRNAs	
**Target knock-out**	**gRNA sequence (5' → 3')**
Pcgf2	Guide 1: CACGGATTAAAATCACGGAG
	Guide 2: GTGAGTTGAATTCGGGGGTG
Pcgf4	Guide 1: TTATAAACCGCTCAGCATTC
	Guide 2: TACACAGTGTTCGCCTTCT
Phc1	Guide 1: ACTGCAGCGGCAACCTAATG
	Guide 2: GGGGGAGTTACTGCAAGGTT
Phc1 (SAM domain)	Guide 1: TCCTAGCCAATGGAGCGTCG
	Guide 2: AGTGCCATGAACATCAAATT
Irx3 loop anchor	Guide 1: ATCCAAGGCGTCACTACTCT
	Guide 2: ACCTTCTAGCATTCTGCCCG
**PCR primers**	
**Target knock-out**	**PCR sequence (5' → 3')**
Pcgf2	Forward: GGTGACACTTCCCAAAT
	Reverse: GAGCCAGAAGCTCACTCCTGG
Pcgf4	Forward: ATTCTGATCTCATTAGTAAATTCCTTT
	Reverse: CATAACTGTGAGGTTTACTTTCTTTT
Phc1	Forward: GAGTAGACTACTGAATCCAACATTG
	Reverse: CTGCAACCTTAATCTAAGAACCAC
Phc1 (SAM domain)	Forward (Common): GCCGGGGATCAGATAATTCCA
	Reverse (WT): AATGTGGACCTGGTTCTGCT
	Reverse (KO): GTTCTGCCTCCAAACCCCAC
Irx3 loop anchor	Forward (Common): GCTAGTGGTCTCCATTCTGAGG
	Reverse (WT): TTCGTCCTGTAGAGGGAGCTC
	Reverse (KO): GGCAAACTGGAAAGTAGCGG
**Sanger sequencing primers**	
**Target knock-out**	**Sanger sequencing primer sequence (5' → 3')**
Pcgf2	CCAGGAGTGAGCTTCTGGCTC
Pcgf4	ATTCTGATCTCATTAGTAAATTCCTTT
Phc1 (SAM domain)	CCATCCACGCCAGAGTTACA
Irx3 loop anchor	ATGTGGGCACCGGTCTTCAT
**qPCR primers**	

Species	Gene	Sequence (5' → 3')	Notes
Mouse	*Gapdh*	Forward: GCGGCACGTCAGATCCA	RT-qPCR
		Reverse: CATGGCCTTCCGTGTTCCT	RT-qPCR
Mouse	*Nanog*	Forward: AGGCTTTGGAGACAGTGAGGTG	RT-qPCR
		Reverse: TGGGTAAGGGTGTTCAAGCACT	RT-qPCR
Mouse	*Oct4*	Forward: AGATCACTCACATCGCCAATCA	RT-qPCR
		Reverse: CGCCGGTTACAGAACCATACTC	RT-qPCR
Mouse	*Nes*	Forward: AGTGCCCAGTTCTAGTGGTGTCC	RT-qPCR
		Reverse: CCTCTAAAATAGAGTGGTGAGGGTTG	RT-qPCR
Mouse	*NeuroD1*	Forward: CGAGTCATGAGTGCCCAGCTTA	RT-qPCR
		Reverse: CCGGGAATAGTGAAACTGACGTG	RT-qPCR
Mouse	*Pax6*	Forward: CTTGGGAAATCCGAGACAGA	RT-qPCR
		Reverse: CTAGCCAGGTTGCGAAGAAC	RT-qPCR
Mouse	*Irx3* - *Irx5* loop	Primer 1a: AGAAGGCCTCTGCGTAATGTC	3C-PCR
		Primer 1b: GCGTAATGTCCACTTCCTTCCAAGC	3C-qPCR
		Primer 2a: GCCGCAAATTACAAACGCAC	3C-PCR
		Primer 2b: CAAACGCACTGAAGCTTTGGGACAG	3C-qPCR
		Primer 3a: GGAAATGATGCGACTCCCTCT	3C-PCR
		Primer 3b: GTTGCATGGAAATGATGCGACTCCCTCTG	3C-PCR
		Primer 4a: TTGCCTTGGTCAAAAGGTGC	3C-PCR
		Primer 4b: GAGTGAAAGATGCAGCCACAGA	3C-PCR
Mouse	*Irx5* - Ctcf loop 1	Primer 1a: CACCGAGGCTCCAAGTCATA	3C-PCR
		Primer 1b: GTCATAAATCAAAGGGATGGAGCAGAGC	3C-PCR, 3C-qPCR
		Primer 2a: TGCCTTGGTCAAAAGGTGCAA	3C-PCR
		Primer 2b: GTGGAGTGAAAGATGCAGCCACAGACAGTT	3C-PCR, 3C-qPCR
Mouse	*Irx5* - Ctcf loop 2	Primer 1a: TACCCTAACCCAAACTCCCTG	3C-PCR
		Primer 1b: ACTCCCTGCTTTTACAGACTGG	3C-PCR, 3C-qPCR
		Primer 2a: CTCACTAGCCAGCAAGCAGTT	3C-PCR
		Primer 2b: GCAAGCAGTTTGCTCGGTTG	3C-PCR, 3C-qPCR

Appendix Table S5. List of ChIP-seq, Hi-ChIP, Hi-C, and RNA-seq datasets used in this study.

**Sample**	**GEO/Encode ID**	**Dataset reference**
ChIP-seq Pcgf2 ESC	GSE122715	(Scelfo *et al*, 2019)
ChIP-seq Ring1b ESC	GSE96107	(Bonev *et al*, 2017)
ChIP-seq Ring1b NPC	GSE96107	(Bonev *et al.*, 2017)
ChIP-seq Rybp ESC	GSE42466	(Morey *et al*, 2013)
ChIP-seq Cbx7 ESC	GSE42466	(Morey *et al.*, 2013)
ChIP-seq Cbx2 ESC	GSE89949	(Kundu *et al*, 2017)
ChIP-seq Phc1 ESC	GSE89949	(Kundu *et al.*, 2017)
ChIP-seq CTCF ESC	GSE96107	(Bonev *et al.*, 2017)
ChIP-seq CTCF NPC	GSE96107	(Bonev *et al.*, 2017)
ChIP-seq H3K4me3 ESC	GSE31039 / ENCSR000CGO	(Sethi *et al*, 2020)
ChIP-seq H3K4me3 NPC	GSE96107	(Bonev *et al.*, 2017)
ChIP-seq H3K27me3 ESC	ENCSR059MBO	(Consortium, 2012)
ChIP-seq H3K27me3 NPC	GSE96107	(Bonev *et al.*, 2017)
ChIP-seq H3K27ac ESC	GSE31039 / ENCSR000CGQ	(Sethi *et al.*, 2020)
ChIP-seq H3K27ac NPC	GSE96107	(Bonev *et al.*, 2017)
ChIP-seq H3K4me3 WT ESC	GSE122715	(Scelfo *et al.*, 2019)
ChIP-seq H3K4me3 Pcgf2/4-/- ESC	GSE122715	(Scelfo *et al.*, 2019)
ChIP-seq H3K27me3 WT ESC	GSE122715	(Scelfo *et al.*, 2019)
ChIP-seq H3K27me3 Pcgf2/4-/- ESC	GSE122715	(Scelfo *et al.*, 2019)
Hi-ChIP H3K4me3 ESC	GSE94452	(Kubo *et al*, 2021)
Hi-ChIP H3K4me3 NPC	GSE94452	(Kubo *et al.*, 2021)
Hi-ChIP H3K4me3 Ctcf knockdown ESC	GSE94452	(Kubo *et al.*, 2021)
Hi-ChIP H3K4me3 Ctcf knockdown NPC	GSE94452	(Kubo *et al.*, 2021)
Hi-C mouse ESC	GSE96107	(Bonev *et al.*, 2017)
Hi-C mouse NPC	GSE96107	(Bonev *et al.*, 2017)
RNA-seq Ctcf control ESC	GSE94452	(Kubo *et al.*, 2021)
RNA-seq Ctcf control NPC	GSE94452	(Kubo *et al.*, 2021)
RNA-seq Ctcf knockdown ESC	GSE94452	(Kubo *et al.*, 2021)
RNA-seq Ctcf knockdown NPC	GSE94452	(Kubo *et al.*, 2021)

References

Bonev B, Mendelson Cohen N, Szabo Q, Fritsch L, Papadopoulos GL, Lubling Y, Xu X, Lv X, Hugnot JP, Tanay A *et al* (2017) Multiscale 3D Genome Rewiring during Mouse Neural Development. *Cell* 171: 557-572.e524

Consortium EP (2012) An integrated encyclopedia of DNA elements in the human genome. *Nature* 489: 57-74

Kubo N, Ishii H, Xiong X, Bianco S, Meitinger F, Hu R, Hocker JD, Conte M, Gorkin D, Yu M *et al* (2021) Promoter-proximal CTCF binding promotes distal enhancer-dependent gene activation. *Nat Struct Mol Biol* 28: 152-161

Kundu S, Ji F, Sunwoo H, Jain G, Lee JT, Sadreyev RI, Dekker J, Kingston RE (2017) Polycomb Repressive Complex 1 Generates Discrete Compacted Domains that Change during Differentiation. *Mol Cell* 65: 432-446.e435

Morey L, Aloia L, Cozzuto L, Benitah SA, Di Croce L (2013) RYBP and Cbx7 define specific biological functions of polycomb complexes in mouse embryonic stem cells. *Cell Rep* 3: 60-69

Scelfo A, Fernández-Pérez D, Tamburri S, Zanotti M, Lavarone E, Soldi M, Bonaldi T, Ferrari KJ, Pasini D (2019) Functional Landscape of PCGF Proteins Reveals Both RING1A/B-Dependent-and RING1A/B-Independent-Specific Activities. *Mol Cell* 74: 1037-1052.e1037

Sethi A, Gu M, Gumusgoz E, Chan L, Yan KK, Rozowsky J, Barozzi I, Afzal V, Akiyama JA, Plajzer-Frick I *et al* (2020) Supervised enhancer prediction with epigenetic pattern recognition and targeted validation. *Nat Methods* 17: 807-814
