# Supplementary material for: cPRC1.2 and CTCF-mediated transition from poised to active chromatin loops at bivalent genes
Source: EMBO Rep. 2026 Jul 23;27(17):5092–120. doi: 10.1038/s44319-026-00885-3 (PMC13554221; doi:10.1038/s44319-026-00885-3)
Supplement: Supplementary file 2 [file 44319_2026_885_MOESM2_ESM.pdf]

EMBO reports

cPRC1.2 and CTCF-Mediated Transition from Poised to Active Chromatin Loops at Bivalent Genes

Zhonghua Gao, Aflah Hanafiah, Zhuangzhuang Geng, Tingting Liu, Yen Teng Tai, Wenjie Cai, Qiang Wang, Neil Christensen, Yan Liu, and Feng Yue

Corresponding author(s): Zhonghua Gao (gaozhonghua@suat-sz.edu.cn) , Feng Yue (yue@northwestern.edu)

Review Timeline:

Submission Date:	9th Feb 26
Editorial Decision:	16th Mar 26
Revision Received:	28th May 26
Editorial Decision:	25th Jun 26
Revision Received:	4th Jul 26
Accepted:	13th Jul 26

Editor: Esther Schnapp

Transaction Report:

(Note: With the exception of the correction of typographical or spelling errors that could be a source of ambiguity, letters and reports are not edited. Depending on transfer agreements, referee reports obtained elsewhere may or may not be included in this compilation. Referee reports are anonymous unless the Referee chooses to sign their reports.)

Dear Dr. Gao,

Thank you for the submission of your manuscript to EMBO reports. So far, I could only secure two referees for it, but given that they are in fair agreement that you should be given a chance to revise your ms, I am making a decision now based on the two reports we have and in the interest of time.

As you will see, both referees acknowledge that the findings are potentially interesting. However, they also both point out that the data are not sufficiently strong to support the main conclusions and both referees have several suggestions for how the study could be strengthened. I think all suggestions are good and should be addressed but please let me know in case you disagree and we can discuss the exact revision requirements further, also in a video chat, if you like. The main concern referee 1 raises is the lack of more comprehensive, genome-wide analyses of all identified loops, while referee 2 notes that a more relevant cellular context should be analysed and stronger data are need to support PcG and CTCF co-localization on chromatin.

I would thus like to invite you to revise your manuscript with the understanding that the referee concerns must be fully addressed and their suggestions taken on board. Please address all referee concerns in a complete point-by-point response.

Acceptance of the manuscript will depend on a positive outcome of a second round of review. It is EMBO reports policy to allow a single round of major revision only and acceptance or rejection of the manuscript will therefore depend on the completeness of your responses included in the next, final version of the manuscript.

We realize that it is difficult to revise to a specific deadline. In the interest of protecting the conceptual advance provided by the work, we recommend a revision within 3 months (16th Jun 2026). Please discuss the revision progress ahead of this time with the editor if you require more time to complete the revisions.

IMPORTANT NOTE: we perform an initial quality control of all revised manuscripts before re-review. Your manuscript will FAIL this control and the handling will be DELAYED if the following APPLIES:
1) A data availability section providing access to data deposited in public databases is missing. If you have not deposited any data, please add a sentence to the data availability section that explains that.
2) Your manuscript contains statistics and error bars based on n=2. Please use scatter blots in these cases. No statistics should be calculated if n=2.

When submitting your revised manuscript, please review the submission guidelines (https://link.springer.com/journal/44319/submission-guidelines#cms-Revised-submissions) and carefully review the instructions that follow below. Failure to include requested items will delay the evaluation of your revision.

1) a .docx formatted version of the manuscript text (including legends for main figures, EV figures and tables). Please make sure that the changes are highlighted to be clearly visible.

2) individual production quality figure files as .eps, .tif, .jpg (one file per figure). See hhttps://media.springernature.com/original/springer-cms/rest/v1/content/27825798/data/v1 for more info on how to prepare your figures.

3) We replaced Supplementary Information with Expanded View (EV) Figures and Tables that are collapsible/expandable online. A maximum of 5 EV Figures can be typeset. EV Figures should be cited as 'Figure EV1, Figure EV2" etc... in the text and their respective legends should be included in the main text after the legends of regular figures.

- For the figures that you do NOT wish to display as Expanded View figures, they should be bundled together with their legends in a single PDF file called *Appendix*, which should start with a short Table of Content. Appendix figures should be referred to in the main text as: "Appendix Figure S1, Appendix Figure S2" etc. See 'Expanded View' section of the submission guidelines.

- Additional Tables/Datasets should be labeled and referred to as Table EV1, Dataset EV1, etc. Legends have to be provided in a separate tab in case of .xls files. Alternatively, the legend can be supplied as a separate text file (README) and zipped together with the Table/Dataset file.

4) a .docx formatted letter INCLUDING the reviewers' reports and your detailed point-by-point responses to their comments. As part of the EMBO Press transparent editorial process, the point-by-point response is part of the Review Process File (RPF), which will be published alongside your paper.

5) a complete author checklist, which you can download from our author guidelines. Please insert information in the checklist that is also reflected in the manuscript. The completed author checklist will also be part of the RPF.

6) Please note that all corresponding authors are required to supply an ORCID ID for their name upon submission of a revised

manuscript (<https://orcid.org/>), which can be linked to your profile in the manuscript submission system.

7) Before submitting your revision, primary datasets produced in this study need to be deposited in an appropriate public database. Please remember to provide a reviewer password if the datasets are not yet public. The accession numbers and database should be listed in a formal "Data Availability" section placed after Methods. Please note that the Data Availability Section is restricted to new primary data that are part of this study. * Note - All links should resolve to a page where the data can be accessed. *

If your study has not produced novel datasets, please mention this fact in the Data Availability Section.

8) At EMBO Press we ask authors to provide source data for the main manuscript figures. You will receive a separate email with instructions for providing source data with your revised manuscript, including how to upload and organize the files.

9) Our journal also encourages inclusion of *data citations in the reference list* to directly cite datasets that were re-used and obtained from public databases. Data citations in the article text are distinct from normal bibliographical citations and should directly link to the database records from which the data can be accessed. In the main text, data citations are formatted as follows: "Data ref: Smith et al, 2001" or "Data ref: NCBI Sequence Read Archive PRJNA342805, 2017". In the Reference list, data citations must be labeled with "[DATASET]". A data reference must provide the database name, accession number/identifiers and a resolvable link to the landing page from which the data can be accessed at the end of the reference.

10) Regarding data quantification, the following points must be specified in each figure legend:

- the name of the statistical test used to generate error bars and P values,

- the number (n) of independent experiments (please specify technical or biological replicates) underlying each data point,

- the nature of the bars and error bars (s.d., s.e.m.),

- If the data are obtained from n Program fragment delivered error ``Can't locate object method "less" via package "than" (perhaps you forgot to load "than"?) at //ejpvfs23/sites23b/embor_www/letters/embor_decision_revise_and_review.txt line 54.' 2, use scatter blots showing the individual data points.

Discussion of statistical methodology can be reported in the materials and methods section, but figure legends should contain a basic description of n, P and the test applied.

- Please also include scale bars in all microscopy images.

11) The journal requires a statement specifying whether or not authors have competing interests (defined as all potential or actual interests that could be perceived to influence the presentation or interpretation of an article). In case of competing interests, this must be specified in your disclosure statement.

12) All Materials and Methods need to be described in the main text using our 'Structured Methods' format, which is required for all research articles. According to this format, the Methods section includes a separate Reagents and Tools Table file (listing key reagents, experimental models, software and relevant equipment and including their sources and relevant identifiers) and a Methods and Protocols section describing the methods using a step-by-step protocol format. The aim is to facilitate adoption of the methodologies across labs. More information on how to adhere to this format as well as a downloadable template (.docx) for the Reagents and Tools Table can be found in our author guidelines.

We would also welcome the submission of cover suggestions, or motifs to be used by our Graphics Illustrator in designing a cover.

As part of the EMBO publication's Transparent Editorial Process, EMBO reports publishes online a Review Process File (RPF) to accompany accepted manuscripts. This File will be published in conjunction with your paper and will include the referee reports, your point-by-point response and all pertinent correspondence relating to the manuscript.

You are able to opt out of this by letting the editorial office know (emboreports@embo.org). If you do opt out, the Review Process File link will point to the following statement: "No Review Process File is available with this article, as the authors have chosen not to make the review process public in this case."

I look forward to seeing a revised form of your manuscript when it is ready.

Best regards,
Esther

Esther Schnapp, PhD
Senior Editor
EMBO reports

Referee #1:

In this manuscript from Hanafiah and co-authors the authors explore the role of Pcgf2 and cPRC1.2 in loop formation in ESCs. They construct a model whereby cPRC1.2 is responsible for maintaining loops at bivalent genes that is then activated during NPC differentiation. If the bivalent loops are disrupted, then NPC differentiation is impaired as these genes are no longer available for activation. The model is interesting and compelling, however I have some serious issues with the way the data was analyzed which means it's not clear if the authors data supports the model as presented.

Major comments:
1. Fig 1d. The authors claim H2AK119ub1 'genomic enrichment was decreased' but it's hard to see any change in this figure. Perhaps a pileup would work better. Additionally, I am confused by this section of the text, which says both H2AK119ub1 "showed only a minor reduction" then 'genomic enrichment was decreased, then "global H2AK119ub1 level was not substantially affected".Can the language be unified about if it's changing or not, and presented so that the changes can be easily seen.

2. It's not clear what the Pcgf2 cells are differentiating to when exposed to NPC differentiation protocol. Identification of the cell type they differentiate to would help give some context. Is it a mixture of non-neuronal cell fates?

3. Of the 458 lost loops, are one or either end associated with neuronal gene sets (instead of just anecdotal genes), e.g. by GO analysis? Table S2 alone is inadequate.

4. Fig 2a is used to support the claim Pcgf2 and Ring1b are enriched at anchors, but a systematic analysis of the 458 differential loops would be better.

5. Again, Fig 2a is used to support the claim H3K4me3 and H3K27me3 are enriched at these anchors. But a systemic analysis like in Fig 2c, but for the 458 lost loops would be more helpful. These are key claims in the paper about bivalent gene establishment/maintenance, but the claim these genes are bivalent is not well supported by the data.

6. Fig 2: What about H2AK119ub1? Please add to the analysis, at least confirm it does not change too much.

7. In general I found Figure 2 and 3 to be problematic. The authors have the epigenetic data in the KO cells, but don't analyse it. What (if any) epigenetic changes are driving/correlated with the lost loops? By integrating the KO epigenetic data it should be possible to build a model for how epigenetic changes at bivalent genes are disrupting loops. As it is, Fig 2 only says loops are disrupted in the Pcgf2 KO cells, but does not provide a reason. Indeed, section 4 begins "Our observation of bivalent histone marks at selected cPRC1.2 ...'. But this has not been established and the analysis of H3K4me3 and H3K27me3 is restricted to a few anecdotal examples in Fig 2a and the KOS are not analyzed in Fig 3b. The authors must answer: 1. Are disregulted loops at bivalent loci? 2. What impact does Pcgf2 KO have on bivalency at disregualted loops? 3. Does this relate to the changes in loops seen in the KOs?

8. Both 'PRC1.2 and cPRC1.2' are used to describe the loops. A unified nomenclature would be helpful here, rather than changing the name of the loops because Rybp wasn't present (or, determine this first and so keep the cPRC1.2 name?). Later in Fig 3a, it seems all loops are now being used? As it now says 6,694 loop anchors? Wouldn't it be better to focus the entire manuscript on the differential loops so as to build a mechanistic chain from Pcgf2, to bivalency, to loop disruption to gene expression changes and finally to phenotype changes?

9. The sentence "Pcgf2 is also associated with ncPRC1 components such as Rybp" is confusing in this context as the authors have already shown Rybp is not important (Figure 2c).

10. THe PCA plot is very interesting, but the labelling and color selection is poor making it difficult to interpret. It seems to suggest that ESCs are invariant to Pcgf2 KO, and does not support the authors assertion that the Phc1dSAM is a phenocopy of the Pcgf2 KO. A cross-correlation heatmap of gene expression might be useful to explore the similarities. Similarly, some heat maps of selected neuronal genes would be useful in the different cell types to see specific changes.

11. Fig 5a: Is this correct? It appears to show no enrichment for Pcgf2 and Ring1b ad cPRC1.2 anchors, despite these factors being used to define these peaks?

12. Fig 5a: Similarly, these plots reject the idea that CTCF is co-bound at these loops.

13. The Virtual4C is useful, can these sorts of analysis be added to earlier figures as well, e.g. Fig 2, to increase the clarity.

14. Fig 6c: A common problem in this manuscript is the use of single anecdotal loci to make sweeping claims which are not explored systematically genome wide. The pattern is much better if a systematic analysis is done, a conclusion made and then an anecdotal loci used to illustrate the effect. In Fig 6c the authors are trying to establish a link to H3K27ac, but this is only explored at a single locus.

15. Fig 8: The model is interesting, but I find it only partially supported at present due to missing or incomplete genome-wide analysis for NPC genes. The authors did not conclusively establish bivalency at cPRC1.2, and how that changes when Pcgf2 is KOd. The link to CTCF is weak and not established in a systemic way. Finally, a clear path from Pcgf2 binding, to bivalent loci, to epigenetic changes to loop disruption to phenotype has not been established. This is despite all f the data being available in this manuscript to come to these conclusions. Note that above I do not ask for any extra experiments, just improved more systematic analysis.

Minor comments:
1. In the methods specific command line options used for things like bwa, MACS, etc. should be specified.

Referee #2:

In this manuscript, the authors investigate the role of PCGF2-containing Polycomb (PcG) bodies in neural differentiation, focusing on transcriptional regulation and chromatin organization during lineage transition. By combining genetic perturbation with genomic analyses, the authors identify a set of genes whose activation during neural differentiation appears to depend on PCGF2. In addition, the authors explore the relationship between PCGF2 and CTCF and propose that co-localization between these factors contributes to the activation of neuro-ectodermal genes during differentiation.
Overall, the manuscript contains several interesting observations. In particular, the identification of PCGF2-dependent transcriptional responses during neural differentiation is potentially relevant to understanding functional specialization among PRC1 complexes. The datasets presented here may therefore be of interest to researchers studying Polycomb-mediated gene regulation.
However, I find that several aspects of the mechanistic interpretation appear overstated, and in some cases the conclusions are not fully supported by the data presented. In particular, the proposed relationship between PcG complexes and CTCF requires further clarification. While the observations themselves appear sound, alternative explanations are not sufficiently considered.
In my view, the manuscript could be suitable for publication in EMBO Reports, provided that the interpretation of several key results is moderated and that the analyses supporting the proposed model are strengthened. I hope the comments below will help the authors improve the manuscript.

Major comments

1. Interpretation of PcG-CTCF co-localization (Figure 5)
The proposed mechanistic interpretation of the relationship between CTCF and PcG binding sites may require further clarification.
In the current manuscript, the model appears to rely heavily on analyses performed in U2OS cells, whereas the biological context of the study concerns neural differentiation of ESCs. Because PcG occupancy and chromatin architecture can vary substantially across cell types, it is not entirely clear whether the proposed PcG-CTCF relationship observed in U2OS cells can be directly generalized to the differentiation system studied here.
In particular, comparisons with ChIP profiles from ESCs or NPCs would be important to determine whether similar PcG-CTCF associations are present in the relevant developmental context. Without such comparisons, it remains difficult to evaluate whether the proposed mechanism reflects a general regulatory principle or a cell-type-specific phenomenon.
Furthermore, the interpretation assumes that PcG and CTCF co-occupy the same genomic loci in cis, based largely on ChIP peak overlap and immunostaining. However, these observations do not exclude the possibility that the apparent proximity arises from long-range chromatin interactions, such as contacts between CTCF-bound sites and PcG-bound CpG islands.
Given the well-established role of CTCF in mediating long-range chromatin interactions, this alternative explanation should also be considered.
To better support the proposed model, it would therefore be helpful if the authors could provide additional analyses, such as:
1) Heatmaps showing RING1B signal centered on CTCF sites (and vice versa)
2) Representative genomic browser snapshots demonstrating peak co-localization
3) Quantitative analyses distinguishing cis co-binding from long-range interactions

2. Interpretation of transcriptional changes in Figure 1
In Figure 1A, the authors identify genes whose activation during neural differentiation depends on PCGF2. However, the data appear to reveal two distinct transcriptional tendencies, where some genes show reduced activation in PCGF2-deficient cells, whereas others appear to become more active.
Despite this bidirectional transcriptional behavior, the subsequent discussion largely interprets the relationship between

Polycomb and CTCF in the context of transcriptional activation alone. This interpretation seems somewhat oversimplified. Given that both activation and derepression patterns appear to be present, it would be important to consider whether the observed transcriptional effects reflect multiple regulatory modes of PcG complexes rather than a single activation mechanism. Moreover, since the PcG-CTCF relationship is primarily analyzed in U2OS cells, the manuscript should clarify how these observations relate to the transcriptional changes observed during neural differentiation.
A more balanced discussion of these transcriptional patterns would help place the findings in a clearer mechanistic framework.

Minor comments

1. Chromatin organization statement (page 3, line 6)
The statement that chromatin compartments and TADs show high evolutionary conservation may be somewhat misleading depending on the references cited. TAD conservation across species remains debated and appears to depend strongly on the scale of analysis. The authors may wish to verify the cited literature and adjust the wording accordingly.

2. Novelty of Figure 2
The analyses presented in Figure 2 appear largely consistent with previously reported features of PcG occupancy and chromatin states. While these data are useful for contextualizing the study, their novelty appears somewhat limited. The authors may consider clarifying whether these analyses are intended primarily as validation of previously reported observations.

3. Quantification of PcG-CTCF overlap
The manuscript frequently refers to co-localization between PcG and CTCF based on ChIP peak overlap. It would be useful if the authors could provide quantitative assessments of this overlap, for example by calculating enrichment relative to randomized genomic regions or by using permutation-based analyses. Such analyses would help demonstrate whether the observed overlap is statistically meaningful.
5. Distinguishing correlation from causation
More generally, several conclusions rely primarily on correlations between PcG binding, CTCF occupancy, and transcriptional activation. While these correlations are interesting, the manuscript would benefit from a clearer distinction between correlation and causation in the interpretation of these findings.

Response to reviewers (EMBOR-2026-63823V1)

Dear Editor and Reviewers,

We thank the reviewers for their valuable comments and suggestions. In response, we have extensively revised our manuscript by conducting additional analyses of existing data and public datasets published by others. These revisions strengthen our conclusions about how PRC1-mediated chromatin loops promote CTCF-mediated active loops and the subsequent activation of genes critical for neuronal differentiation. We hope you may find the manuscript is now significantly improved and worthy of publication. Below, we provide a point-by-point response to the reviewers' concerns.

Referee #1:

In this manuscript from Hanafiah and co-authors the authors explore the role of Pcgf2 and cPRC1.2 in loop formation in ESCs. They construct a model whereby cPRC1.2 is responsible for maintaining loops at bivalent genes that is then activated during NPC differentiation. If the bivalent loops are disrupted, then NPC differentiation is impaired as these genes are no longer available for activation. The model is interesting and compelling, however I have some serious issues with the way the data was analyzed which means it's not clear if the authors data supports the model as presented.

Major comments:

1. Fig 1d. The authors claim H2AK119ub1 'genomic enrichment was decreased' but it's hard to see any change in this figure. Perhaps a pileup would work better. Additionally, I am confused by this section of the text, which says both H2AK119ub1 "showed only a minor reduction" then 'genomic enrichment was decreased, then "global H2AK119ub1 level was not substantially affected".Can the language be unified about if it's changing or not, and presented so that the changes can be easily seen.

Response: H2AK119ub1 does not show an obvious change, as the reviewer pointed out. By saying 'genomic enrichment was decreased', we meant for Ring1b. We have revised the text to clarify this confusion. It now reads "……but the genomic enrichment of Ring1b was decreased (Fig. 1d). "

2. It's not clear what the Pcgf2 cells are differentiating to when exposed to NPC differentiation protocol. Identification of the cell type they differentiate to would help give some context. Is it a mixture of non-neuronal cell fates?

Response: **As the reviewer rightly pointed out, the cells differentiated from Pcgf2 KO ESCs are likely a mixture of non-neuronal cells. Given the nature of our RNA-seq analysis, it would be challenging to determine the identity of these differentiated cells. We acknowledge this as a limitation of the current study. Resolving the cellular heterogeneity would indeed require a substantial effort, such as scRNA-seq analysis, and we intend to pursue this in follow-up work.**

3. Of the 458 lost loops, are one or either end associated with neuronal gene sets (instead of just anecdotal genes), e.g. by GO analysis? Table S2 alone is inadequate.

Response: **Among 458 lost loops, a substantial number of them are associated with neuronal genes as listed in Table S2. As suggested by the reviewer, we conducted a GO analysis on these loop-targeted genes, and the results showed a clear enrichment of gene terms associated with neurodevelopment, including "eye development", "forebrain development", "sensory organ morphogenesis", and "telencephalon development", as shown in new Fig. S2c.**

4. Fig 2a is used to support the claim Pcgf2 and Ring1b are enriched at anchors, but a systematic analysis of the 458 differential loops would be better.

Response: **We agree with the reviewer that a more thorough analysis of all cPRC1.2 loops would give a better understanding of the correlation of cPRC1.2 and loops. In our original submission, we performed a systematic analysis on 149 loops with both anchors occupied by cPRC1.2 (old Fig. 2c, now Fig. S2b), in which we plotted the ChIP-seq intensities of cPRC1.2 subunits, including Pcgf2, Ring1b, Cbx2, Phc1, and Cbx7. These loops are a subset of the total 432 cPRC1.2 loops lost in Pcgf2 knockout ESCs, which also include loops with only one anchor enriched with cPRC1.2. We have now replaced this heatmap with a new one that includes all 432 cPRC1.2 loops, as shown in the new Fig. 2b. We have also kept the 149-loop heatmap in the supplemental figure, Fig. S2b. We believe this new analysis strengthens our finding that cPRC1.2 components are enriched at loop anchors.**

5. Again, Fig 2a is used to support the claim H3K4me3 and H3K27me3 are enriched at these anchors. But a systemic analysis like in Fig 2c, but for the 458 lost loops would be more helpful.

These are key claims in the paper about bivalent gene establishment/maintenance, but the claim these genes are bivalent is not well supported by the data.

Response: **As the reviewer suggested, we have extended our analysis to all 432 cPRC1.2 loops and found the co-occupancy of H3K4me3 and H3K27me3 at loop anchors, as shown in new Fig. 3b.**

6. Fig 2: What about H2AK119ub1? Please add to the analysis, at least confirm it does not change too much.

Response: **We have generated a heatmap of H2AK119ub1 alongside H3K4me3 and H3K27me3 for all 432 cPRC1.2 loops. Our new analysis showed no obvious difference in H2AK119ub1 between WT and Pcgf2 knockout ESCs (Fig. 3b).**

7. In general I found Figure 2 and 3 to be problematic. The authors have the epigenetic data in the KO cells, but don't analyse it. What (if any) epigenetic changes are driving/correlated with the lost loops? By integrating the KO epigenetic data it should be possible to build a model for how epigenetic changes at bivalent genes are disrupting loops. As it is, Fig 2 only says loops are disrupted in the Pcgf2 KO cells, but does not provide a reason. Indeed, section 4 begins "Our observation of bivalent histone marks at selected cPRC1.2 ...'. But this has not been established and the analysis of H3K4me3 and H3K27me3 is restricted to a few anecdotal examples in Fig 2a and the KOS are not analyzed in Fig 3b. The authors must answer: 1. Are disregulted loops at bivalent loci? 2. What impact does Pcgf2 KO have on bivalency at disregualted loops? 3. Does this relate to the changes in loops seen in the KOs?

Response: **In response to the reviewer's suggestions, we performed an analysis using previously published ChIP-seq data (GSE122715) for H3K27me3 and H3K4me3 in WT and Pcgf2/4 KO ESCs. We have not done H3K27me3 and H3K4me3 ChIP-seq in Pcgf2 KO ESCs ourselves. We believe the Pcgf2 and Pcgf4 double knockout should give a comparable assessment for the impact of loss of cPRC1.2 on bivalency, given the similarity in composition between cPRC1.2 and cPRC1.4. Our new analysis shows no obvious change in H3K27me3 and H3K4me3 enrichment at cPRC1.2 loop anchors between WT and Pcgf2/4 KO cells (Fig. 3b). Therefore, we reasoned that, although the loops depend on the presence of cPRC1.2, they are not a direct result of the bivalent marks. This analysis should answer questions 2 and 3 in this part of the comments. To question 1, our new analysis in Fig. 3b shows that cPRC1.2 loops that are disrupted in Pcgf2 KO ESCs are simultaneously enriched with both H3K27me3 and H3K4me3 marks.**

8. Both 'PRC1.2 and cPRC1.2' are used to describe the loops. A unified nomenclature would be helpful here, rather than changing the name of the loops because Rybp wasn't present (or, determine this first and so keep the cPRC1.2 name?). Later in Fig 3a, it seems all loops are now being used? As it now says 6,694 loop anchors? Wouldn't it be better to focus the entire manuscript on the differential loops so as to build a mechanistic chain from Pcgf2, to bivalency, to loop disruption to gene expression changes and finally to phenotype changes?

Response: **We have revised our nomenclature and consistently used cPRC1.2 loops throughout the manuscript. As suggested by the reviewer, we determined the absence of the non-canonical component Rybp earlier by switching Fig 2b and 2c.**

In Fig 3a, only cPRC1.2 loops were used. We edited the figure legends to make this clear. Throughout the manuscript, we have focused on cPRC1.2-dependent differential loops to establish a clear and logical analytical flow.

9. The sentence "Pcgf2 is also associated with ncPRC1 components such as Rybp" is confusing in this context as the authors have already shown Rybp is not important (Figure 2c).

Response: **We included this sentence in the manuscript, intending to point out that Pcgf2 is associated with both cPRC1 and ncPRC1, whereas Phc1 is specifically associated with cPRC1. We have changed the original sentence to 'Pcgf2 is present in both cPRC1 and ncPRC1' to avoid confusion.**

10. THe PCA plot is very interesting, but the labelling and color selection is poor making it difficult to interpret. It seems to suggest that ESCs are invariant to Pcgf2 KO, and does not support the authors assertion that the Phc1dSAM is a phenocopy of the Pcgf2 KO. A cross-correlation heatmap of gene expression might be useful to explore exploring similarities. Similarly, some heat maps of selected neuronal genes would be useful in the different cell types to see specific changes.

Response: **We have modified the labeling with combinations of shapes and colors for better visualization (Fig. 4d).**

Neither deletion of Pcgf2 nor deletion of SAM domain of Phc1 caused a dramatic change in gene expression in ESCs. The change was much more pronounced when cells were induced to differentiate into NPCs. Interestingly, in NPCs, Pcgf2 KO had a more dramatic effect than Phc1∆SAM. Given that Pcgf2 is present in both cPRC1 and ncPRC1, but Phc1 is only associated

with cPRC1, we reasoned that Pcgf2 KO disrupts both cPRC1 and ncPRC1, whereas Phc1ΔSAM affects only cPRC1. As a result, Pcgf2KO caused a broader effect than Phc1ΔSAM.

As the reviewer suggested, we have generated heat maps of selected neuronal genes (Fig. EV3e) to better visualize the change in specific gene expression.

<u>11. Fig 5a: Is this correct? It appears to show no enrichment for Pcgf2 and Ring1b ad cPRC1.2 anchors, despite these factors being used to define these peaks?</u>

Response: Fig 5a shows enrichment near cPRC1.2 loop anchors, with the ChIP-seq peak shifted from the center of the anchor regions. Given the Hi-C resolution, it is difficult to precisely locate the loop anchors. Therefore, we used a 10-kb genomic window to assess overlap between Hi-C data and ChIP-seq peaks. Consequently, the ChIP-seq peaks do not exactly match the loop anchors. However, both Pcgf2 and Ring1b show a similar pattern across the same loop anchor regions, suggesting the enrichment of cPRC1.2 complex at these sites.

<u>12. Fig 5a: Similarly, these plots reject the idea that CTCF is co-bound at these loops.</u>

Response: We conducted a similar analysis for Ctcf as done for Ring1b and Pcgf2. The Ctcf genomic occupancy at cPRC1.2 loop anchor regions mimics the distribution of Pcgf2 and Ring1b (Fig. 5a, top). However, when we analyzed control regions with no Ring1b and Pcgf2 enrichment, we don't see Ctcf enrichment (Fig. 5a, bottom). These results strongly suggest that Ctcf tends to co-localize with cPRC1.2 at loop anchors.

<u>13. The Virtual4C is useful, can these sorts of analysis be added to earlier figures as well, e.g. Fig 2, to increase the clarity.</u>

Response: We agree that Virtual 4C is a useful tool for visualizing specific interactions from Hi-C data. Therefore, we have added the corresponding Virtual 4C panels in Fig. 2a, as the reviewer suggested. This analysis further supports our claimed requirement of Pcgf2 for the formation of cPRC1.2 loops in ESCs.

<u>14. Fig 6c: A common problem in this manuscript is the use of single anecdotal loci to make sweeping claims which are not explored systematically genome wide. The pattern is much better if a systematic analysis is done, a conclusion made and then an anecdotal loci used to illustrate the effect. In Fig 6c the authors are trying to establish a link to H3K27ac, but this is only explored at a single locus.</u>

Response: We agree with the reviewer that a systematic evaluation of H3K27ac could strengthen our conclusion. In our revised manuscript, we have included a global analysis of H3K27ac enrichment at newly formed CTCF loops during differentiation. As shown in Fig. 6f, the averaging H3K27ac signals across newly gained CTCF loops in NPCs show a higher enrichment than those in ESCs (Fig. 6f).

15. Fig 8: The model is interesting, but I find it only partially supported at present due to missing or incomplete genome-wide analysis for NPC genes. The authors did not conclusively establish bivalency at cPRC1.2, and how that changes when Pcgf2 is KOd. The link to CTCF is weak and not established in a systemic way. Finally, a clear path from Pcgf2 binding, to bivalent loci, to epigenetic changes to loop disruption to phenotype has not been established. This is despite all f the data being available in this manuscript to come to these conclusions. Note that above I do not ask for any extra experiments, just improved, more systematic analysis.

Response: We appreciate the reviewer's comments and suggestions. As we have described in response to the above specific comments, we have provided additional genome-wide analyses to complement observations at specific loci to enhance our conclusions. We have analyzed all cPRC1.2 loop-target sites and shown that H3K4me3 and H3K27me3 are simultaneously enriched and unaffected by Pcgf2 knockout (Fig. 3b), which disrupts looping, suggesting that bivalency precedes cPRC1.2-mediated long-range interactions. To improve the formation of subsequent CTCF loops, we analyzed H3K27ac enrichment at newly formed CTCF loops upon neuronal differentiation (Fig. 6f), which shows clearly higher enrichment in NPCs. Together with our other efforts to address the impact of loop disruption on the global chromatin features and gene expression described above for specific comments, we believe we have substantially strengthened our manuscript.

Minor comments:

1. In the methods specific command line options used for things like bwa, MACS, etc. should be specified.

Response: We have added the suggested information related to RNA-seq, ChIP-seq, and Hi-C analysis to the Methods section.

Referee #2:

In this manuscript, the authors investigate the role of PCGF2-containing Polycomb (PcG) bodies

in neural differentiation, focusing on transcriptional regulation and chromatin organization during lineage transition. By combining genetic perturbation with genomic analyses, the authors identify a set of genes whose activation during neural differentiation appears to depend on PCGF2. In addition, the authors explore the relationship between PCGF2 and CTCF and propose that co-localization between these factors contributes to the activation of neuro-ectodermal genes during differentiation.

Overall, the manuscript contains several interesting observations. In particular, the identification of PCGF2-dependent transcriptional responses during neural differentiation is potentially relevant to understanding functional specialization among PRC1 complexes. The datasets presented here may therefore be of interest to researchers studying Polycomb-mediated gene regulation.

However, I find that several aspects of the mechanistic interpretation appear overstated, and in some cases the conclusions are not fully supported by the data presented. In particular, the proposed relationship between PcG complexes and CTCF requires further clarification. While the observations themselves appear sound, alternative explanations are not sufficiently considered.

In my view, the manuscript could be suitable for publication in EMBO Reports, provided that the interpretation of several key results is moderated and that the analyses supporting the proposed model are strengthened. I hope the comments below will help the authors improve the manuscript.

Major comments

1. Interpretation of PcG-CTCF co-localization (Figure 5)

The proposed mechanistic interpretation of the relationship between CTCF and PcG binding sites may require further clarification.

In the current manuscript, the model appears to rely heavily on analyses performed in U2OS cells, whereas the biological context of the study concerns neural differentiation of ESCs. Because PcG occupancy and chromatin architecture can vary substantially across cell types, it is not entirely clear whether the proposed PcG-CTCF relationship observed in U2OS cells can be directly generalized to the differentiation system studied here.

In particular, comparisons with ChIP profiles from ESCs or NPCs would be important to determine whether similar PcG-CTCF associations are present in the relevant developmental context. Without such comparisons, it remains difficult to evaluate whether the proposed mechanism reflects a general regulatory principle or a cell-type-specific phenomenon.

Furthermore, the interpretation assumes that PcG and CTCF co-occupy the same genomic loci in cis, based largely on ChIP peak overlap and immunostaining. However, these observations do not exclude the possibility that the apparent proximity arises from long-range chromatin

interactions, such as contacts between CTCF-bound sites and PcG-bound CpG islands. Given the well-established role of CTCF in mediating long-range chromatin interactions, this alternative explanation should also be considered.

To better support the proposed model, it would therefore be helpful if the authors could provide additional analyses, such as:

1) Heatmaps showing RING1B signal centered on CTCF sites (and vice versa)

2) Representative genomic browser snapshots demonstrating peak co-localization

3) Quantitative analyses distinguishing cis co-binding from long-range interactions

Response: We appreciate these constructive comments and suggestions. We agree that performing additional analyses is important to strengthen the link between cPRC1.2 and CTCF.

1) First, as the reviewer suggested, in addition to U2OS microscopic analysis, we should conduct ChIP-seq in ESCs to compare the localization of PRC1 and Ctcf. In fact, we analyzed Ctcf enrichment at cPRC1.2 loop anchors, as reflected by Pcgf2 and Ring1b peaks in Fig. 5a, but we did not clearly state which cell types we analyzed. We have now edited the text and figure legends to clarify that this analysis was conducted in ESCs. We also provided additional analysis on Ring1b signals around Ctcf peaks, as shown in new Fig. S5a, which shows, albeit relatively weak, colocalization of Ring1b. We reasoned that this may be because, in general, CTCF and PRC1 are not bound at the same genomic sites, but they may come into proximity to regulate a specific subset of loci.

2) For the second suggestion, we have provided IGV snapshots, as shown in new Fig. S5b, in addition to those shown in Fig. 2a.

3) For the third suggestion, we appreciate the reviewer's comment, and this is a critical issue generally related to genome-wide colocalization studies. Completely ruling out this possibility is challenging; however, we believe that, in our case, looping is unlikely to have caused the co-localization of PRC1 and Ctcf observed in ChIP-seq analysis. Assuming the PRC1 and Ctcf co-localization is due to long-range interactions from two distal sites, with one site having only PRC1 bound and the other Ctcf. After crosslinking, the two sites stay together during the subsequent ChIP procedure; therefore, it may seem that both sites, PRC1 and Ctcf, co-localize. If this is the case, we can imagine that, if the loop is disrupted, occupancy of either PRC1 or Ctcf will disappear at some sites. However, in our Fig. EV4g and h, we conducted Ctcf ChIP-seq in Pcgf2 KO ESCs, where PRC1 loops are disrupted. We did not see changes in Ctcf enrichment at either selected loop anchors or globally. A similar observation was made in U2OS cells with siRING1B (Fig. EV4e and f). This observation suggests that the co-occupancy of PRC1 and Ctcf is unlikely to be due solely to long-range interactions.

2. Interpretation of transcriptional changes in Figure 1

In Figure 1A, the authors identify genes whose activation during neural differentiation depends on PCGF2. However, the data appear to reveal two distinct transcriptional tendencies, where some genes show reduced activation in PCGF2-deficient cells, whereas others appear to become more active.

Despite this bidirectional transcriptional behavior, the subsequent discussion largely interprets the relationship between Polycomb and CTCF in the context of transcriptional activation alone. This interpretation seems somewhat oversimplified.

Given that both activation and derepression patterns appear to be present, it would be important to consider whether the observed transcriptional effects reflect multiple regulatory modes of PcG complexes rather than a single activation mechanism.

Moreover, since the PcG-CTCF relationship is primarily analyzed in U2OS cells, the manuscript should clarify how these observations relate to the transcriptional changes observed during neural differentiation.

A more balanced discussion of these transcriptional patterns would help place the findings in a clearer mechanistic framework.

Response: We agree with the reviewer that PRC1 may regulate gene transcription bidirectionally, as indicated in Fig. 1a and in multiple previous studies. We have included a more balanced discussion of both activation and repression of genes mediated by PRC1, presumably through distinct mechanisms. It now reads "……. and NPC stages. Although canonical PRC1 complexes are traditionally viewed as transcriptional repressors, certain variants of PRC1 can also activate transcription. Consistent with the dual role of PRC1 complexes, our differential gene expression analysis revealed bidirectional changes in gene expression in Pcgf2-/- NPCs (Fig. 1a). To gain insight into the potential activation function of PRC1.2, we focused on genes that are downregulated in the absence of Pcgf2 for further analysis. Gene ontology (GO) analysis ……."

Regarding the point about the "PcG-CTCF relationship", we have performed an analysis using ChIP-seq data for PRC1 and Ctcf in ESCs (Fig. 5a). We have added in the text and figure legends to clearly state that the analysis was done in ESCs. We agree with the reviewer that it is not clear at this stage how this interaction may regulate neuronal differentiation. Therefore, we modified the text in the discussion section accordingly. It reads "…… activation upon differentiation. However, the cPRC1.2 and CTCF relationship was primarily examined in U2OS cells and ESCs, a key remaining question is how this interaction is regulated during neuronal differentiation. Future studies integrating biochemical and genetic approaches will be needed

to address how cPRC1.2 and CTCF may cooperate to achieve sophisticated gene regulation. Regarding the collaboration ……"

Minor comments

1. Chromatin organization statement (page 3, line 6)
The statement that chromatin compartments and TADs show high evolutionary conservation may be somewhat misleading depending on the references cited. TAD conservation across species remains debated and appears to depend strongly on the scale of analysis. The authors may wish to verify the cited literature and adjust the wording accordingly.

Response: We thank the reviewer for pointing out our oversight. We initially meant to say that chromatin loops are relatively more dynamic compared with TADs. To avoid any confusion, we have modified the text as suggested. It now reads "……In particular, chromatin loops constitute a dynamic, cell-type-specific regulation network. CTCF plays a pivotal role in forming…..".

2. Novelty of Figure 2
The analyses presented in Figure 2 appear largely consistent with previously reported features of PcG occupancy and chromatin states. While these data are useful for contextualizing the study, their novelty appears somewhat limited. The authors may consider clarifying whether these analyses are intended primarily as validation of previously reported observations.

Response: We agree with the reviewer that the association of PcG occupancy with chromatin loops, and we did not intend to claim this as our novel finding. However, it has not been shown that the specific cPRC1.2 is required for such loops, or its inactivation is sufficient to cause loop disruption. Furthermore, we found, to our knowledge, that the cPRC1.2 loop anchors overlap with bivalent marks and CTCF, a new finding. We have modified the text to better distinguish the known facts and our contribution. It now reads "….Despite the previously established role of PRC1 in chromatin looping, the specific subcomplex remains undefined. Given that Pcgf2 deletion had little effect …….".

3. Quantification of PcG-CTCF overlap
The manuscript frequently refers to co-localization between PcG and CTCF based on ChIP peak overlap. It would be useful if the authors could provide quantitative assessments of this overlap, for example by calculating enrichment relative to randomized genomic regions or by using

permutation-based analyses. Such analyses would help demonstrate whether the observed overlap is statistically meaningful.

Response: As the reviewer suggested, we performed a quantitative assessment using the permutation test function in the regioneR R package to evaluate the overlap between Ring1b and CTCF. The statistic suggested that they colocalized rather than a random distribution, with a p-value of 0.00099.

5. Distinguishing correlation from causation
More generally, several conclusions rely primarily on correlations between PcG binding, CTCF occupancy, and transcriptional activation. While these correlations are interesting, the manuscript would benefit from a clearer distinction between correlation and causation in the interpretation of these findings.

Response: We appreciate this comment from the reviewer and have revised the text to avoid overstatement of our conclusions. We made the following modifications:

From "Our results identify a novel role for the cPRC1.2 complex in activating neuro-ectodermal lineage genes through chromatin looping to promote NPC identity. " changed to "Our results suggest that the cPRC1.2 complex plays a role in activating neuro-ectodermal lineage genes through chromatin looping to promote NPC identity. "

From "Taken together, our results uncover a novel mechanism by which PRC1 and CTCF orchestrate chromatin looping interactions for bivalent gene activation, facilitating the activation of the neuronal differentiation program (Fig. 8). " changed to " Taken together, our results uncover a novel mechanism by which PRC1 and CTCF orchestrate chromatin looping interactions, which contributes to bivalent gene activation critical for neuronal differentiation (Fig. 8). "

1st Revision - Editorial Decision 25th Jun 2026

Dear Dr. Gao,

Thank you for the submission of your revised manuscript. We have now received the enclosed reports from the referees, and I am happy to say that both support its publication now. Both referees still have several comments that should all be addressed in the ms text. Please also remove all overstatements from the title and abstract and please write the abstract in present tense, as per journal policy.

Please also co-submit a point-by-point response to all final comments with your final ms.

A few editorial requests will also need to be addressed:

- In the Data Availability Section (DAS) only the info on deposited datasets should be listed. Statements such as "Any additional information required to reproduce data reported in this article is available from the lead contact upon request.", Lead Contact and Materials Availability sections need to be removed. The URL for the large deposited Source Data needs to be provided in the DAS. The specific URLs for datasets (Gene Expression Omnibus (GEO) under the accession number:GSE278946) need to be provided in the DAS.

- The author credits need to be removed from the ms file. All credits need to be entered during online ms submission.

- The REFERENCE format needs to be corrected: It needs to be alphabetical, not numerical; DOIs should only be used for preprints and datasets that have not been published yet. Please use the EMBO reports reference style.

- In the author checklist, please answer all questions on statistics and send us a new, completed checklist.

- A callout for Figure EV2 is missing; callouts for the Appendix tables need to be corrected in the ms and Reagents table - they are currently missing the word "Appendix".

- The APPENDIX FILE needs to be submitted as PDF format; it needs a Table of Content on the title page with page numbers, author list and affiliations are not needed; the correct nomenclature throughout the file should be Appendix Figure S1, etc. and Appendix Table S1, etc.

- The manuscript sections should be in the following order: Title page - Abstract - Introduction - Results - Discussion - Methods - Data Availability - Acknowledgments - Disclosure Statement & Competing Interests - References - Figure Legends - (Main Tables with legends if applicable) - Expanded View Figure Legends.

- Materials and Methods should be Methods

* Figure Legends - Comments *

- Please note that the exact p values are not provided in the legends of 1F; 3D; 4C,E; 5C,F; 6E; 7E,H,J; EV-1I; EV-2B,D,E; EV-5D; Figure S6-A. Please provide exact values as reasonable.
- Please indicate the statistical test used for data analysis in the legends of figures 1B,F; 3D; 4E; 6E; EV-3B,C; Figure S2-C; Figure S6-A.
- Please note that the box plots need to be defined in terms of centre, bounds of box and percentile in the legends of figures 3D; 4E; 6E; Figure S6-A.
- Please note that information related to n is missing in the legends 6E; Figure S6-A.
- Please note that the error bars are not defined in the legends of figure EV-5D.
- Please note that the measure of center for the error bars needs to be defined in the legends of figures 4C; EV-1I; EV-2B,E.

EMBO press papers are accompanied online by A) a short (1-2 sentences) summary of the findings and their significance, B) 2-3 bullet points highlighting key results and C) a synopsis image that is exactly 550 pixels wide and 300 pixels high. The synopsis image should provide a sketch of the major findings, like a graphical abstract. Please note that text needs to be readable at the final size. Please send us this information along with the final manuscript.

I look forward to seeing a final version of your manuscript as soon as possible.

Kind regards,
Esther

Esther Schnapp, PhD
Senior Editor
EMBO reports

Referee #1:

The authors have addressed my main comments. I still have some reservations about the way the systematic analysis is done, but I believe these are minor issues.

In general, the authors make the analysis needlessly obtuse, making it hard to understand the conclusions. For example, Fig 3b continues to be superficially analysed. Why no pileups? Also, the connection from the epigenetic changes (or lack thereof) to the changes in loops is unclear as the authors admit the epigenome at the cPRC2.1 loops does not change. So what is the mechanism?

Nonetheless, I can appreciate this manuscript is of narrow interest to PRC-oriented researchers, and may find an audience with them, despite my continuting lukewarm attitude to the manuscript.

Referee #2:

I appreciate the authors' efforts in revising the manuscript. The revised version has addressed many of the concerns raised in the previous round of review, and the additional genome-wide analyses have strengthened the manuscript considerably.
I acknowledge that cPRC1.2 contributes to transcriptional activation during neural differentiation, as supported by multiple lines of genetic evidence including Pcgf2 knockout, Phc1ΔSAM, and the ΔIrx3 anchor deletion. I also acknowledge that CTCF is found in the vicinity of activated genomic regions.
However, I maintain some reservations regarding the proposed model. It remains unclear whether the formation of CTCF-mediated loops is a consequence of transcriptional activation itself or a direct result of cPRC1.2 activity. Furthermore, the co-localization data between PcG and CTCF, which relies primarily on analyses in U2OS cells, does not constitute strong evidence for a functional cooperation between these factors in the ESC-to-NPC differentiation context. The mechanistic leap from co-localization to cooperative loop regulation therefore requires more cautious interpretation.
Additionally, the direct evidence for cPRC1.2 loop-dependent CTCF loop formation is currently limited to a single locus (Irx3/Irx5), and broader generalization of this model warrants careful discussion. This concern is also reflected in the Discussion, where the language remains somewhat assertive regarding the cooperative role of PRC1 and CTCF.
Overall, I recommend minor revision to moderate the mechanistic claims and provide a more balanced discussion of alternative interpretations.

Response (EMBOR-2026-63823V2**)**

Dear Dr. Schnapp,

We thank the editorial team and the reviewers for supporting our manuscript for publication in your prestigious journal. We also appreciate the valuable comments and suggestions for further revision. In response, we have carefully addressed these issues and hope you will find the revised manuscript acceptable for publication.

Please also remove all overstatements from the title and abstract and please write the abstract in present tense, as per journal policy.

We have toned down the language by replacing "Our study discovers…" with more cautious phrasing, now reading "Our results suggest…" in the abstract. In addition, we have revised all verbs in the abstract to the present tense to comply with journal policy.

Please also co-submit a point-by-point response to all final comments with your final ms.

A few editorial requests will also need to be addressed:

- In the Data Availability Section (DAS) only the info on deposited datasets should be listed. Statements such as "Any additional information required to reproduce data reported in this article is available from the lead contact upon request.", Lead Contact and Materials Availability sections need to be removed. The URL for the large deposited Source Data needs to be provided in the DAS. The specific URLs for datasets (Gene Expression Omnibus (GEO) under the accession number:GSE278946) need to be provided in the DAS.

We have removed Lead Contact and Materials Availability sections. We kept only the info on deposited datasets and added URLs for datasets (Gene Expression Omnibus (GEO) under the accession number:GSE278946).

- The author credits need to be removed from the ms file. All credits need to be entered during online ms submission.

We have made the requested revisions accordingly.

- The REFERENCE format needs to be corrected: It needs to be alphabetical, not numerical; DOIs should only be used for preprints and datasets that have not been published yet. Please use the EMBO reports reference style.

We have made the requested revisions accordingly.

- In the author checklist, please answer all questions on statistics and send us a new, completed checklist.

We have made the requested revisions accordingly.

- A callout for Figure EV2 is missing; callouts for the Appendix tables need to be corrected in the ms and Reagents table - they are currently missing the word "Appendix".

We have made the requested revisions accordingly.

- The APPENDIX FILE needs to be submitted as PDF format; it needs a Table of Content on the title page with page numbers, author list and affiliations are not needed; the correct nomenclature throughout the file should be Appendix Figure S1, etc. and Appendix Table S1, etc.

We have made the requested revisions accordingly.

- The manuscript sections should be in the following order: Title page - Abstract - Introduction - Results - Discussion - Methods - Data Availability - Acknowledgments - Disclosure Statement & Competing Interests - References - Figure Legends - (Main Tables with legends if applicable) - Expanded View Figure Legends.

We have made the requested revisions accordingly.

- Materials and Methods should be Methods

We have made the requested revisions accordingly.

* Figure Legends - Comments *

- Please note that the exact p values are not provided in the legends of 1F; 3D; 4C,E; 5C,F; 6E; 7E,H,J; EV-1I; EV-2B,D,E; EV-5D; Figure S6-A. Please provide exact values as reasonable.

We have made the requested revisions accordingly.

- Please indicate the statistical test used for data analysis in the legends of figures 1B,F; 3D; 4E; 6E; EV-3B,C; Figure S2-C; Figure S6-A.

We have made the requested revisions accordingly.

- Please note that the box plots need to be defined in terms of centre, bounds of box and percentile in the legends of figures 3D; 4E; 6E; Figure S6-A.

We have made the requested revisions accordingly.

- Please note that information related to n is missing in the legends 6E; Figure S6-A.

We have made the requested revisions accordingly.

- Please note that the error bars are not defined in the legends of figure EV-5D.

We have made the requested revisions accordingly.

- Please note that the measure of center for the error bars needs to be defined in the legends of figures 4C; EV-1I; EV-2B,E.

We have made the requested revisions accordingly.

EMBO press papers are accompanied online by A) a short (1-2 sentences) summary of the findings and their significance, B) 2-3 bullet points highlighting key results and C) a synopsis image that is exactly 550 pixels wide and 300 pixels high. The synopsis image should provide a sketch of the major findings, like a graphical abstract. Please note that text needs to be readable at the final size. Please send us this information along with the final manuscript.

We have prepared a short summary, highlights for key results, and a synopsis image to be submitted with the revised manuscript.

Referee #1:

The authors have addressed my main comments. I still have some reservations about the way the systematic analysis is done, but I believe these are minor issues.

In general, the authors make the analysis needlessly obtuse, making it hard to understand the conclusions. For example, Fig 3b continues to be superficially analysed. Why no pileups?

We appreciate the reviewer's suggestion but believe the heatmap is more informative. While a pileup plot displays the average coverage profile across all sites, it collapses site-to-site variability into a single summary line. In contrast, the heatmap presents the full dataset with read density for every individual binding site simultaneously, therefore providing greater transparency and allowing readers to see both the consistency and heterogeneity of genome-wide enrichment.

Also, the connection from the epigenetic changes (or lack thereof) to the changes in loops is unclear as the authors admit the epigenome at the cPRC2.1 loops does not change. So what is the mechanism?

As the reviewer pointed out, our new analysis reveals no substantial change in H3K27me3 or H3K4me3 enrichment at cPRC1.2 loop anchors between WT and Pcgf2/4 KO cells (Fig. 3b). This finding suggests that, while cPRC1.2 is required for these loops, the loops themselves are not a direct consequence of bivalent chromatin marks. Mechanistically, given the established role of PRC2-mediated H3K27me3 in recruiting cPRC1, we propose that H3K27me3 primarily directs cPRC1 localization to distal genomic sites, which in turn facilitates loop formation through multivalent interactions among cPRC1 subunits. Specifically, CBX binds H3K27me3 to tether cPRC1 to anchor regions, while PHC subunits bridge multiple cPRC1 complexes via SAM domain-mediated oligomerization.

Nonetheless, I can appreciate this manuscript is of narrow interest to PRC-oriented researchers, and may find an audience with them, despite my continuting lukewarm attitude to the manuscript.

Referee #2:

I appreciate the authors' efforts in revising the manuscript. The revised version has addressed many of the concerns raised in the previous round of review, and the additional genome-wide analyses have strengthened the manuscript considerably.
I acknowledge that cPRC1.2 contributes to transcriptional activation during neural differentiation, as

supported by multiple lines of genetic evidence including Pcgf2 knockout, Phc1ΔSAM, and the ΔIrx3 anchor deletion. I also acknowledge that CTCF is found in the vicinity of activated genomic regions.

However, I maintain some reservations regarding the proposed model. It remains unclear whether the formation of CTCF-mediated loops is a consequence of transcriptional activation itself or a direct result of cPRC1.2 activity. Furthermore, the co-localization data between PcG and CTCF, which relies primarily on analyses in U2OS cells, does not constitute strong evidence for a functional cooperation between these factors in the ESC-to-NPC differentiation context. The mechanistic leap from co-localization to cooperative loop regulation therefore requires more cautious interpretation.

We thank the reviewer for raising these important points and agree that our proposed model requires more cautious interpretation.

First, regarding the distinction between CTCF-mediated loop formation as a consequence of transcriptional activation versus a direct result of cPRC1.2 activity, we acknowledge that our analysis was limited to a single locus. It is possible that the loop we observed is an indirect outcome of gene activation upon differentiation, rather than a direct effect of cPRC1.2 activity.

Second, we agree that our co-localization data between PcG and CTCF, derived primarily from U2OS cells, do not constitute strong evidence for functional cooperation in the ESC-to-NPC differentiation context. We acknowledge that these data are correlative and obtained in a heterologous system, and we have accordingly toned down our interpretation.

We have therefore incorporated a more cautious discussion, which now reads: ***Furthermore, as our analysis was limited to a single loop, we cannot exclude the possibility that CTCF-mediated loop formation arises indirectly from cPRC1.2 loops, occurring as a result of gene activation upon differentiation. Hence, future research incorporating genome-wide approaches analyzing multiple differentiation stages will be key to delineate the precise sequence of molecular events following the disruption of PRC1 loops in regulating cell fate transitions.***

Additionally, the direct evidence for cPRC1.2 loop-dependent CTCF loop formation is currently limited to a single locus (Irx3/Irx5), and broader generalization of this model warrants careful discussion. This concern is also reflected in the Discussion, where the language remains somewhat assertive regarding the cooperative role of PRC1 and CTCF. Overall, I recommend minor revision to moderate the mechanistic claims and provide a more balanced discussion of alternative interpretations.

We agree with the reviewer and have accordingly toned down our language to more accurately reflect our findings. In the Results section, we have replaced ***"……our results uncover a novel mechanism"*** **with** ***"……our results suggest a mechanism……"*****. In the Discussion, we have revised** ***"Our study uncovers a novel mechanism……"*** **to** ***"Our study supports a novel mechanism……"*****.**

Dr. Zhonghua Gao
Shenzhen University of Advanced Technology
The Faculty of Synthetic Biology
518107
China

Dear Dr. Gao,

I am very pleased to accept your manuscript for publication in the next available issue of EMBO reports. Thank you for your contribution to our journal.

Your manuscript will be processed for publication by EMBO Press. It will be copy edited and you will receive page proofs prior to publication. Please note that you will be contacted by Springer Nature Author Services to complete licensing and payment information.

You may qualify for financial assistance for your publication charges - either via a Springer Nature fully open access agreement or an EMBO initiative. Check your eligibility: https://link.springer.com/journal/44319/how-to-publish-with-us

Should you be planning a Press Release on your article, please notify the production team via this form in order to coordinate publication and release dates: https://forms.cloud.microsoft/e/eH4A8JVP6g

If you have any questions, please do not hesitate to contact the Editorial Office. Thank you for your contribution to EMBO Reports.

Best regards,
Esther

Esther Schnapp, PhD
Senior Editor
EMBO reports

>>> Please note that it is EMBO Reports policy for the transcript of the editorial process (containing referee reports and your response letter) to be published as an online supplement to each paper. If you do NOT want this, you will need to inform the Editorial Office via email immediately. More information is available here: https://link.springer.com/partners/embo-press/editorial-policies#Peer%20review

EMBO Press Author Checklist

Corresponding Author Name: Zhonghua Gao	
Journal Submitted to: EMBO Reports	
Manuscript Number: EMBOR-2026-63823V2	

USEFUL LINKS FOR COMPLETING THIS FORM
The EMBO Journal - Author Guidelines
EMBO Reports - Author Guidelines
Molecular Systems Biology - Author Guidelines
EMBO Molecular Medicine - Author Guidelines

Reporting Checklist for Life Science Articles (updated January
This checklist is adapted from Materials Design Analysis Reporting (MDAR) Checklist for Authors. MDAR establishes a minimum set of requirements in transparent reporting in the life sciences (see Statement of Task: 10.31222/osf.io/9sm4x). Please follow the journal's guidelines in preparing your
Please note that a copy of this checklist will be published alongside your article.

Abridged guidelines for figures
1. Data
The data shown in figures should satisfy the following conditions:
➞ the data were obtained and processed according to the field's best practice and are presented to reflect the results of the experiments in an accurate and unbiased manner.
➞ ideally, figure panels should include only measurements that are directly comparable to each other and obtained with the same assay.
➞ plots include clearly labeled error bars for independent experiments and sample sizes. Unless justified, error bars should not be shown for technical
➞ if n<5, the individual data points from each experiment should be plotted. Any statistical test employed should be justified.
➞ Source Data should be included to report the data underlying figures according to the guidelines set out in the authorship guidelines on Data

2. Captions
Each figure caption should contain the following information, for each panel where they are relevant:
➞ a specification of the experimental system investigated (eg cell line, species name).
➞ the assay(s) and method(s) used to carry out the reported observations and measurements.
➞ an explicit mention of the biological and chemical entity(ies) that are being measured.
➞ an explicit mention of the biological and chemical entity(ies) that are altered/varied/perturbed in a controlled manner.
➞ the exact sample size (n) for each experimental group/condition, given as a number, not a range;
➞ a description of the sample collection allowing the reader to understand whether the samples represent technical or biological replicates (including how many animals, litters, cultures, etc.).
➞ a statement of how many times the experiment shown was independently replicated in the laboratory.
➞ definitions of statistical methods and measures:
- common tests, such as t-test (please specify whether paired vs. unpaired), simple χ2 tests, Wilcoxon and Mann-Whitney tests, can be unambiguously identified by name only, but more complex techniques should be described in the methods section;
- are tests one-sided or two-sided?
- are there adjustments for multiple comparisons?
- exact statistical test results, e.g., P values = x but not P values < x;
- definition of 'center values' as median or average;
- definition of error bars as s.d. or s.e.m.

Please complete ALL of the questions below.
Select "Not Applicable" only when the requested information is not relevant for your study.

Materials

Newly Created Materials	Information included in the manuscript?	In which section is the information available? (Reagents and Tools Table, Materials and Methods, Figures, Data Availability Section)
New materials and reagents need to be available; do any restrictions apply?	Yes	Reagents and Tools Table

Antibodies	Information included in the manuscript?	In which section is the information available? (Reagents and Tools Table, Materials and Methods, Figures, Data Availability Section)
For **antibodies** provide the following information:<br>- Commercial antibodies: RRID (if possible) or supplier name, catalogue number and or/clone number<br>- Non-commercial: RRID or citation	Yes	Reagents and Tools Table

DNA and RNA sequences	Information included in the manuscript?	In which section is the information available? (Reagents and Tools Table, Materials and Methods, Figures, Data Availability Section)
**Short novel DNA or RNA including primers, probes**: provide the sequences.	Yes	Reagents and Tools Table

Cell materials	Information included in the manuscript?	In which section is the information available? (Reagents and Tools Table, Materials and Methods, Figures, Data Availability Section)
**Cell lines:** Provide species information, strain. Provide accession number in repository **OR** supplier name, catalog number, clone number, and/**OR** RRID.	Yes	Reagents and Tools Table
**Primary cultures:** Provide species, strain, sex of origin, genetic modification status.	Not Applicable	
Report if the cell lines were recently **authenticated** (e.g., by STR profiling) and tested for mycoplasma contamination.	Not Applicable	

Experimental animals	Information included in the manuscript?	In which section is the information available? (Reagents and Tools Table, Materials and Methods, Figures, Data Availability Section)
**Laboratory animals or Model organisms:** Provide species, strain, sex, age, genetic modification status. Provide accession number in repository **OR** supplier name, catalog number, clone number, **OR** RRID.	Not Applicable	
**Animal observed in or captured from the field:** Provide species, sex, and age where possible.	Not Applicable	
Please detail **housing and husbandry conditions**.	Not Applicable	

Plants and microbes	Information included in the manuscript?	In which section is the information available? (Reagents and Tools Table, Materials and Methods, Figures, Data Availability Section)
**Plants:** provide species and strain, ecotype and cultivar where relevant, unique accession number if available, and source (including location for collected wild specimens).	Not Applicable	
**Microbes:** provide species and strain, unique accession number if available, and source.	Not Applicable	

Human research participants	Information included in the manuscript?	In which section is the information available? (Reagents and Tools Table, Materials and Methods, Figures, Data Availability Section)
If collected and within the bounds of privacy constraints report on age, sex and gender or ethnicity for all study participants.	Not Applicable	

Core facilities	Information included in the manuscript?	In which section is the information available? (Reagents and Tools Table, Materials and Methods, Figures, Data Availability Section)
If your work benefited from core facilities, was their service mentioned in the acknowledgments section?	Yes	Acknowledgments

Design

Study protocol	Information included in the manuscript?	In which section is the information available? (Reagents and Tools Table, Materials and Methods, Figures, Data Availability Section)
If study protocol has been **pre-registered, provide DOI in the manuscript**. For clinical trials, provide the trial registration number **OR** cite DOI.	Not Applicable	
Report the **clinical trial registration number** (at ClinicalTrials.gov or equivalent), where applicable.	Not Applicable	

Laboratory protocol	Information included in the manuscript?	In which section is the information available? (Reagents and Tools Table, Materials and Methods, Figures, Data Availability Section)
Provide DOI OR other citation details if **external detailed step-by-step protocols** are available.	Not Applicable	

Experimental study design and statistics	Information included in the manuscript?	In which section is the information available? (Reagents and Tools Table, Materials and Methods, Figures, Data Availability Section)
Include a statement about **sample size** estimate even if no statistical methods were used.	Yes	Methods
Were any steps taken to minimize the effects of subjective bias when allocating animals/samples to treatment (e.g. **randomization procedure**)? If yes, have they been described?	Yes	Methods
Include a statement about **blinding** even if no blinding was done.	Yes	Methods
Describe **inclusion/exclusion criteria** if samples or animals were excluded from the analysis. Were the criteria pre-established?<br><br>If sample or data points were omitted from analysis, report if this was due to attrition or intentional exclusion and provide justification.	Yes	Methods
For every figure, are **statistical tests** justified as appropriate? Do the data meet the assumptions of the tests (e.g., normal distribution)? Describe any methods used to assess it. Is there an estimate of variation within each group of data? **I**s the variance similar between the groups that are being statistically compared?	Yes	Methods, Figures

Sample definition and in-laboratory replication	Information included in the manuscript?	In which section is the information available? (Reagents and Tools Table, Materials and Methods, Figures, Data Availability Section)
In the figure legends: state number of times the experiment was **replicated** in laboratory.	Yes	Figures
In the figure legends: define whether data describe **technical or biological replicates**.	Yes	Figures

Ethics

Ethics	Information included in the manuscript?	In which section is the information available? (Reagents and Tools Table, Materials and Methods, Figures, Data Availability Section)
Studies involving **human participants**: State details of **authority granting ethics approval** (IRB or equivalent committee(s), provide reference number for approval.	Not Applicable	
Studies involving **human participants**: Include a statement confirming that **informed consent** was obtained from all subjects and that the experiments conformed to the principles set out in the WMA Declaration of Helsinki and the Department of Health and Human Services Belmont Report.	Not Applicable	
Studies involving **human participants:** For publication of **patient photos**, include a statement confirming that consent to publish was obtained.	Not Applicable	
Studies involving experimental **animals**: State details of **authority granting ethics approval** (IRB or equivalent committee(s), provide reference number for approval. Include a statement of compliance with ethical regulations.	Not Applicable	
Studies involving **specimen and field samples:** State if relevant **permits** obtained, provide details of authority approving study; if none were required, explain why.	Not Applicable	

Dual Use Research of Concern (DURC)	Information included in the manuscript?	In which section is the information available? (Reagents and Tools Table, Materials and Methods, Figures, Data Availability Section)
Could your study fall under dual use research restrictions? Please check biosecurity documents and list of **select agents and toxins** (CDC): https://www.selectagents.gov/sat/list.htm	Not Applicable	
If you used a select agent, is the security level of the lab appropriate and reported in the manuscript?	Not Applicable	
If a study is subject to dual use research of concern regulations, is the name of the **authority granting approval and reference number** for the regulatory approval provided in the manuscript?	Not Applicable	

Reporting

The MDAR framework recommends adoption of discipline-specific guidelines, established and endorsed through community initiatives. Journals have their own policy about requiring specific guidelines and recommendations to complement MDAR.

Adherence to community standards	Information included in the manuscript?	In which section is the information available? (Reagents and Tools Table, Materials and Methods, Figures, Data Availability Section)
State if relevant guidelines or checklists (e.g., **ICMJE, MIBBI, ARRIVE, PRISMA**) have been followed or provided.	Not Applicable	
For **tumor marker prognostic studies,** we recommend that you follow the **REMARK** reporting guidelines (see link list at top right). See author guidelines, under 'Reporting Guidelines'. Please confirm you have followed these guidelines.	Not Applicable	
For **phase II and III randomized controlled trials**, please refer to the **CONSORT** flow diagram (see link list at top right) and submit the CONSORT checklist (see link list at top right) with your submission. See author guidelines, under 'Reporting Guidelines'. Please confirm you have submitted this list.	Not Applicable	

Data Availability

Data availability	Information included in the manuscript?	In which section is the information available? (Reagents and Tools Table, Materials and Methods, Figures, Data Availability Section)
Have **primary datasets** been deposited according to the journal's guidelines (see 'Data Deposition' section) and the respective accession numbers provided in the Data Availability Section?	Yes	Data Availability Section
Were **human clinical and genomic datasets** deposited in a public access-controlled repository in accordance to ethical obligations to the patients and to the applicable consent agreement?	Not Applicable	
Are **computational models** that are central and integral to a study available without restrictions in a machine-readable form? Were the relevant accession numbers or links provided?	Not Applicable	
If publicly available data were reused, provide the respective **data citations in the reference list**.	Yes	Methods, Appendix
