# Supplementary material for: cPRC1.2 and CTCF-mediated transition from poised to active chromatin loops at bivalent genes
Source: EMBO Rep. 2026 Jul 23;27(17):5092–120. doi: 10.1038/s44319-026-00885-3 (PMC13554221; doi:10.1038/s44319-026-00885-3)
Supplement: Supplementary file 3 [file 44319_2026_885_MOESM7_ESM.pdf]

Expanded View Figures

Figure EV1. Generation and phenotypic analysis of Pcgf2$^{-/-}$ and Pcgf4$^{-/-}$ ESC lines.

(**A**) Schematic of CRISPR/Cas9-mediated deletion of Pcgf2 in ESCs. Bottom: Sanger sequencing validating the excision of Exon 2. (**B**) Schematic of CRISPR/Cas9-mediated deletion of Pcgf4 in ESCs. Bottom: Sanger sequencing validating the excision of Exon 1. (**C**) Gel electrophoresis of PCR products using primers targeting the excision region in WT and Pcgf2$^{-/-}$ ESC clone. (**D**) Immunoblotting of Pcgf2 in WT and Pcgf2$^{-/-}$ ESC clone. (**E**) Gel electrophoresis of PCR products using primers targeting the excision region in WT and Pcgf4$^{-/-}$ ESC clone. (**F**) Immunoblotting of Pcgf4 in WT and Pcgf4$^{-/-}$ ESC clone. (**G**) Schematic of NPC differentiation, as detailed in the Methods section. (**H**) Immunofluorescence staining of Neurofilament (Nfm), a neuronal marker, in neurons differentiated from WT, Pcgf2$^{-/-}$, Pcgf4$^{-/-}$ ESCs. (**I**) RT-qPCR analysis of a pluripotency (*Oct4*) and NPC (*Pax6* and *NeuroD1*) markers in WT, Pcgf2$^{-/-}$, and Pcgf4$^{-/-}$ cells at the ESC, EB, and NPC stages, normalized to *Gapdh*. All mean values of expression levels and standard deviations (error bars) were calculated from three technical replicates. From left to right, p values are as following, for *Oct4*: 0.43, 0.76, 0.98, 0.29, 0.76, 0.50, 0.19, 0.76, 0.20; for *Pax6*: 0.24, 0.73, 0.19, 0.07, 0.008, 0.09, <0.001, 0.73, 0.04; for *NeuroD1*: 0.16, 0.33, 0.84, 0.11, 0.28, 0.27, 0.003, 0.77, and 0.27, calculated by Student's t-test. *$p < 0.05$; **$p < 0.01$; ***$p < 0.001$; n.s. not significant.

© The Author(s)

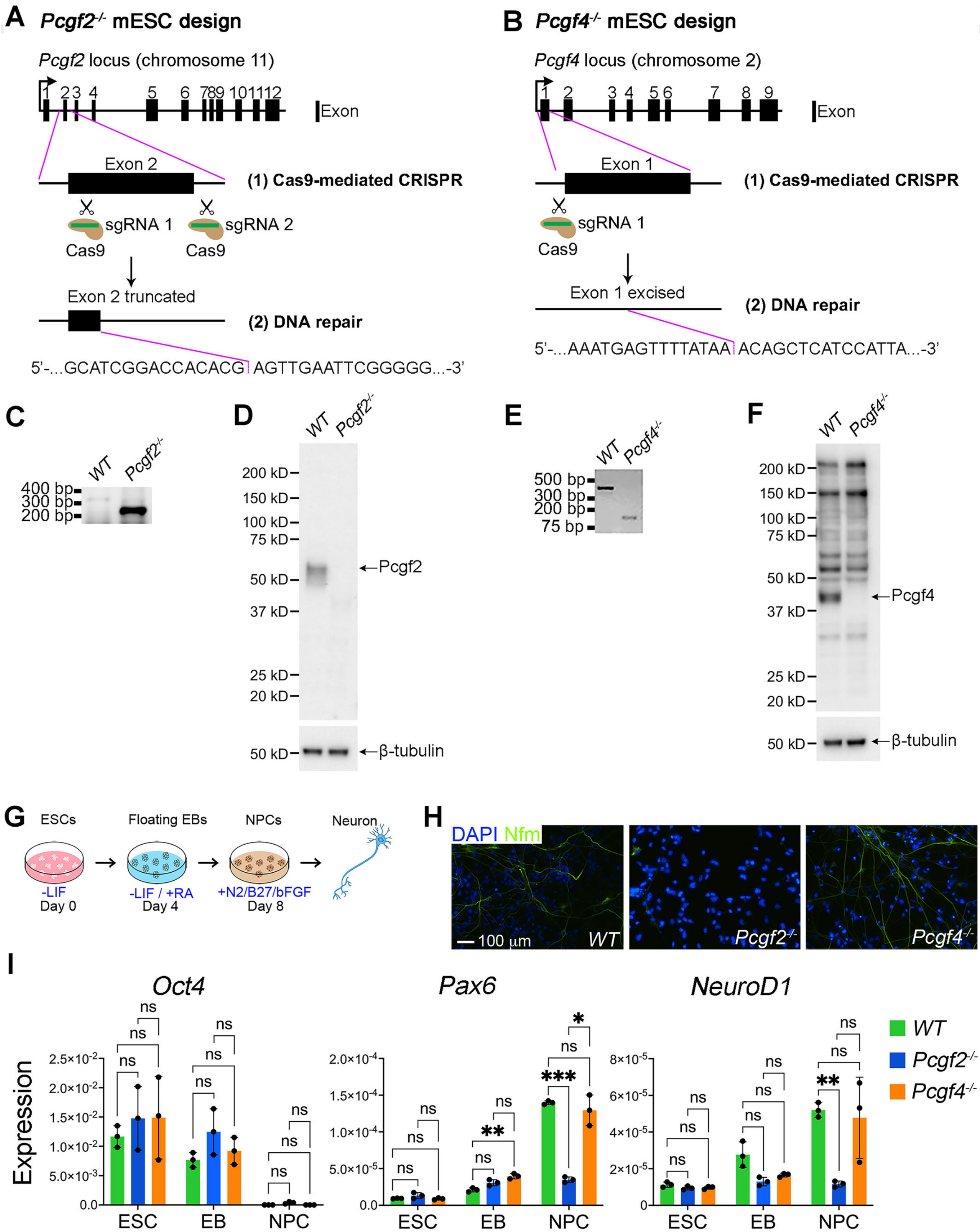
A
Pcgf2-/- mESC design
Pcgf2 locus (chromosome 11)
Exon
Exon 2
(1) Cas9-mediated CRISPR
sgRNA 1
sgRNA 2
Cas9
Cas9
Exon 2 truncated
(2) DNA repair
5'-...GCATCGGACCACACG AGTTGAATTCGGGGG...-3'
B
Pcgf4-/- mESC design
Pcgf4 locus (chromosome 2)
Exon
Exon 1
(1) Cas9-mediated CRISPR
sgRNA 1
Cas9
Exon 1 excised
(2) DNA repair
5'-...AAATGAGTTTTATAA ACAGCTCATCCATTA...-3'
C
WT
Pcgf2-/-
400 bp
300 bp
200 bp
D
WT
Pcgf2-/-
Pcgf2
β-tubulin
E
WT
Pcgf4-/-
500 bp
300 bp
200 bp
75 bp
F
WT
Pcgf4-/-
Pcgf4
β-tubulin
G
ESCs
Floating EBs
NPCs
Neuron
-LIF
Day 0
-LIF / +RA
Day 4
+N2/B27/bFGF
Day 8
H
DAPI Nfm
100 µm
WT
Pcgf2-/-
Pcgf4-/-
I
Oct4
Pax6
NeuroD1
Expression
ESC
EB
NPC
WT
Pcgf2-/-
Pcgf4-/-

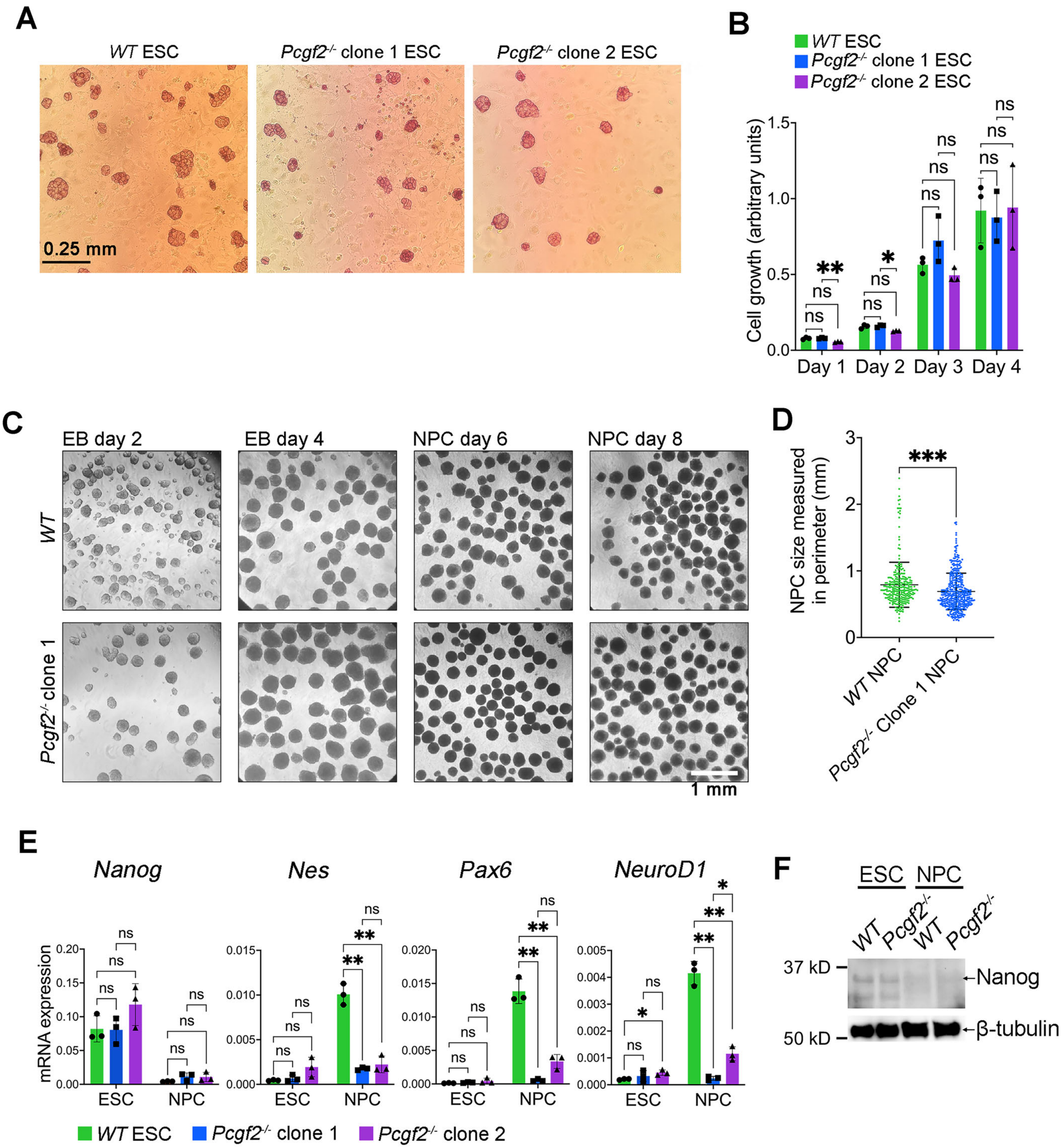
A
WT ESC
Pcgf2-/- clone 1 ESC
Pcgf2-/- clone 2 ESC
0.25 mm
B
WT ESC
Pcgf2-/- clone 1 ESC
Pcgf2-/- clone 2 ESC
Cell growth (arbitrary units)
Day 1
Day 2
Day 3
Day 4
C
EB day 2
EB day 4
NPC day 6
NPC day 8
WT
Pcgf2-/- clone 1
1 mm
D
NPC size measured in perimeter (mm)
WT NPC
Pcgf2-/- Clone 1 NPC
E
Nanog
Nes
Pax6
NeuroD1
mRNA expression
ESC
NPC
WT ESC
Pcgf2-/- clone 1
Pcgf2-/- clone 2
F
ESC
NPC
WT
Pcgf2-/-
37 kD
50 kD
Nanog
β-tubulin

 © The Author(s)

Figure EV2. Characterization of Pcgf2$^{-/-}$ ESC clones.

(**A**) Alkaline phosphatase (AP) staining in WT and two Pcgf2$^{-/-}$ ESC clones co-cultured with mouse embryonic fibroblasts (MEF). Pluripotent cells stain purple. (**B**) MTT cell proliferation assay in WT and two Pcgf2$^{-/-}$ ESC clones over four days. All mean values and standard deviations (error bars) were calculated from three technical replicates. From left to right, *p* values are: 0.97, 0.08, 0.007, 0.97, 0.16, 0.03, 0.59, 0.30, 0.21, 0.97, 0.92, 0.74. A *t*-test was performed. $^*p < 0.05$; $^{**}p < 0.01$; n.s. not significant. (**C**) Brightfield images of the WT and Pcgf2$^{-/-}$ clone 1 at EB and NPC stages during differentiation. (**D**) Quantification of NPC size at day 8 in WT ($n = 277$) and Pcgf2$^{-/-}$ clone 1 ($n = 416$) based on cell perimeter. The center line represents the mean, and the ends represent the error bars from the standard deviation. A *t*-test was performed, yielding a *p* value <0.0001. $^{***}p < 0.001$. (**E**) RT-qPCR of pluripotency (*Nanog*) and NPC (*Nes*, *Pax6*, and *NeuroD1*) markers in WT and two Pcgf2$^{-/-}$ clones at the ESC, EB, and NPC stages, normalized to *Gapdh*. All mean values of expression levels and standard deviations (error bars) were calculated from three technical replicates. From left to right, *p* values are as follows: for *Nanog*: 0.93, 0.32, 0.30, 0.25, 0.32, 0.89; for *Nes*: 0.40, 0.14, 0.32, 0.01, 0.002, 0.47; for *Pax6*: 0.13, 0.32, 0.45, 0.01, 0.005, 0.08; for *NeuroD1*: 0.41, 0.04, 0.40, 0.007, 0.003, 0.04. A *t*-test was performed. $^*p < 0.05$; $^{**}p < 0.01$; n.s. not significant. (**F**) Immunoblotting showing the Nanog protein level in WT and Pcgf2$^{-/-}$ clone 1 ESCs and NPCs.

© The Author(s)

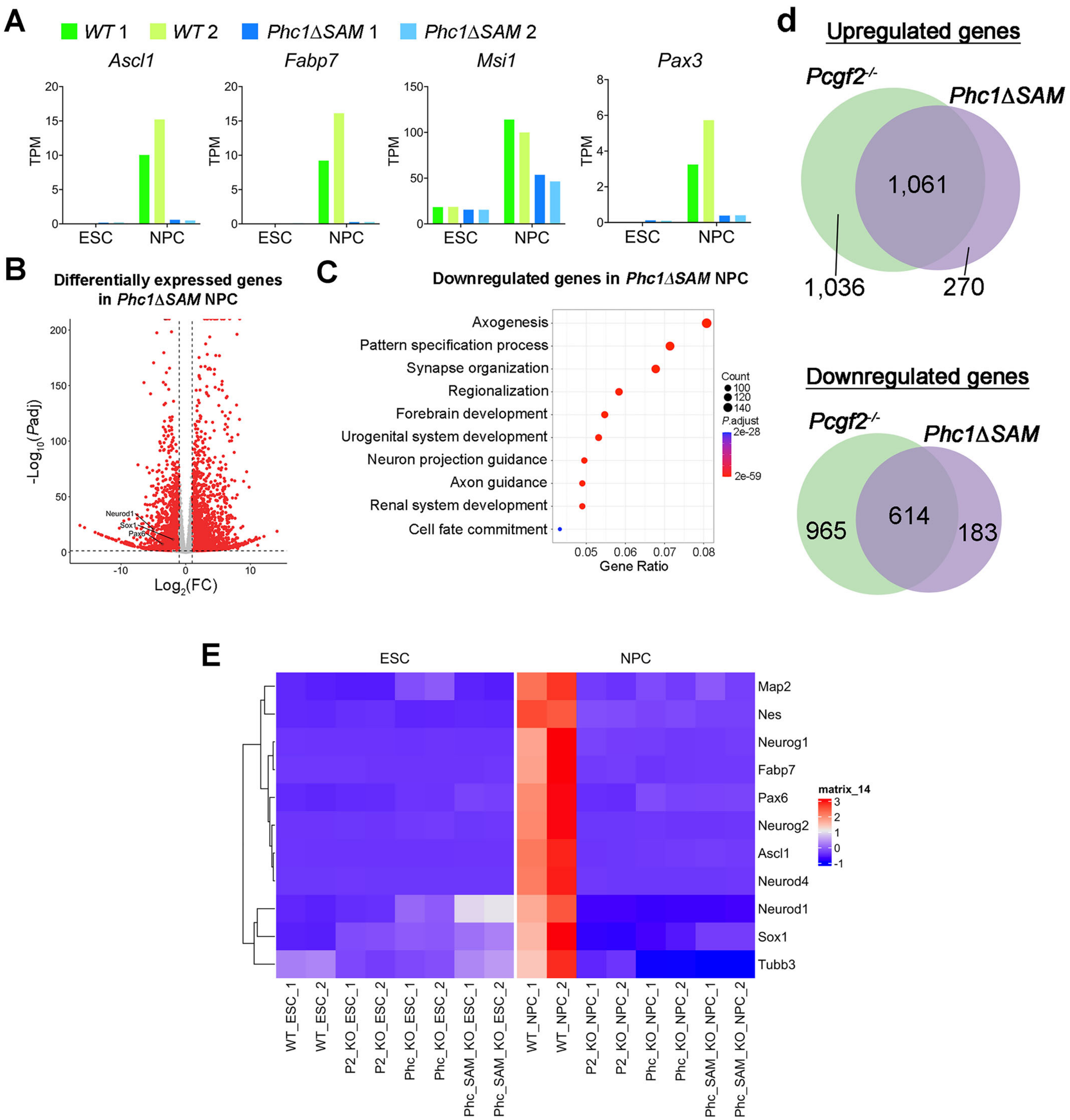

Figure EV3. SAM domain truncation alters the NPC transcriptomic profile similar to Pcgf2 deletion.

(**A**) Bar graphs show the TPM values for NPC and neuron-related gene markers in WT and Phc1ΔSAM ESCs and NPCs. (**B**) Volcano plot of differentially expressed genes (DEGs) in Phc1ΔSAM NPCs compared to WT NPCs. *p* values are calculated by Wald test using DEseq2 package. Red dots denote significant DEGs, and gray dots denote insignificant DEGs. NPC markers such as NeuroD1, Sox1, and Pax6 are indicated among the downregulated genes. (**C**) GO analysis of downregulated genes in Phc1ΔSAM NPCs. Gene ratio (x-axis) indicates the proportion of genes in each GO term. *p* values are calculated by the hypergeometric distribution embedded in the clusterprofiler package. (**D**) Venn diagrams show the number of differentially expressed genes that are either exclusive or shared between Pcgf2$^{-/-}$ and Phc1ΔSAM NPCs based on RNA-seq data. (**E**) Heatmap using RNA-seq data showing selected neuronal genes targeted by cPRC1.2 loops.

 © The Author(s)

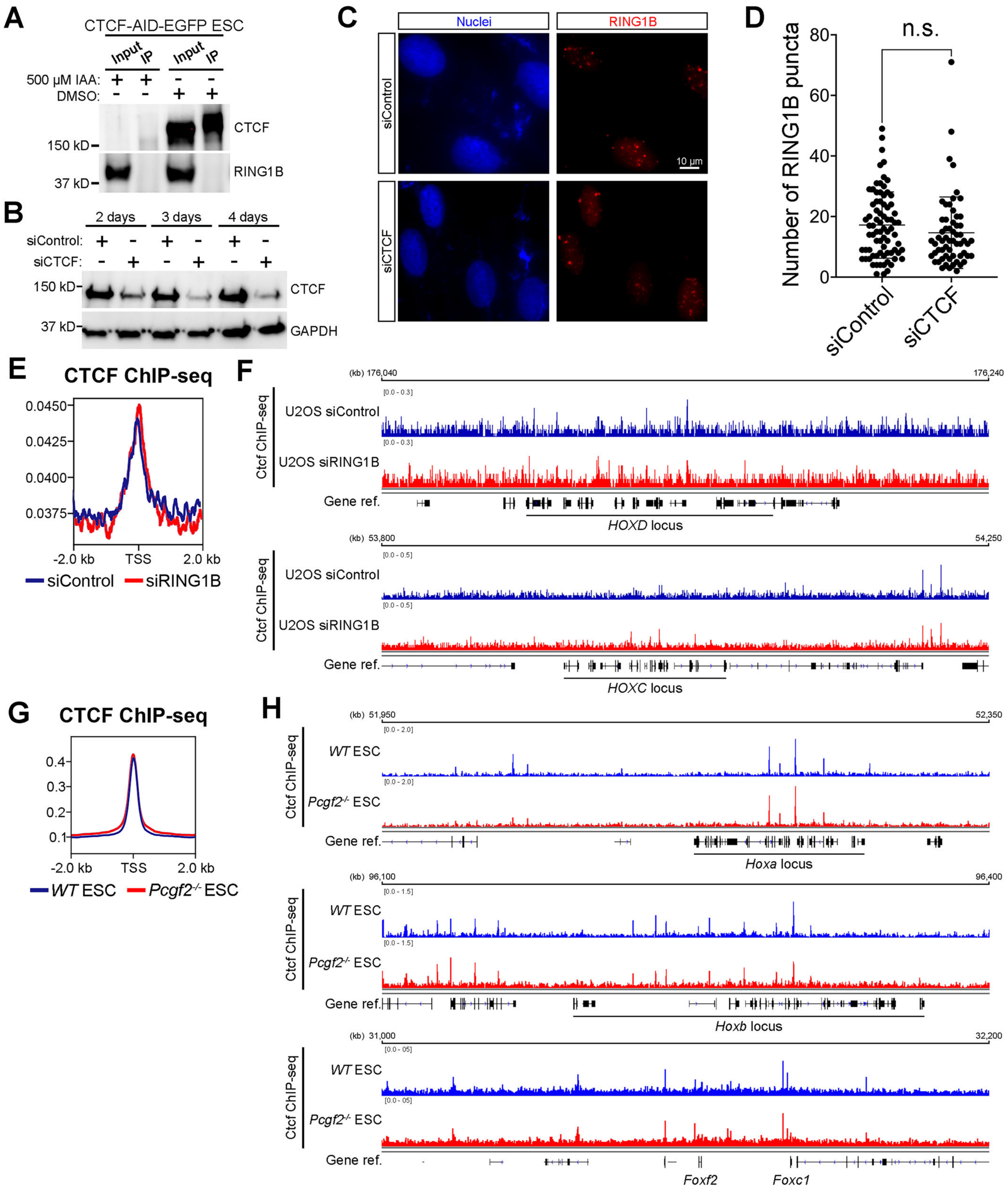
A
CTCF-AID-EGFP ESC
Input IP Input IP
500 μM IAA: + + - -
DMSO: - - + +
150 kD
37 kD
CTCF
RING1B
B
2 days 3 days 4 days
siControl: + - + - + -
siCTCF: - + - + - +
150 kD
37 kD
CTCF
GAPDH
C
Nuclei
RING1B
siControl
siCTCF
10 μm
D
n.s.
Number of RING1B puncta
siControl
siCTCF
E
CTCF ChIP-seq
-2.0 kb TSS 2.0 kb
siControl siRING1B
F
Ctcf ChIP-seq
U2OS siControl
U2OS siRING1B
Gene ref.
HOXD locus
HOXC locus
G
CTCF ChIP-seq
-2.0 kb TSS 2.0 kb
WT ESC Pcgf2-/- ESC
H
Ctcf ChIP-seq
WT ESC
Pcgf2-/- ESC
Gene ref.
Hoxa locus
Hoxb locus
Foxf2
Foxc1

Figure EV4. CTCF is not required for PcG body formation.

(**A**) Immunoprecipitation using CTCF antibody in CTCF-AID-EGFP ESCs with 500 mM indole-3-acetic acid (IAA) or DMSO treatment for 24 h, followed by immunoblotting for CTCF and RING1B. (**B**) Immunoblotting of CTCF in U2OS cells with CTCF knockdown (siCTCF) or control (siControl) over 2, 3, and 4 days. (**C**) Immunofluorescent images of siControl and siCTCF U2OS cells stained for RING1B. (**D**) Dot plots of PcG body count in siControl ($n = 77$) and siCTCF ($n = 60$) U2OS cells. The center line represents the mean, and the ends represent the error bars from the standard deviation. A *t*-test was performed. n.s. not significant. (**E**) Line graphs show the CTCF enrichment based on ChIP-seq output in siControl and siRING1B U2OS cells. (**F**) IGV graphs show the ChIP-seq tracks of CTCF enrichment in siControl and siRING1B U2OS cells at the *HOXD* (top panel) and *HOXC* (bottom panel) loci. (**G**) Line graphs show the CTCF enrichment based on ChIP-seq output in WT and Pcgf2$^{-/-}$ ESCs and NPCs. (**H**) IGV graphs show the ChIP-seq tracks of Ctcf enrichment in WT and Pcgf2$^{-/-}$ ESCs at the *Hoxa* (top panel), *Hoxb* (middle panel), and *Foxq1/Foxc1* (bottom panel) loci.

 © The Author(s)

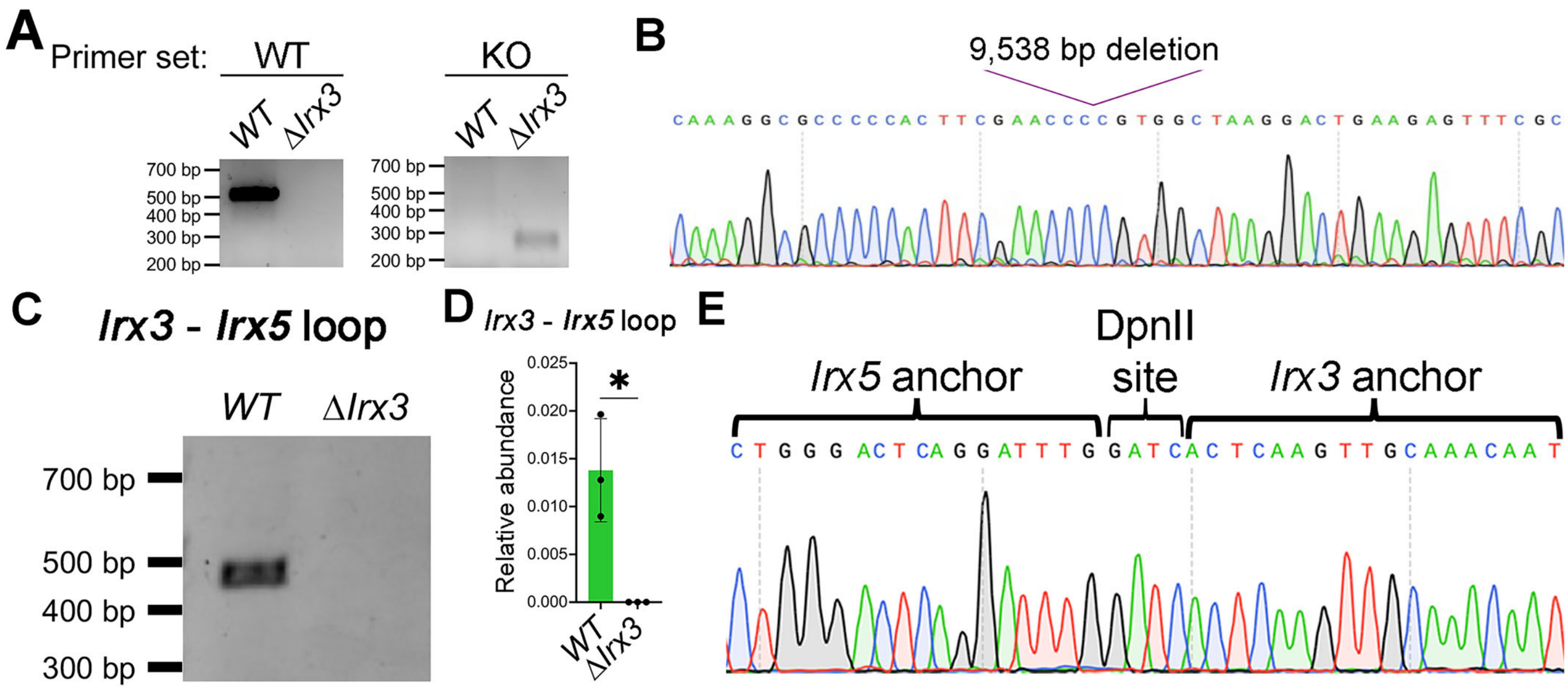

Figure EV5. Irx3 anchor deletion disrupts Irx3-Irx5 cPRC1.2 loop formation.

(**A**) Agarose gel picture shows the PCR output of Irx3 anchor deletion (ΔIrx3) in ESCs using specific primer sets to amplify the intact sequence (WT) or the excised sequence (KO). (**B**) Sanger sequencing result of the homozygous deletion of the Irx3 anchor in WT ESCs. (**C**) Agarose gel picture shows the 3C-PCR result of the cPRC1.2 Irx3-Irx5 loop interaction in WT and ΔIrx3 ESCs. (**D**) The bar graph shows the 3C-qPCR analysis for Irx3-Irx5 loop interaction between WT and ΔIrx3 ESCs. The relative abundance is calculated by normalizing against the internal loading control for each sample. Each data point represents three biological replicates. Error bars represent standard deviations. A *t*-test was performed, yielding a *p* value of 0.05. $^*p < 0.05$. (**E**) Sanger sequencing result for Irx3-Irx5 loop interaction fragment in WT ESCs derived from 3C.
